# Inhibition of PI3K and MAPK pathways along with KIT inhibitors as a strategy to overcome drug resistance in gastrointestinal stromal tumors

**DOI:** 10.1371/journal.pone.0252689

**Published:** 2021-07-29

**Authors:** Anu Gupta, Shuang Ma, Kepeng Che, Ajaybabu V. Pobbati, Brian P. Rubin

**Affiliations:** 1 Department of Cancer Biology, Lerner Research Institute, Cleveland Clinic Foundation, Cleveland, Ohio, United States of America; 2 Robert J. Tomsich Pathology and Laboratory Medicine Institute, Cleveland Clinic Foundation, Cleveland, Ohio, United States of America; Osmania University, Hyderabad, India, INDIA

## Abstract

Activating mutations in KIT/PDGFRA receptor tyrosine kinases drive gastrointestinal stromal tumors (GIST). KIT/PDGFRA inhibitors, such as imatinib do not evoke an effective cytocidal response, leaving room for quiescence and development of multiple secondary resistance mutations. As the majority of the secondary resistance clones activate PI3K and MAPK pathways, we investigated whether combined targeting of KIT/PI3K/MAPK (KPM) pathways overcomes drug resistance and quiescence in GIST cells. We monitored the proliferation of imatinib–sensitive and–resistant GIST cell lines after treating them with various combinations of drugs to inhibit KPM pathways. Cytocidal response was evaluated through proliferation, apoptosis and colony outgrowth assays. Combined inhibition of KPM signaling pathways using a KPM inhibitor cocktail decreased the survival of drug-resistant GIST cells and dramatically reduced their proliferation. Downstream pathway analysis showed that the residual PI3K/MAPK signaling observed after KIT inhibitor treatment plays a role in mediating quiescence and drug resistance. The KPM inhibitor cocktail with sunitinib or regorafenib effectively induced apoptosis and prevented colony outgrowth after long-term drug removal, suggesting that it can be used as an effective strategy against quiescence and drug resistance in metastatic GIST.

## Introduction

Gastrointestinal stromal tumors (GIST) are the most common mesenchymal neoplasms of the gastrointestinal tract [1, 2]. GIST arise from interstitial cells of Cajal (peristaltic pacemaker cells of the gut) or from their stem cell-like precursors as they accumulate primary mutations [3]. The most common primary alterations that drive GIST pathogenesis are activating mutations that occur in a mutually exclusive manner in the two receptor tyrosine kinases KIT and platelet-derived growth factor receptor alpha (PDGFRA) [4–6].

Imatinib mesylate (IM), a small molecule tyrosine kinase inhibitor that abrogates KIT and PDGFRA activity, is a highly effective treatment for KIT/PDGFRA -driven metastatic GIST and leads to stable disease in approximately 85% of cases [7, 8]. IM administration for GISTs is the first example of using a targeted therapy to manage a solid tumor. Although IM revolutionized the treatment of unresectable or metastatic GIST with primary KIT and PDGFRA mutations, prolonged IM treatment is required to prevent the re-activation of quiescent nonproliferating tumor cells, since IM is cytostatic and not cytocidal. These quiescent GIST cells present a clinical challenge as they are resistant to apoptosis and endure drug treatment by undergoing autophagy [9].

Although, imatinib has shown good efficacy against primary mutations in exon 11, it is not effective against primary A-loop mutations of PDGFRA (D842V) which occurs in ~5% of the GIST patients [10]. A corresponding mutation in c-KIT (D816V) also provides primary resistance to imatinib. Prolonged exposure to IM results in secondary resistance mutations after approximately 18–24 months of treatment [11]. Tumors with secondary mutations are then treated with sunitinib (SU) [12], a second-line therapy and Regorafenib (RE), a third-line therapy for IM- and SU-resistant tumors [13]. Although SU or RE treatment results in an increase in the median time to disease progression, resistance usually occurs within 6–12 months for each line of therapy. Ripretinib has been recently approved as 4^th^ line of therapy for patients who progress after three lines of therapies. Ripretinib treated patients have a mean progression-free survival of 6.3 months, compared to 1 month for placebo [14].

The challenge in long-term management of GIST is due to the occurrence of tumor heterogeneity under pharmacological pressure where tumors bear multiple secondary resistance clones that respond variably to each drug [15, 16]. Most of the inhibitors used to treat imatinib-resistant GIST in the clinic either target exon 13/14 mutations such as V654A and T670I (e.g. Sunitinib) or exon 17 mutations such as D816V (Regorafenib), but not both [17]. As a result, secondary resistant mutations arise in different loops of kinase domain upon treatment. Currently, administration of alternating therapy with SU and RE is being evaluated as an option to manage metastatic GIST [18]. Ripretinib has a broad-spectrum activity against several primary and secondary resistant mutations but is still cytostatic [19, 20]. Although potent KIT inhibitors targeting multiple resistant mutations are being tested in preclinical studies and in clinic, they are not expected to cover the entire mutational landscape [21, 22]. Genomic data on GIST also show that mutations in other key signaling proteins, such as KRAS, BRAF, PIK3CA, PTEN could also mediate resistance to KIT/PDGFRA inhibitors [23–25]. Activation of the MAPK signaling pathway through alternate receptor tyrosine kinases (RTKs), such as FGFR1 or FGFR2, AXL and c-MET can also induce resistance to IM treatment [26, 27]. Therefore, novel therapeutic approaches are needed to address the cytostatic response, heterogeneity of drug resistance mutations, and alternate/downstream pathway activation that make tumors resistant to KIT inhibitors.

Two pathways that play a crucial role in KIT/PDGFRA-mediated signaling are Phosphatidylinositol 3-kinase (PI3K) and mitogen activated protein kinase (MAPK) pathways. PI3K/AKT pathway is central to constitutively activated KIT/PDGFRA and is considered to promote proliferation of GIST [28]. RAS-RAF-MAPK pathway is another KIT/PDGFRA-activated pathway that is important for cell survival. KIT activates RAS via adaptor proteins, which in turn initiates the RAF/MEK/MAPK kinase cascade, eventually leading to upregulation of cell survival/proliferation pathways. Since, the majority of KIT–dependent and–independent mutant clones activate the PI3K/MAPK pathways, we hypothesized that inhibiting the PI3K/MAPK pathways together with KIT inhibition could be a potential therapy for IM/SU/RE–refractory GIST. Since KIT inhibitors are mostly cytostatic, we also tested whether this combinatorial treatment could induce a cytotoxic response in GIST cells and prevent the emergence of resistance.

GDC-0941 (GD) was chosen as a PI3K inhibitor as it inhibits all class I PI3Ks and mTOR. PD 0325901 (PD) and trametinib (TR) were chosen for inhibition of MAPK pathway. These inhibitors were used either alone or in combination with KIT inhibitors in cellular assays of proliferation and apoptosis. Both drug–sensitive and -resistant GIST cell lines were used to monitor if the cytostatic response of KIT inhibitors can be converted to a cytocidal response. Our results show that the KPM (KIT/PI3K/MAPK) inhibitor cocktails inhibit the survival of drug-resistant GIST cell lines and also induce apoptosis, abrogating long-term colony growth after drug removal.

## Materials and methods

### Cell lines

GIST-T1 is a cell line established from an untreated GIST harboring an IM-sensitive, KIT primary (exon 11) mutation [29]. GIST-T1 cells were obtained from Prof. Takahiro Taguchi, Kochi Medical school, Japan and were cultured as described previously [29]. GIST-T1/670 was derived (in Rubin Laboratory) from GIST-T1 as an IM-resistant clone that arose by continuous culture in 5 μM IM. In addition to the original KIT exon 11 deletion, these cells acquired a missense T670I mutation in exon 14—a mutation frequently found in GIST that have acquired secondary resistance to IM [30, 31]. GIST-T1/10R grew as a colony in 10 μM of imatinib (derived in Rubin laboratory from GIST-T1) and does not have any additional mutation in KIT or PDGFRA. GIST882 (K642E mutation), GIST430 (heterozygous primary deletion mutation in ex 11) and GIST430/654 (ex 11 and V654A mutation) were a gift from Jonathan Fletcher. MCF7 and MRC5 were purchased from ATCC. All cell lines were cultured in DMEM with 10% FBS, penicillin and streptomycin.

### Chemicals

Axitinib, cyclopamine, Erlotinib, Fasudil, Genistein, Irinotecan, Lestaurtinib, Temsirolimus, SP600125, Vatalanib, Tozasertib, Sunitinib, Dasatinib, Imatinib, Sorafenib, Nilotinib, Masitinib, PD325901 and GDC0941 were purchased from LC labs (Woburn, MA). Triptolide, 5-iodotubercidin, Calphostin C, Splitomycin, SB203580, Juglone, Thalidomide, Indirubin-3’monoxime, Compound C, Jak Inhibitor 1, Lavendustin A and FH535 were purchased from EMD (Gibbstown, NJ). Pazopanib and Regorafenib were purchased from Selleck chemicals (Houston, TX). H-89 and Rps-Be-cAMPS were purchased from Santa Cruz Biotech (Santa cruz, CA). Digitoxin was purchased from Sigma-Aldrich (St.Louis MO).

### Measuring activated RAS in GIST cell lines

Activated Ras levels were measured using active Ras kit from Cell Signaling (Danvers, MA). Briefly, cells were treated with Imatinib (1μM) or Sorafenib (500 nM) for 4 hours and lysates were incubated with GST-Raf1-RBD fusion protein to bind the activated form of GTP-bound Ras, which was then immunoprecipitated with glutathione resin as per manufacturer’s instructions. Active Ras levels were determined by western using anti-Ras Mouse mAb.

### Cell proliferation assays

A drug screen was performed in which ~40 drugs targeting different signaling were tested on the four GIST cell lines. For most of the drugs, selection was based on potential clinical relevance of their targets. Drug stocks were dissolved in DMSO, ethanol or PBS and stored in aliquots at appropriate temperature as per vendor’s instructions. For each cell line, 10,000 cells were seeded in 96-well plates and allowed to attach for 24 hours. Cells were treated with various inhibitors listed in Table 1 with a 6-point dilution range according to published effective doses of these drugs. Cellular proliferation was evaluated after 72 h of treatment using Cell Counting Kit-8 (Dojindo Laboratories, Kumamoto, Japan) according to manufacturer’s instructions. After drug treatment, WST-8, was added to each well and incubated for an additional 2 h at 37°C. The relative number of viable cells in triplicate wells were measured as absorbance (450 nm) of reduced WST-8. A compound was considered effective if it reduced the cell viability >50% at a drug concentration of <100 nM in all four cell lines. To find out the optimal concentration of GD and PD to be used for combination studies, a matrix of various concentrations (125 nM-5000 nM) of GD and PD was used to measure proliferation inhibition and the Loewe synergy score was calculated with Combenefit software.

**Table 1:** IC50s of inhibitors used for proliferation assay.

		IC_50_ (uM)					
**Drug**	**Targets**	**GIST-T1**	**T1/670**	**T1-10R**	**GIST882**	**MCF7**	**MRC5**
**Axitinib**	**VEGFR1,R2,R3, PDGFR, c-kit**	**<0.001**	**0.5**	**0.5**	**<0.01**	**>10**	**>10**
**cyclopamine**	**hedgehog (Hh)**	**>10**	**>10**	**>10**	**>10**	**n.d**	**n.d**
**Erlotinib**	**EGFR**	**>10**	**>10**	**>10**	**>10**	**n.d**	**n.d**
**Fasudil**	**Rho associated kinase II, ROCK**	**~70**	**~70**	**~70**	**~70**	**n.d**	**n.d**
**Genistein**	**NFkB, ERK**	**>50**	**>50**	**>50**	**>50**	**n.d**	**n.d**
**Irinotecan**	**Topoisomerase I inhibitor**	**0.5**	**5**	**5**	**0.5**	**5**	**5**
**Lestaurtinib**	**Several TK including flt3 and trkA, JAK2**	**0.1**	**5**	**5**	**>10**	**>10**	**>10**
**Temsirolimus**	**mTOR**	**>100**	**>100**	**>100**	**>100**	**n.d.**	**n.d.**
**SP600125**	**JNK**	**1**	**1**	**1**	**1**	**n.d.**	**n.d.**
**Vatalanib**	**VEGFR1,R2, PDGFR, c-kit, fms**	**0.1**	**>10**	**>10**	**1**	**>10**	**>10**
**Triptolide**	**NFkB, Smad, HSP, disintegrin, MMP**	**<0.01**	**<0.025**	**<0.025**	**<0.01**	**>10**	**0.01**
**5-iodotubercidin**	**NFkB, ERK2, AMPK, adenosine kinase**	**≥5**	**≥5**	**≥5**	**≥5**	**n.d**	**n.d**
**Calphostin C**	**PKC**	**0.1**	**>1**	**>1**	**0.1**	**n.d**	**n.d**
**Splitomicin**	**Histone deacetylase, Sir2**	**>10**	**>10**	**>10**	**15**	**>10**	**>10**
**SB203580**	**p38, p40, CSBP or MAP kinase homolog RK**	**>10**	**>10**	**>10**	**>10**	**n.d**	**n.d**
**Juglone**	**Cysteine rich proteases, PPIases,**	**5**	**5**	**5**	**5**	**n.d**	**n.d**
**Thalidomide**	**angiogenesis**	**>100**	**>100**	**>100**	**>100**	**n.d**	**n.d**
**Indirubin-3’-Monoxime**	**CDK**	**>10**	**>10**	**>10**	**>10**	**n.d**	**n.d**
**Tozasertib**	**aurora kinases, abl, IM resistant mutations**	**5**	**>10**	**>10**	**5**	**5**	**5**
**Nilotinib**	**VEGFR1,R2,R3, PDGFR, c-kit**	**0.025**	**>10**	**>10**	**0.025**	**n.d.**	**n.d.**
**Sunitinib**	**VEGFR1,R2,R3, PDGFR, c-kit**	**0.025**	**>10**	**>10**	**<0.2**	**n.d.**	**n.d.**
**Dasatinib**	**VEGFR1,R2,R3, PDGFR, c-kit, bcr-abl**	**0.025**	**>10**	**>10**	**<0.1**	**n.d.**	**n.d.**
**Pazopanib**	**VEGFR1,R2,R3, PDGFR, c-kit,**	**0.025**	**>5**	**>1**	**0.5**	**>10**	**>10**
**Sorafenib**	**VEGFR1,R2,R3, PDGFR, c-kit, raf, mnk2**	**0.025**	**<0.5**	**0.2**	**<0.1**	**n.d.**	**n.d.**
**JAK Inhibitor 1**	**JAK inhibitor**	**>10**	**>15**	**>15**	**>10**	**n.d.**	**n.d.**
**Lavendustin A**	**EGF, PDGF, IGF1**	**>100**	**>15**	**>15**	**>100**	**n.d.**	**n.d.**
**FH535**	**catenin/Tcf**	**>30**	**>15**	**>15**	**>30**	**n.d.**	**n.d.**
**Imatinib**	**VEGFR1,R2,R3, PDGFR, c-kit**	**<0.1**	**>10**	**>10**	**<0.1**	**>10**	**>10**
**Sarcatanib**	**src, bcr-abl, VEGFR2, FGFR, c-Kit, Aur-3**	**5**	**>10**	**>10**	**1**	**n.d**	**n.d**
**Digitoxin**	**target unknown**	**<0.1**	**0.5**	**0.5**	**<0.5**	**n.d**	**n.d**
**PRT06070**	**Syk Inhibitor**	**0.2**	**0.2**	**0.2**	**0.2**	**n.d**	**n.d**
**Regorafenib**	**VEGFR1,R2,R3, PDGFR, c-kit, RAF**	**<0.1**	**1**	**1**	**<0.1**	**>10**	**>10**
**Compound C**	**AMPK**	**<0.1**	**5**	**5**	**<0.1**	**n.d.**	**n.d.**
**Masatinib**	**abl, PDGFR, c-kit, c-fms**	**0.025**	**5**	**5**	**0.05**	**n.d.**	**n.d.**
**PD325901**	**MEK inh**	**0.5**	**1**	**1**	**0.5**	**n.d.**	**n.d.**
**GDC-0941**	**PI3K**	**0.3**	**>1**	**>1**	**0.5**	**n.d.**	**n.d.**
**H-89**	**PKA inh**	**>25**	**n.d.**	**n.d.**	**>25**	**n.d.**	**n.d.**
**Rps-Br-cAMPS**	**PKA inh**	**>100**	**n.d.**	**n.d.**	**>100**	**n.d.**	**n.d.**

IC_50_ values from cell viability assays after 72 hours of treatment with 9-point dilution of inhibitors. n.d., not done.

### Apoptosis assays

Around 10,000 cells/well were plated in complete media in 96-well white flat-bottom plates and therapeutic compounds were added 24 hours later. Apoptosis was evaluated by measuring caspase-3/7 activity using Caspase-Glo 3/7 Assay System (Promega, WI, USA) after 24 and 48 h of treatment with inhibitors. Samples (100 μl) were gently mixed with Caspase-Glo substrate (100 μl) and the luminescence of each sample in triplicate wells was measured using a Wallac 1420 multi-label counter (Perkin Elmer, MA, USA) after one hour of incubation. Inhibitor concentrations used were as mentioned in figure legends.

### Immunoblot analysis

Cells were treated with inhibitors for 24 hours and then lysed in RIPA buffer (1% Triton X-100, 1% sodium deoxycholate, 0.1% SDS, 25 mM Tris, pH 7.6, 150mM NaCl, 10 mM NaF, 10 mM β-glycerophosphate, 1 mM Na_3_VO_3_, and 10 nM calyculin A) plus protease inhibitors. Inhibitors were used at concentrations as indicated in Figure legends. The following primary antibodies p-KIT (Tyr 719), p-AKT (Ser 473), p-S6 (Ser235/236), p-ERK (Thr202/Tyr204), ERK, S6, AKT, PARP (Cell Signaling, Danvers, MA, USA) beta-actin (Sigma-Aldrich, MO, USA) and, c-KIT (Santa Cruz Biotech, CA, USA) were used for immunoblot analysis.

### Clonogenic assays

In 6 well plates, 500 cells were plated/well (in triplicate) for GIST-T1 and GIST-T1/10R and 1000 cells for GIST-T1/670 and allowed to attach for 24 h. Cells were treated with either IM (1 μM), SU (1 μM), RE (1 μM), GDC (500 nM), PD (500 nM), trametinib (500 nM), either alone or in combination for 1 month with replacement of fresh media with drug every week. Thereafter, cells were washed with PBS twice and fresh media without inhibitors was added and colonies were allowed to grow for 2 more weeks. The colonies were stained with crystal violet and counted. Untreated wells were not revived for extra 2 weeks.

### Statistical analysis

The results were expressed as the mean ± standard error of triplicates. Multiple comparison analysis was done by 2-Way Anova to compare the significance between single agent treatment and the combination using graphpad prism version 8.4.3. P<0.05 was considered to indicate a statistically significant difference.

## Results

### Imatinib-resistant GIST cells have activated MAPK pathway

It has been previously shown that GIST cells undergo cytostatic response to KIT inhibitors, which is a major cause of progression in patients [32]. We have also shown that GIST cells can enter and remain in a quiescent phase in the presence of imatinib for long periods of time. If the drug is washed off, these cells re-enter the cell cycle and start proliferating again and if cultured continuously with imatinib, resistance develops [9]. In order to identify if any inhibitors or a combination thereof, can induce apoptosis in GIST cells, a small screen was performed to test the sensitivities of imatinib -sensitive and -resistant cell lines. A panel of ~40 clinically-used or investigational drugs were selected from multiple oncology-related drug classes, including several tyrosine kinase inhibitors, Rho, mTOR, Topoisomerase I, NFkB, CDK, Cysteine protease, JNK, JAK, TRKA, PI3K, MEK and other kinase inhibitors (Table 1). All these inhibitors were used for proliferation assays in 4 GIST cell lines with a 6-point concentration range and IC_50_s were calculated (Table 1). GIST882 and GIST-T1 are imatinib sensitive cell lines, whereas GIST-T1/670 and GIST-T1/10R are resistant to imatinib. GIST-T1/670 has acquired a T670I gatekeeper mutation and as expected is sensitive to sunitinib which targets exon 13 (V654A) and exon 14 (T670I) mutations and GIST-T1/10R is resistant to all three KIT inhibitors (S1A Fig). MCF7 (a breast cancer cell line, not dependent on KIT pathway) and MRC5 (normal fibroblast cell line) were used as control cell lines. Any compounds that inhibited the proliferation of MCF7 and MRC5 were considered non-specific and were not selected for further analysis.

**Fig 1:**
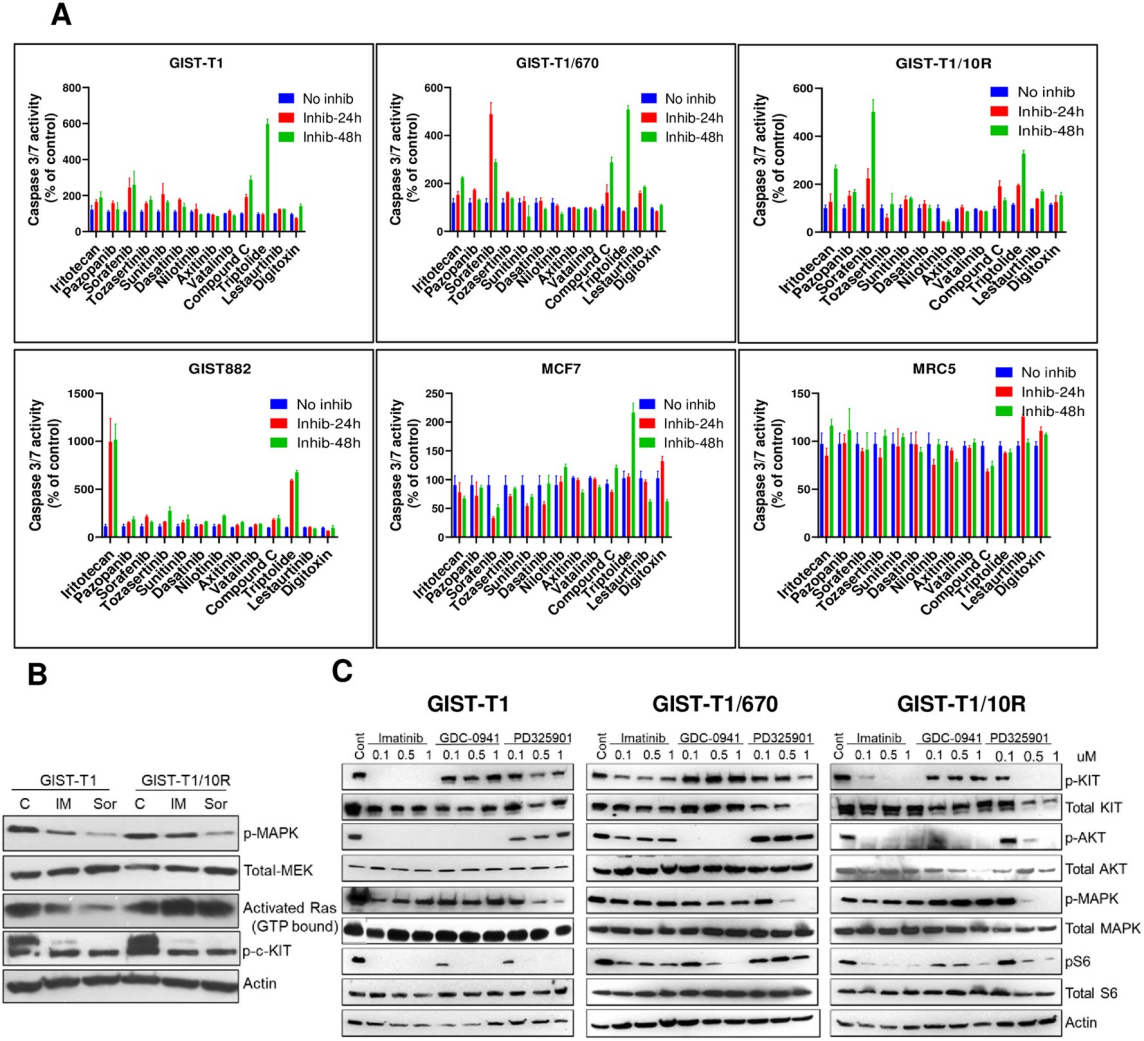
Imatinib resistant cell lines have activated MAPK pathway. (A) Caspase 3/7 activity of selected compounds in imatinib-sensitive and -resistant GIST cell lines. Cells lines were treated with irinotecan (0.5 μM), pazopanib, tozasertinib, sunitinib, dasatinib, nilotinib, vatalinib, lestauritinib, digitoxin (1 μM), sorafenib (500 nM), axitinib (500 nM), compound C (5 μM), or triptolide (25 nM) for 24 h and 48 h and caspase 3/7 activity was measured. The data is represented as % of control; each bar represents the mean of triplicates. (B) Constitutively active Ras in GIST-T1/10R is not inhibited by KIT inhibitors. GIST-T1 and GIST-T1/10R cell lines were treated with 1 μM of imatinib and 500 nM of sorafenib and activated RAS and MAPK levels were measured by western blot. (C) Dose response of imatinib, GD and PD in GIST cell lines. Cells were treated with inhibitors at indicated concentrations for 24 h. Cell lysates from GIST-T1, GIST-T1/670 and GIST-T1/10R cells were analyzed by western blot to detect the total and phosphorylated levels of the indicated proteins.

Most of the kinase inhibitors that target KIT, inhibited the cell proliferation of GIST cell lines significantly. Other inhibitors, such as irinotecan, lestaurtinib, triptolide, digitoxin, calphostin C, PRT06070, compound C, GDC-0941 and PD325901 inhibited the proliferation of imatinib sensitive cell lines at a concentration of <0.5 μM. Higher concentrations (1–5 μM) were needed to inhibit the proliferation of imatinib-resistant cell lines. All the inhibitors that showed significant inhibition of cell growth in all 4 GIST cell lines, specifically in IM-resistant cell line GIST-T1/T670I and GIST-T1/10R were evaluated by a caspase assay to monitor if these drugs induced apoptosis in GIST cell lines (Fig 1A). Irinotecan, compound C, sorafenib and triptolide showed cytotoxic effects in both imatinib sensitive and resistant cell lines by 2 fold or more. Triptolide also showed cytotoxicity in MCF7, so it was not chosen for further experiments (Fig 1A).

Sorafenib was of interest because it increased apoptosis by ~4–8 fold in both imatinib-resistant cell lines. Sorafenib is a dual inhibitor of KIT and RAF and since, GIST-T1/670 is still dependent on KIT, inhibition of both KIT and downstream MAPK pathway appeared to be cytotoxic for this cell line. Increased apoptosis in GIST-T1/10R also implies that it has become dependent on MAPK pathway. Western analysis for GTP-bound RAS showed that GIST-T1/10R has high levels of activated RAS compared to GIST-T1, even in the presence of imatinib and sorafenib (Fig 1B). Since MAPK is downstream of RAF, MEK inhibition was still observed. These results confirm that this cell line is dependent on MAPK pathway and inhibition of MAPK pathway by sorafenib induces apoptosis in this cell line (Fig 1B). As sorafenib is a multikinase inhibitor, MEKi in combination with FDA approved KIT inhibitors was used to confirm that apoptosis observed in cells is indeed due to inhibition of MAPK pathway. Since RAS pathway cross-talks with PI3 kinase pathway, GDC-0941 (already on drug panel), an FDA approved PI3K inhibitor, was also included in subsequent studies.

To determine the optimal inhibitory concentrations to be used for further experiments, a multi-dose combination matrix was used for GD and PD combinations in GIST-T1 and IC50s (Table 1) and synergy (S1B Fig) was calculated. Any combination that showed a score of 10 or greater was considered highly synergistic. Surface mapping of the combination dose response showed strong synergy at 500 nM concentration of GD with 125 nM– 500 nM of PD (S1B Fig).

To validate that the selected concentrations are sufficient to inhibit their respective targets in GIST cell lines, cells were treated with a range of GD and PD concentrations (100 nM, 500 nM and 1 μM) and p-Akt and p-MAPK levels were measured. Western blot analysis shows that GD inhibits phosphorylation of Akt at 100 nM in all cell lines tested and a complete inhibition of mTOR (as determined by p-S6) is seen only in GIST-T1 (Fig 1C and S1C Fig). Higher concentrations of GD are needed to completely inhibit mTOR signaling in resistant cell lines. 100 nM of PD is not sufficient to inhibit the p-MAPK in any of the cell lines tested. 500 nM of PD inhibits the p-MAPK by ~80% in all the cell lines. Based on cell proliferation IC_50_, synergy matrix and target engagement data, 500 nM of GD and 500 nM of PD were selected for subsequent experiments presented in this study. In a dose response study in GIST882, 100 nM of imatinib was not sufficient to completely inhibit p-KIT (S1C Fig) and no significant difference in colony outgrowth was observed at 100 nM or 1 μM of imatinib in GIST-T1 (S1D Fig). Based on these results and the IC_50_ of imatinib in GIST-T1/670 and GIST-T1/10R cell lines, 1 μM of imatinib was selected for all the combination experiments.

### KIT and PI3K/MAPK inhibitor combination significantly affects the proliferation of imatinib-resistant GIST cell lines

Administration of imatinib (IM) stabilizes GIST tumors but surviving GIST cells eventually develop resistance and proliferate, even in the presence of drug. We investigated whether the combination of MAPK and/or PI3K pathway inhibitors with IM potentiates a reduction of cell proliferation, compared to IM treatment alone. Six different GIST cell lines that have varying degrees of sensitivity/resistance to IM were used in this study. GIST-T1, GIST882, GIST430 are IM-sensitive cell lines, whereas GIST430/654, GIST-T1/670 and GIST-T1/10R are IM-resistant cell lines.

**Fig 2:**
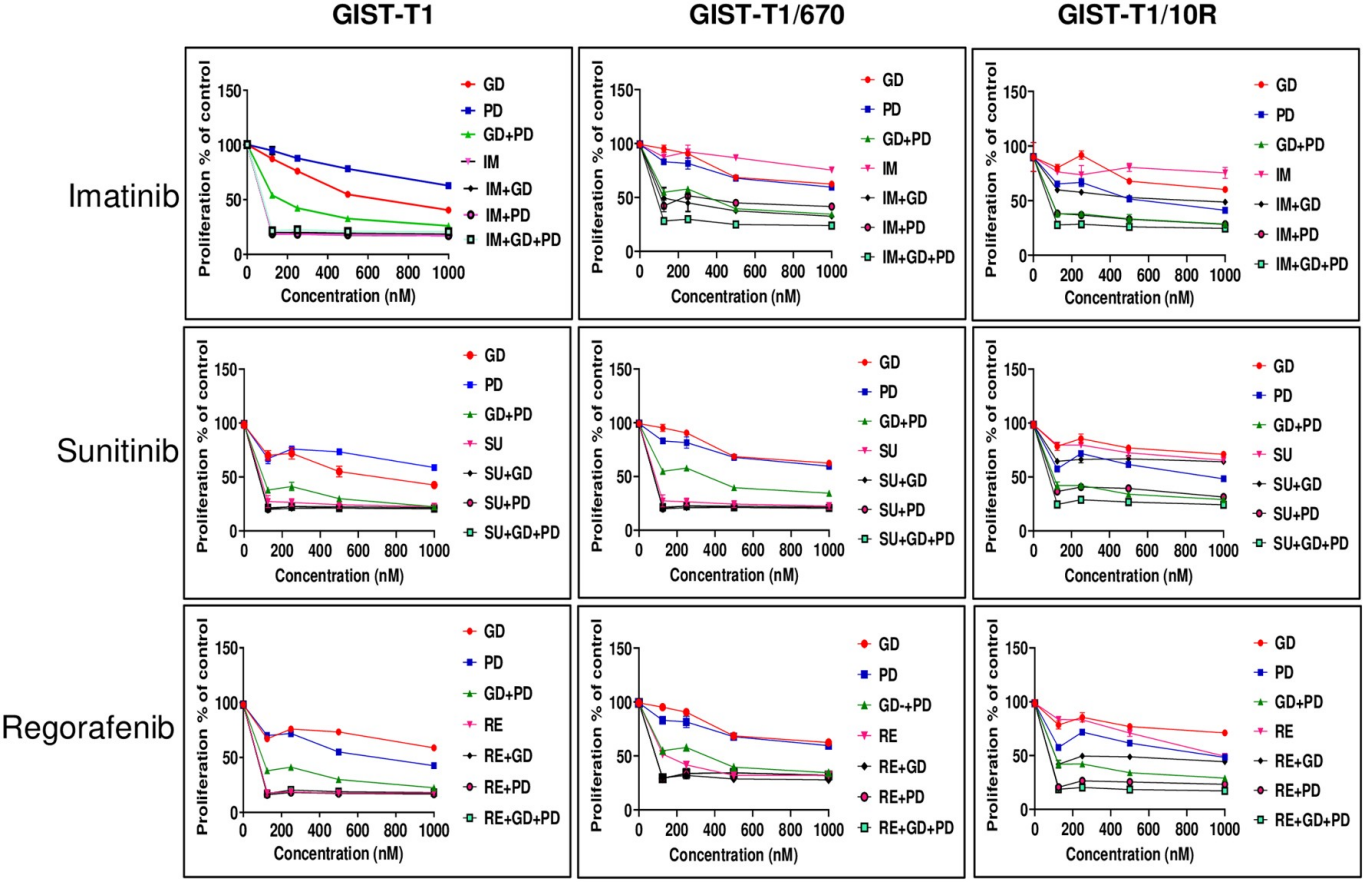
KIT and PI3K/MAPK Inhibitor combination significantly affects the proliferation of imatinib-resistant cell lines. Dose-response curves showing the proliferation of IM-sensitive (GIST-T1) and IM-resistant (GIST-T1/670, GIST-T1/10R) cell lines upon treatment with the indicated inhibitors. (IM: Imatinib; SU: Sunitinib, RE: Regorafenib, GD: GDC-0941, PD: PD 0325901). Cells were treated with varying concentrations of KIT inhibitors, GD and PD as indicated, PD was used at a fixed concentration of 500 nM in combination with varying doses of GD in GD+PD combination. In triple combination, GD and PD were used at a fixed dose of 500 nM with varying concentrations of KIT inhibitors. Proliferation was measured using the WST-1 reagent. Data is presented as percentage of control. Each point represents mean ± standard error, *n* = 3. The data is representative of three independent experiments.

As expected, IM significantly inhibited the proliferation of the GIST-T1, GIST 882 and GIST430 cell lines (Fig 2 and S2A Fig) and no further decrease was seen when IM was combined with GD, PD or both. GIST-T1/670, GIST-T1/10R and GIST430/654 cell lines remained refractory to IM treatment (Fig 2 and S2A Fig). A significant decrease in cell proliferation was observed when these cell lines were treated with PI3K/MAPK inhibitors in combination with IM. IM and GD combination led to a seventy-five percent (p<0.0001) decrease in cell proliferation in GIST-T1/670 and to a fifty percent decrease in GIST-T1/10R (p<0.01) compared to IM alone. The IM and PD combination was more effective than the IM and GD combination in the GIST-T1/10R cell line (p<0.0001 IM+GD vs. IM alone: p<0.0002 IM+GD vs IM+PD) (Fig 2). Although any dual combination with IM caused a decrease in proliferation (p<0.0001, IM+GD and IM+PD vs imatinib), the most effective combination was the KPM (KIT/PI3K/MAPK) inhibitor cocktail (IM+GD+PD) that reduced cell proliferation by greater than 80% in the GIST-T1/670 cell line (p<0.0001) (Fig 2, top row). In GIST-T1/10R, addition of GD to IM+PD did not show any added advantage, suggesting that this cell line is dependent on MAPK pathway. Similarly, GIST430/654 showed a significant decrease in cell proliferation when IM was combined with GD, PD or both (p<0.0001) (S2A Fig).

GIST-T1 is also sensitive to sunitinib (SU) and addition of GD or PD did not inhibit cell proliferation further. However, GIST882 and GIST430 showed a significant decrease with triple combination compared with either GD (p<0.0002 for GIST882; p<0.008 for GIST430), or PD (p<0.0001 for GIST882 an p<0.002 for GIST430) respectively (S2A Fig). GIST-T1/670 carries an additional T670I substitution and is also SU-sensitive. SU is used clinically to treat patients presenting with this secondary resistance mutation. As expected, SU treatment significantly decreased the proliferation of GIST-T1/670 cell line and addition of GD or PD did not cause any additional decrease (Fig 2). Proliferation of GIST-T1/10R was not significantly inhibited when treated with SU alone, but the addition of either GD or PD or a combination of both significantly inhibited growth (p<0.0001). GIST430/654 did not show a significant difference in proliferation with SU in combination with GD or PD but the triple combination showed better efficacy (p<0.04) (S2A Fig).

When the IM-resistant cell lines were treated with another FDA-approved KIT inhibitor, regorafenib (RE), either alone or in combination with GD or PD, similar results were obtained (Fig 2). Regorafenib significantly inhibited the proliferation of GIST-T1 and GIST 882 and addition of GD or PD or a combination thereof did not have any additional inhibitory effect. In GIST430, a combination of GD or PD significantly decreased proliferation (p<0.0001) but the triple combination was better (p<0.003 RE+GD vs. RE+GD+PD; p<0.001, RE+PD vs. RE+GD+PD at 200 nM of RE) (S2A Fig). RE alone was not sufficient to inhibit the proliferation of GIST-T1/10R but proliferation of GIST-T1/670 and GIST430/654 cell line was significantly inhibited and addition of either GD or PD inhibited the proliferation of all 3 cell lines (p<0.0001) (Fig 2 and S2A Fig). Again, RE+PD was more effective than RE+GD (p<0.0001), suggesting that GIST-T1/10R is dependent on MAPK pathway (Fig 2). Consistently, the KPM inhibitor cocktail caused a further decrease in cell proliferation (>80%) (p<0.004) (Fig 2). Therefore, a complete shutdown of KIT/PI3K and MAPK pathways elicited the most effective response.

In order to confirm that inhibition of MAPK pathway would be more effective in inhibiting the growth of GIST cell lines, we used another MEK inhibitor trametinib (TR) (a FDA approved drug), either alone or in combination with KIT inhibitors and GD. TR displayed properties similar to that of PD when administered in combination with other drugs except that it was better at inhibiting cell proliferation than PD when administered as a single agent. The KPM inhibitor cocktail with trametinib was much more effective than any single agent KIT inhibitor, indicating that synergistic effects occur when MEK inhibitors are coupled with KIT inhibitors (S2B Fig). Overall, our results suggest that drug resistance in GIST cell lines to KIT inhibitors could be overcome through combined KIT/MAPK or KIT/PI3K/MAPK pathway inhibition.

### MAPK and PI3K signaling remain active in GIST cell lines despite KIT inhibition

**Fig 3:**
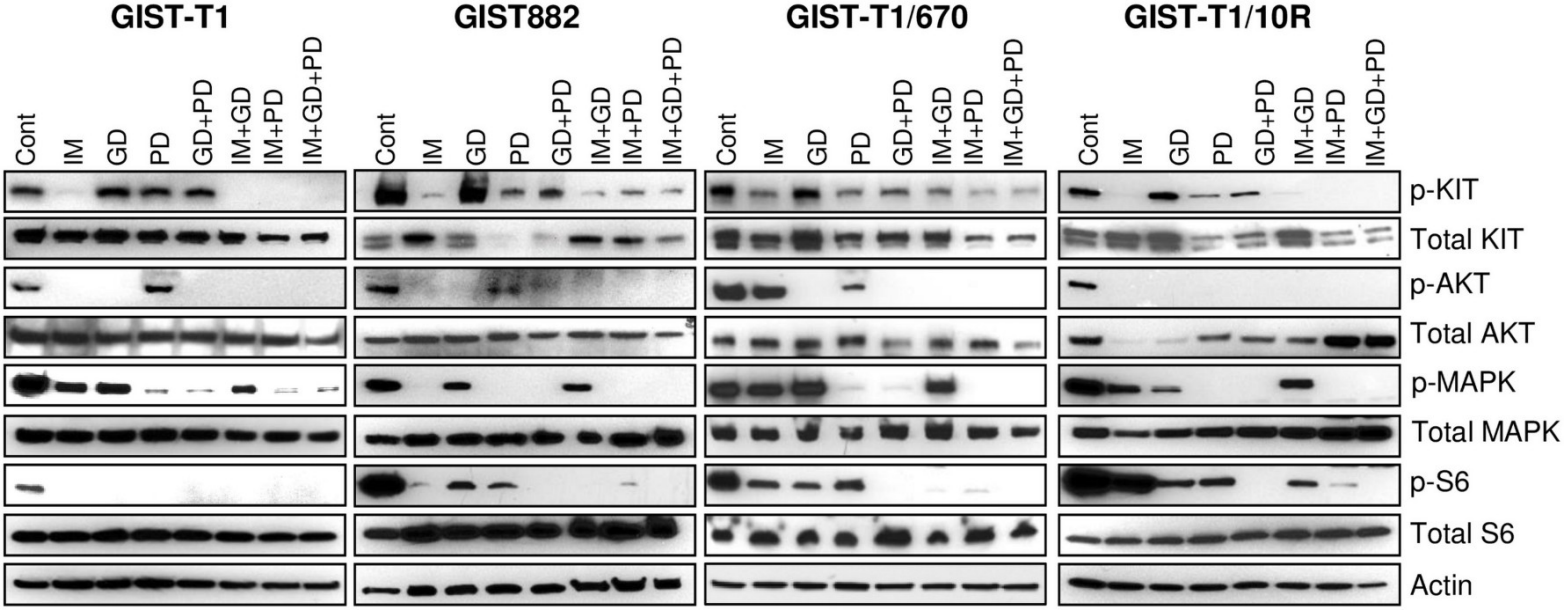
Active MAPK and PI3K signaling in GIST cell lines despite KIT inhibition. Lysates from GIST-T1, GIST882, GIST-T1/670 and GIST-T1/10R cells were analyzed using immunoblots to detect the total and phosphorylated levels of the indicated proteins. Cells were treated with the indicated inhibitors for 24 h–IM: imatinib (1 μM); GD: GDC-0941 (500 nM), PD: PD0325901 (500 nM). Data is representative of two independent experiments.

PI3K and MAPK signaling are downstream of KIT and here we probed the extent of phosphorylation inhibition in these signaling pathways upon KIT inhibition. IM-sensitive (GIST-T1, GIST882) and IM-resistant (GIST-T1/670, GIST-T1/10R) cell lines were treated with various inhibitors and inhibitor combinations. The levels of total and phosphorylated forms of ERK1/2 (MAPK pathway), AKT (PI3K pathway) and S6 (mTOR pathway, downstream of PI3K) were analyzed by western blots (Fig 3). The blots also served as a basis to evaluate the efficacy of the inhibitors used in this study. GD only inhibited PI3K/AKT pathway and PD was specific for the MAPK pathway. Both drugs in combination were not sufficient to completely inhibit KIT phosphorylation (Fig 3). Although IM significantly inhibited KIT, AKT and MAPK phosphorylation in GIST882, it only partially suppressed the downstream MAPK signaling in GIST-T1 (50% inhibition) and complete inhibition required the addition of the MEK inhibitor PD (Fig 3, left panel). These results suggest that active MAPK signaling might promote GIST-T1 survival in the presence of IM.

Only partial inhibition of KIT phosphorylation was observed in GIST-T1/670 upon IM treatment and this could explain why both PI3K and MAPK signaling appear to be fully functional—as determined by phosphorylation of AKT and ERK, respectively (Fig 3). Further, combinatorial treatment with at least two agents was required for complete inhibition of S6 phosphorylation. IM with PD treatment completely abrogated both MAPK and PI3K signaling whereas IM with GD combination only inhibited the PI3K pathway. PD treatment alone remarkably reduced the expression level of KIT, suggesting that there is a positive feedback between MAPK signaling and KIT expression (Fig 3 and S1C and S3 Figs).

Although IM completely inhibited phosphorylation of KIT in GIST-T1/10R, MAPK signaling was still active (Fig 3, right panel), suggesting KIT-independent activation of MAPK pathway. Phosphorylation of S6 also showed no significant inhibition after IM treatment. However, triple combination (KPM inhibitor cocktail) completely abrogated signaling through KIT and PI3K/MAPK pathways (Fig 3, right panel).

Sunitinib (SU) and regorafenib (RE) inhibited PI3K and MAPK signaling more efficiently than IM in GIST-T1 (S3A Fig). However, SU and RE as a single agent failed to completely inhibit MAPK signaling in GIST-T1/10R (S3A Fig). Complete inhibition of MAPK signaling required a dual combination with PD or the use of KPM inhibitor cocktail with either SU or RE as components.

Similar results were obtained when TR was used in combination treatment instead of PD (S3B Fig). Overall, our data indicate that KIT inhibitor treatment alone does not fully suppress MAPK signaling; residual PI3K signaling was also observed in the GIST-T1/10R cell lines, which might play a role in sustaining proliferation and survival in the presence of KIT inhibitors. Therefore, combination with PI3K or MAPK inhibitors are needed to completely shut down the signaling pathways.

### Combined inhibition of KIT, PI3K and MAPK signaling pathways induces apoptosis in GIST cells

**Fig 4:**
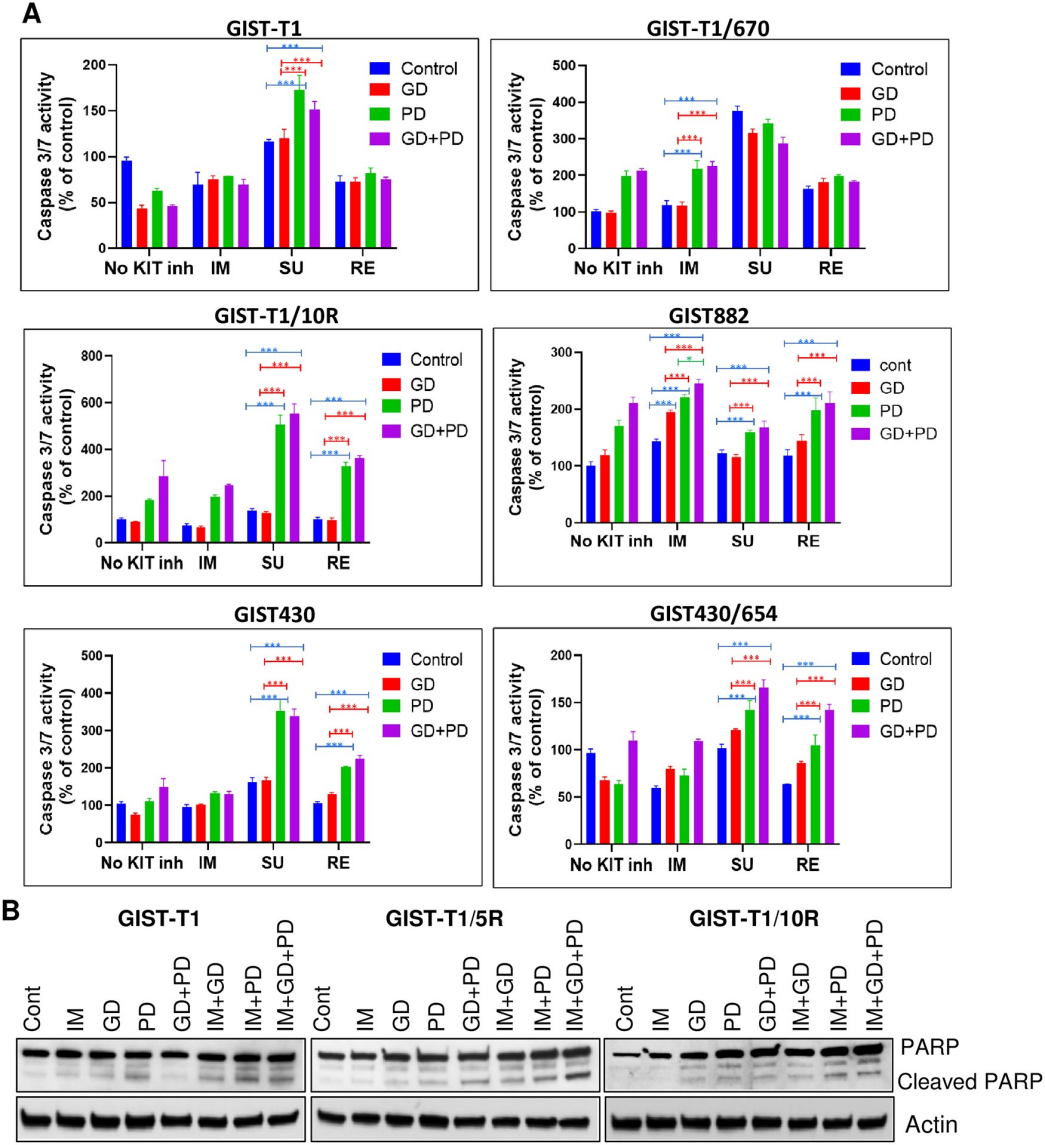
Combined Inhibition of KIT, PI3K and MAPK signaling pathways induces apoptosis in GIST cells. (A) Caspase 3/7 activity was measured to monitor apoptosis in GIST-T1, GIST-T1/670, GIST-T1/10R, GIST430 and GIST430/654 cells after treatment with various drug/drug combinations for 48 h, and in GIST882 cells after 24 h of treatment. The data is represented as percent increase over untreated cells; each column represents mean of triplicates. The data is representative of three independent experiments (Control: DMSO GD: GDC-0941, PD: PD 0325901). Each point represents mean ± standard error, *n* = 3. p values for various combinations were compared within each KIT inhibitor treatment group. (***p<0.0001); (**p<0.001); (*p<0.05). (B) Cleaved PARP levels in GIST-T1, GIST-T1/670 and GIST-T1/10R cells after treatment with indicated inhibitors for 24 h–IM: imatinib (1 μM) GD: GDC-0941 (500 nM), PD: PD 0325901 (500 nM).

Given the pronounced effect of the KPM inhibitor cocktail in reducing proliferation and inhibiting signaling, we investigated whether it is also effective in inducing apoptosis in imatinib-resistant GIST cells, particularly in GIST-T1 and GIST-T1/10R. Disease progression can be reduced or delayed if apoptosis is induced in drug-resistant GIST cells. Cells were treated with the indicated inhibitors or combinations thereof, and caspase activity was used as a measure of apoptosis. The data is presented as a percent increase in caspase activity over untreated cells (Fig 4A). Imatinib and regorafenib, either as single agents or in combinations did not induce apoptosis in GIST-T1 cells. Sunitinib alone or in combination with GD was not effective either. However, sunitinib in combination with PD led to an increase in apoptosis (p<0.0001 SU+GD/SU+PD vs. SU) and KPM combination did not have any additive effect (Fig 4A).

In imatinib-resistant GIST-T1/670 cells, imatinib or GD treatment alone did not induce apoptosis. However, PD enhanced apoptosis by about two-fold (p<0.0001). There was no further appreciable increase in apoptosis when PD was combined with GD or with KPM inhibitor cocktail containing imatinib suggesting that inhibition of MAPK pathway may be important in induction of apoptosis in this cell line (Fig 4A). In contrast to imatinib, sunitinib and regorafenib were able to induce apoptosis effectively, with sunitinib being the most effective–a three-fold increase in apoptosis levels were observed (P<0.0001) (Fig 4A). Since this cell line is sensitive to sunitinib, no further increase was seen with addition of GD, PD or both.

In GIST-T1/10R cell line, imatinib alone or in combination with GD did not show increase in caspase activity. Combination of imatinib with PD led to about a two-fold increase (p<0.0001) and triple combination increased it even further (~2.5-fold increase over control, but the latter increase was not statistically significant. Sunitinib alone did not induce apoptosis in GIST-T1/10R cell line and combined treatment of sunitinib with PD or KPM inhibitor cocktail (SU+GD+PD) caused a dramatic enhancement of apoptosis (p<0.0001) (Fig 4A). The effect was mostly due to PD in KPM combination as SU+GD+PD did not show a significant increase over sunitinib+PD combination. Similar to sunitinib, regorafenib was effective in inducing apoptosis in this cell line when combined with PD or when used as a component of the KPM inhibitor cocktail (p<0.0001) (Fig 4A). The combination of KIT inhibitors with trametinib also showed a significant increase in caspase activity (p<0.0001) and the KPM inhibitor cocktail was the most potent in inducing apoptosis in the GIST-T1/10R cell line when PD was replaced with trametinib (p<0.0001, SU+GD+PD vs. SU +PD; p<0.0001, RE+GD+PD vs. RE +PD) (S4B Fig).

A significant increase in caspase 3/7 activity was seen in GIST882 when treated with a combination of GD (p<0.0001) and PD (p<0.0001) compared to KIT inhibitor alone (Fig 4A). Combination of IM+GD+PD was even better than IM+GD (p<0.0001) or IM+PD (p<0.03). Similar to GIST882, an increase in apoptosis was also observed in GIST430 when sunitinib and regorafenib were used in combination with PD (P<0.0001) and addition of GD to these combinations did not have an additive effect on increasing caspase activity (Fig 4A). In GIST430/654 an imatinib resistant cell line, GD+PD was better than any double combination with KIT inhibitors and addition of imatinib did not further increase apoptosis. Sunitinib/regorafenib in combination with PD and GD+PD showed a significant increase in apoptosis in this cell line when compared to KIT inhibitors alone (p<0.0001) (Fig 4A). Similar to GIST-T1/670, sunitinib showed better efficacy in this cell line as V654A, the secondary resistance mutation in GIST430/654 is sensitive to sunitinib. Overall, our results showed that combination of KIT inhibitors with PD was better than GD and sunitinib/regorafenib in combination with PD performed better in imatinib resistant cell lines.

Apoptosis was also evaluated by PARP cleavage and GIST-T1 showed an increase in cleaved PARP with PD alone or in combination treatments with imatinib. GIST-T1/670 also had elevated levels of cleaved PARP with either GD+PD or imatinib in combination with GD or PD. Triple combination showed even higher levels of cleaved PARP. Similar to caspase activity, GIST-T1/10R cells showed an increase in cleaved PARP levels with IM+PD and IM+GD+PD combination (Fig 4B). Sunitinib/regorafenib as a single agent or in combination also led to increase in cleaved PARP levels in GIST-T1 (S4A Fig). Similarly, sunitinib and regorafenib alone induced cleavage of PARP which was further increased in combination with GD, PD or both in GIST-T1/670 cells, whereas sunitinib/regorafenib induced cleavage of PARP with PD or GD+PD in GIST-T1/10R cells. In summary, combination of PD and GD+PD was better in inducing cleaved PARP levels in all three cell lines.

### Effective reduction in colony outgrowth after treatment with the KPM inhibitor cocktail

**Fig 5:**
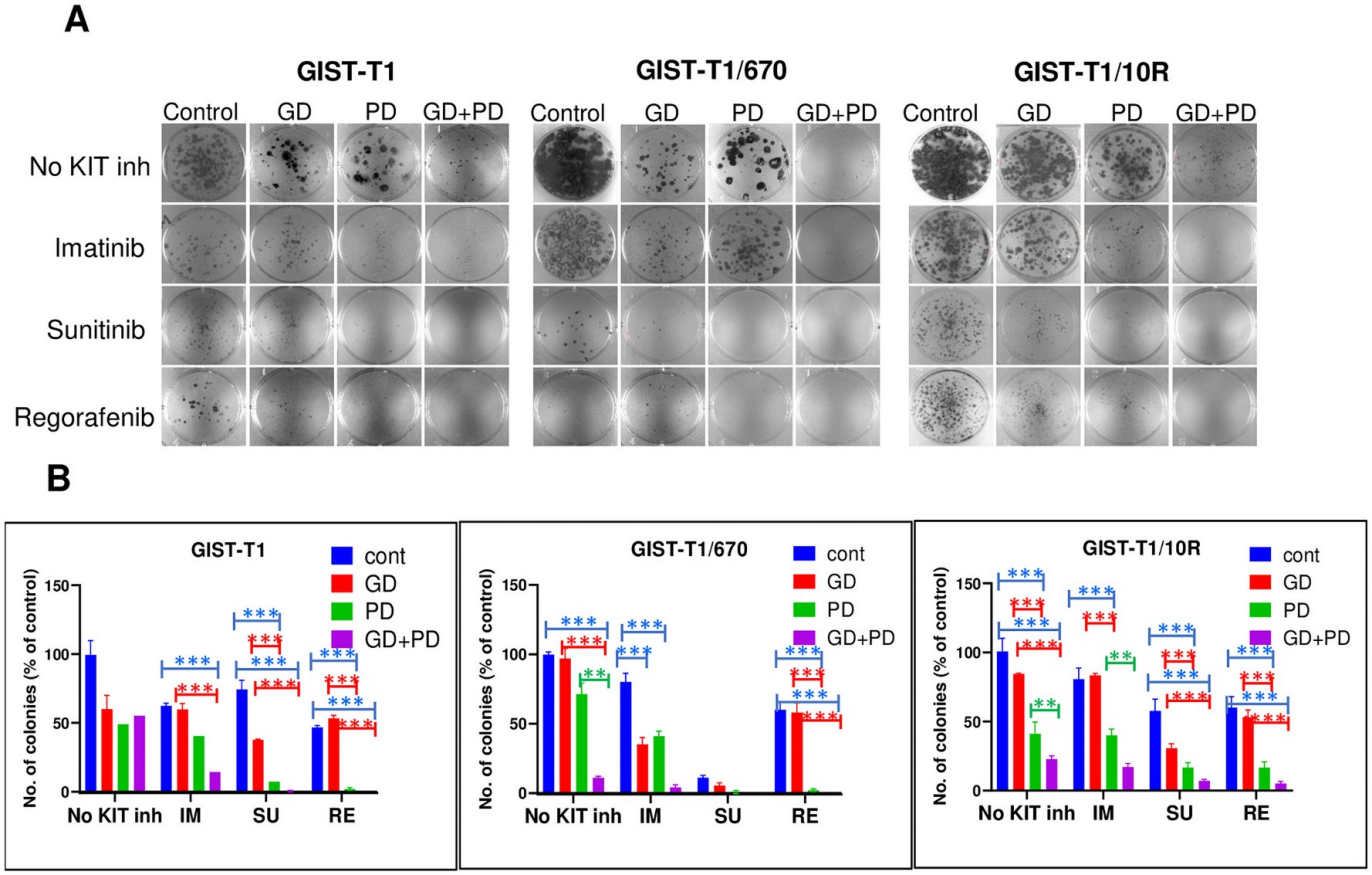
Effective reduction in colony growth after treatment with the KPM inhibitor cocktail. (A) Colony outgrowth assay to evaluate the long-term survival of GIST cell lines in the presence of indicated drugs. After 1-month duration of treatment, cells were allowed to grow in drug-free media for two weeks before staining with crystal violet. (B) Quantification of colony counts from the clonogenic assay, each column represents average of three wells. Each point represents mean ± standard error, *n* = 3. The data is representative of three independent experiments. p values for various combinations were compared within each KIT inhibitor treatment group. (***p<0.0001); (**p<0.005); (*p<0.01).

Caspase assay or PARP cleavage measures apoptosis in cells dying in culture at a certain time, while colony formation assay measures all modalities of cell death over a long period of time and is considered a gold standard to assess the effects of chemotherapy [33]. To study the cytotoxic effects of the various therapies, we used a modified clonogenic assay where the colony outgrowth was assessed after drug removal. Both imatinib–resistant and–sensitive GIST cells were treated with the indicated combination of drugs for 1 month. To estimate cell survival after treatment, the drugs were washed out and cells were allowed to grow drug-free for two weeks and the colonies were stained with crystal violet to facilitate visualization. In the presence of imatinib, GIST-T1 cells entered a quiescent state. Imatinib was not cytocidal as the cells proliferated to form colonies when the drug was removed (Fig 5). The combination of imatinib with GD or PD, significantly reduced the number of colonies (p<0.001), although it was not fully cytocidal either, as roughly half the number of colonies still grew out after drug removal. Effective suppression of colony growth required the KPM inhibitor cocktail (IM+GD+PD), which reduced colony outgrowth to <15% (p<0.001) (Fig 5A and 5B). Sunitinib alone or in combination with GD did not completely suppress colony outgrowth, whereas sunitinib with PD combination was effective (p<0.004) and it reduced colony outgrowth to 7%. A similar trend was seen with regorafenib where effective colony growth suppression required combination with PD (p<0.004), or GD and PD (KPM inhibitor cocktail).

Remarkably, KIT inhibitors as single agents failed to effectively suppress colony outgrowth in GIST-T1/670 and GIST-T1/10R cell lines although sunitinib as a single agent was effective against GIST-T1/670 (Fig 5A and 5B). GD and PD dual combination was effective (p<0.001) but KIT was still active when this combination was used (Fig 3). KIT inhibitors in combination with PD also effectively suppressed colony growth (p<0.0001). Again, the KPM inhibitor cocktail with either imatinib, sunitinib or regorafenib caused the most pronounced reduction of colony outgrowth in both of the resistant cell lines (Fig 5A and 5B). We also investigated the effect of trametinib (TR) in clonogenic assays (S5 Fig). TR was also as potent as PD and, similar to PD, inclusion of TR as a component of the KPM inhibitor cocktail caused a dramatic reduction in colony outgrowth (p<0.0001) (S5 Fig). This cocktail is effective in both imatinib-sensitive and -resistant cell lines.

## Discussion

There are a variety of primary gain-of-function mutations in the receptor tyrosine kinases (RTKs) KIT and PDGFRA that are the prime oncogenic drivers of GIST [1, 2]. By inhibiting these RTKs, imatinib dramatically improved the survival of patients with metastatic GIST [34]. Complete responses to imatinib treatment are rare and continued imatinib treatment causes drug resistance 18–24 months after the initiation of therapy [30, 35]. We have shown previously that one of the mechanisms of resistance is that cells enter into a quiescence phase and can remain in this state for a long time [9]. Incomplete inhibition of downstream signaling pathways after treatment with IM can contribute to survival of drug-treated cells. Resistance arises most often through secondary intra-allelic *KIT* mutations, which abrogate binding of the drug to the kinase domain and patients no longer respond to imatinib. Sunitinib and regorafenib target only a subset of common secondary resistance mutations and patients eventually develop resistance to these inhibitors as well, leading to only a modest improvement in progression-free survival [12, 13]. Currently, two recently approved KIT inhibitors avapritinib (for PDGFRA-D842V) and ripretinib as 4^th^ line therapy for GIST, hold promise to treat some of the secondary resistance mutations of KIT/PDGFRA in metastatic GIST [36]. However, none of these approved inhibitors target all the secondary KIT mutations observed in clinic with equal potency. Besides secondary resistance mutations in KIT or PDGFRA, concomitant mutations in other signaling proteins, such as EGFR, PI3K, RAS, PTEN, NF1, TSC2, SDH, PP2A, FGFR1, FGFR3, and FGF4 have been reported in GIST patients and are associated with poor prognosis [37]. Based on these reports, we reasoned that simultaneous targeting of deregulated downstream pathways along with KIT inhibitors should effectively prevent the progression of drug-resistant GIST.

To identify the pathways that promote drug resistance, we used 40 FDA-approved or investigational drugs that target various cell survival pathways. Sorafenib stood out as it induced apoptosis in all 4 GIST cell lines tested and the induction of apoptosis was much higher in the two imatinib resistant cell lines. Since sorafenib is a dual inhibitor of KIT and RAF, it was tempting to speculate that MAPK pathway inhibitors might induce apoptosis in GIST cell lines. It was intriguing to see that RAF inhibition induced apoptosis in GIST-T1/10R which is resistant to all KIT inhibitors with no secondary resistant mutations in KIT. Ras signaling pathway evaluation showed that GIST-T1/10R has activated Ras and was even further activated in the presence of imatinib or sorafenib. Since, sorafenib inhibits signaling downstream of Ras, MEK inhibition was still observed. To confirm that MEKi is indeed responsible for induced apoptosis, PD was used in combination with KIT inhibitors as it was already on our drug screen and had shown efficacy as a single agent. The PI3K inhibitor GD was also included in the study as the RAS pathway can also activate AKT.

Three FDA-approved KIT inhibitors; imatinib, sunitinib and regorafenib were used to identify which KIT inhibitor would show better efficacy in combination with MEKi in our studies. Although sorafenib showed cytotoxicity in our initial screen, it was not used in the follow up studies. Sorafenib and regorafenib are nearly identical compounds (with a difference of a fluorine atom) and show similar activity profiles in enzymatic assays [17]. In clinical trials with sorafenib, overall progression free survival in GIST patients is <6mo and overall response rate < 10%, which are comparable to regorafenib [38, 39]. Clinical development of sorafenib for GIST was stopped by Bayer Pharmaceuticals in favor of regorafenib. However, regorafenib did not seem to be as potent as sorafenib as a single agent based on caspase assay in this study. It could be due to differences in pharmaceutical properties reported in preclinical models of colorectal [40] and hepatocellular carcinoma [41] and some off-target effects in cell culture conditions. Further studies are needed to understand the difference between sorafenib and regorafenib, observed in this study.

Our results show that KIT inhibitors as a single agent, with the exception of sunitinib, in GIST-T1/670, were not effective at inducing apoptosis in imatinib-sensitive and -resistant cell lines. Imatinib alone was unable to completely turn-off MAPK signaling in GIST-T1, and PI3K/MAPK signaling in resistant cell lines, suggesting that residual signaling can facilitate survival of GIST cells in the presence of KIT inhibitors. A combination with GD had little effect on apoptosis and only showed a modest response in colony outgrowth assays, suggesting that combination of KIT inhibitors with GD is also cytostatic. Consistent with our results, Floris et al [42] has shown that GD treatment results in a significant reduction in proliferative activity leading to either tumor growth delay or tumor burden stabilization, but no apoptosis was observed in xenograft models. They suggested that the survival of GIST cells does not depend on AKT activity alone even though AKT is constitutively activated in IM-sensitive GIST cell lines.

KIT inhibitors in combination with PD were more effective at inhibiting cell proliferation and inducing apoptosis in all three cell lines tested. Out of 3 KIT inhibitors, the combination of sunitinib or regorafenib with PD was more effective than the combination of imatinib+PD, suggesting that MAPK signaling plays an important role in GIST cell survival and a combination of MEK inhibitors with 2^nd^ and 3^rd^ line KIT inhibitors might be a more effective strategy to overcome resistance. Synergistic responses have also been documented in other reports where imatinib or PLX3397 (another KIT inhibitor) in combination with a MAPK inhibitor binimetinib, showed a prolonged response in comparison with a single KIT inhibitor [43]. MAPK pathway also appears to reinforce KIT signaling through positive feedback in imatinib resistant cell lines and inhibition of these downstream signaling components improves drug sensitivity. For instance, we consistently observed that MAPK pathway inhibition through PD also reduced the expression levels of the KIT oncoprotein, particularly in the imatinib-resistant cell lines (Fig 3), possibly due to inhibition of transcription factor ETV1, which maintains KIT mRNA levels [43]. Targeting of GIST with PI3K and MAPK pathway inhibitors (KPM inhibitor cocktail) was even better than dual combination with KIT inhibitor and PD, leading to pronounced reduction or complete eradication of colony outgrowth in GIST-T1/670 and GIST-T1/10R cell lines. Imatinib failed to inhibit phosphorylation of AKT, p-ERK and pS6 in GIST-T1/10R, demonstrating its dependence on PI3K/MAPK signaling. Inhibition of both PI3K and MAPK with GD and PD in this cell line led to induction of apoptosis and inhibition of outgrowth of colonies. The GD and PD combination was not as effective as the triple combination, further suggesting that targeting KIT is still needed in this cell line. Interestingly, these two pathways are also activated in patients with either *KIT/PDGFRA/SDH* wild-type GIST or KIT mutants that progress on KIT inhibitors without developing secondary mutations [44]. These findings indicate that co-activation of the MAPK and PI3K pathways in GIST fosters KIT-independence and contributes to KIT inhibitor resistance. Here, we demonstrate that combined inhibition of KIT/PI3K/MAPK signaling using the KPM inhibitor cocktail is the most effective means to combat drug resistance in GIST.

Overall, our data shows that a KIT and MEK inhibitor combination has distinct advantages over treatments with KIT inhibitors alone in killing GIST cells and triple combination with addition of a PI3K inhibitor is even better. Furthermore, sunitinib/regorafenib showed superior efficacy compared with imatinib in combination with MAPK signaling inhibitors in inducing cytocidal responses in both imatinib -sensitive and -resistant GIST cell lines. Effective targeting of metastatic GIST with a plethora of primary and secondary mutations is an unmet clinical need. KIT/PDGFRA inhibitors, are widely used as single agents for treating metastatic GIST. However, emergence of secondary drug resistance is an ongoing and unresolved problem with these inhibitors. To address drug resistance, a growing list of alternate targeting strategies using CDK, HSP90, IGF1R, immune checkpoint, PI3K, MEK, and MET inhibitors either alone or in dual combination with RTK inhibitors are being investigated in pre-clinical and clinical trials [45]. Here we demonstrate that the KPM inhibitor combination has distinct advantages over treatment with KIT inhibitors alone, as it has the potential to overcome the compensatory resistance mechanisms that arise in GIST. Dual inhibition of PI3K and MAPK pathway has shown benefit in a genetically engineered mouse model of imatinib-resistant GIST equivalent to ex 11 with T670I mutation of human KIT protein. However, other gate keeper imatinib-resistant KIT mutations in ex 13 V654A and KIT independent resistance mechanisms have not been explored [46]. Triple combination therapy using KIT/PI3K/MAPK inhibitors or PI3K/MAPK inhibitors might be particularly useful for heavily treated patients with primary KIT mutations but lacking secondary KIT mutations and also in patients with KIT exon 11 mutations, who do not respond to imatinib treatment, as these cohorts have activated MAPK/PI3K signaling [44, 46, 47].

MEK inhibitors have shown some toxicity in the clinic [48, 49], and it is likely that triple combination will be even more toxic. In vivo studies have shown that KIT inhibitors with MEK inhibitor combination [43] and with PI3K and MEKi [46] combination can be tolerated. Bosbach et al [46] has also shown that Alpelisib in combination with binimetinib was better tolerated than voxtalisib plus PD or pilaralisib plus PD. However, GD has not been tested in combination with PD, trametinib or binimetinib. If the triple combination shows toxicity, our study suggests that combination with MEK inhibitor can still provide significant benefit in clinic. Further studies are needed to test this combination in *in vivo*. Should this combination display tolerable toxicity profiles it could also be considered for 1^st^/2^nd^ line therapy for metastatic GIST with the goal of eradicating the tumor at the beginning of treatment, and minimize or prevent subsequent emergence of drug-resistant mutations. The combination would also benefit the patients who progress on KIT inhibitors without developing secondary resistant mutations.

## Supporting information

S1 Fig Sensitivity of various GIST cell lines to KIT inhibitors. (A) Dose-response curves showing the proliferation of IM-sensitive (GIST-T1 and GIST882) and IM-resistant (GIST-T1/670, GIST-T1/10R) cell lines upon treatment with the indicated inhibitors. (IM: Imatinib; SU: Sunitinib, RE: Regorafenib). Cells were treated with varying concentrations of KIT inhibitors as indicated and proliferation was measured using the WST-1 reagent. Data is presented as percentage of control, each point represents mean ± standard error, *n* = 3. (B) GD and PD combination is synergistic in GIST-T1 cell line. Surface map of Loewe’s synergy of GD and PD combination at indicated concentrations. GIST-T1 was treated with either inhibitor alone or in combination and proliferation was measured after 72 h of treatment. Values represent average of three replicates (Left panel). Matrix of Loewe synergy and antagonism with GD and PD combination in GIST-T1 cell line. (C) GIST882 cells were treated with the indicated inhibitor concentrations for 24 h and cell lysates were run on SDS-PAGE followed by immunoblotting to detect the indicated proteins. Data is representative of two independent experiments. (D) Average colony counts from colony outgrowth assay in GIST-T1 cell line treated with imatinib at the indicated concentrations. The colony numbers were normalized to the vehicle controls (Cont) and each column represents the mean of triplicates.
(TIF)

S2 Fig KIT and PI3K/MAPK inhibitor combination significantly affects the proliferation of GIST cell lines. (A) Dose-response curves showing the proliferation of IM-sensitive (GIST882 and GIST 430) and IM-resistant (GIST430/654) cell lines upon treatment with the indicated inhibitors for 72 h (IM: Imatinib; SU: Sunitinib, RE: Regorafenib, GD: GDC-0941, PD: PD 0325901). Cells were treated with varying concentrations of KIT inhibitors as indicated, PD was used at a fixed concentration of 500 nM in combination with varying doses of GD in GD+PD combination. GD and PD were used at a fixed dose of 500 nM for triple combination with varying concentrations of KIT inhibitors. Data is presented as percentage of control, each point represents mean ± standard error, *n* = 3. The data is representative of three different experiments. (B) Monitoring cell proliferation with a cocktail containing trametinib. Dose-response curves estimating cell proliferation after treatment of GIST-T1, GIST-T1/670 GIST-T1/10R, and GIST882 cell lines with varying concentrations of indicated KIT inhibitors for 72 h. TR was used at a fixed concentration of 500 nM in combination with varying doses of GD or GD+TR combination. For triple combination, PI3K inhibitor (GD) and trametinib (TR) was used at 500 nM concentration with varying concentrations of KIT inhibitors. Proliferation was measured using the WST-1 reagent. Data is presented as mean ± standard deviation, n = 3. Data is representative of three independent experiments.
(TIF)

S3 Fig MAPK and PI3K signaling is still active in GIST cell lines despite KIT inhibitor treatment. (A) Lysates from GIST-T1, GIST882, GIST-T1/670 and GIST-T1/10R cells were analyzed using immunoblots to detect the total and phosphorylated levels of the indicated proteins. The cells were treated with the indicated inhibitors for 24 h–GD: GDC-0941 (500 nM), PD: PD 0325901 (500 nM) Sunitinib (1 μM) (Upper panel) and Regorafenib (1 μM) (lower panel); data is representative of two independent experiments. (B) GIST-T1/10R cells were treated with the indicated inhibitors or combinations thereof for 24 h GD: GDC-0941 (500 nM), PD: TR: Trametinib (500 nM) Imatinib ((1 μM) Sunitinib (1 μM) and Regorafenib (1 μM); and cell lysates were run on SDS-PAGE followed by immunoblotting to detect the indicated proteins. Data is representative of two independent experiments.
(TIF)

S4 Fig Trametinib in combination with KIT and PI3K inhibitors induces apoptosis in GIST cell lines. (A) Lysates from GIST-T1, GIST-T1/670 and GIST-T1/10R cells were analyzed using immunoblots to detect the total and cleaved PARP levels. The cells were treated with the indicated inhibitors for 24 h–SU: sunitinib (1 μM); RE: regorafenib (1 μM); GD: GDC-0941 (500 nM), PD: PD 0325901 (500 nM) or a combination at these concentrations. (B) GIST-T1/10R cells were treated with either KIT inhibitors (1 μM) or GD (500 nM) or TR (500 nM) and combinations thereof as indicated for 48 h before measuring the caspase 3/7 activity. Data is presented as percentage of control and each column represents mean of triplicates. Each point represents mean ± standard error, *n* = 3. The data is representative of three different experiments. p values for various combinations were compared within each KIT inhibitor treatment group. (***p<0.0001).
(TIF)

S5 Fig Drug combinations with trametinib decrease the colony-forming ability of GIST cell lines. (A) Colony outgrowth (clonogenic) assay to assess the treatment response of imatinib-sensitive (GIST-T1) and -resistant (GIST-T1/670, GIST-T1/10R) cell lines to trametinib (TR) and the indicated drug combinations. After drug treatment, the cells were allowed to form colonies in drug-free media for two weeks before staining with crystal violet. (B) Quantification of colony numbers from the clonogenic assay, the colony numbers were normalized to the vehicle controls (Cont) and each column represents mean of triplicates. The experiment was repeated three times and representative data is shown. Each point represents mean ± standard error, *n* = 3. The data is representative of three different experiments. p values for various combinations were compared within each KIT inhibitor treatment group. (***p<0.0001).
(TIF)

S1 Raw images (PDF)
