# Supplementary figures and images for: Inhibition of PI3K and MAPK pathways along with KIT inhibitors as a strategy to overcome drug resistance in gastrointestinal stromal tumors
Source: PLoS One. 2021 Jul 29;16(7):e0252689. doi: 10.1371/journal.pone.0252689 (PMC8320897; doi:10.1371/journal.pone.0252689)

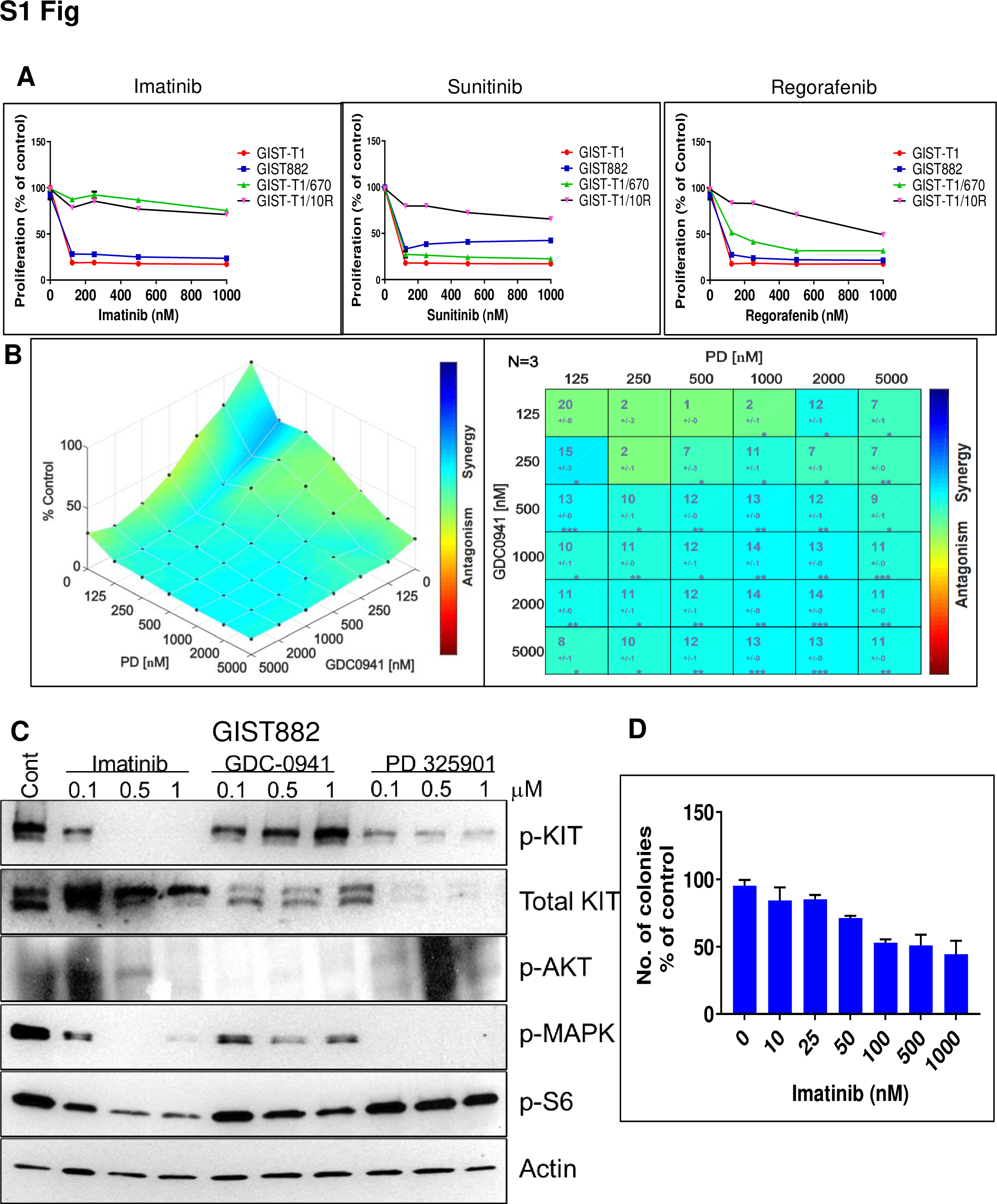

Supplement: S1 Fig — (A) Dose-response curves showing the proliferation of IM-sensitive (GIST-T1 and GIST882) and IM-resistant (GIST-T1/670, GIST-T1/10R) cell lines upon treatment with the indicated inhibitors. (IM: Imatinib; SU: Sunitinib, RE: Regorafenib). Cells were treated with varying concentrations of KIT inhibitors as indicated and proliferation was measured using the WST-1 reagent. Data is presented as percentage of control, each point represents mean ± standard error, n = 3. (B) GD and PD combination is synergistic in GIST-T1 cell line. Surface map of Loewe’s synergy of GD and PD combination at indicated concentrations. GIST-T1 was treated with either inhibitor alone or in combination and proliferation was measured after 72 h of treatment. Values represent average of three replicates (Left panel). Matrix of Loewe synergy and antagonism with GD and PD combination in GIST-T1 cell line. (C) GIST882 cells were treated with the indicated inhibitor concentrations for 24 h and cell lysates were run on SDS-PAGE followed by immunoblotting to detect the indicated proteins. Data is representative of two independent experiments. (D) Average colony counts from colony outgrowth assay in GIST-T1 cell line treated with imatinib at the indicated concentrations. The colony numbers were normalized to the vehicle controls (Cont) and each column represents the mean of triplicates. (TIF) [file pone.0252689.s001.tif]

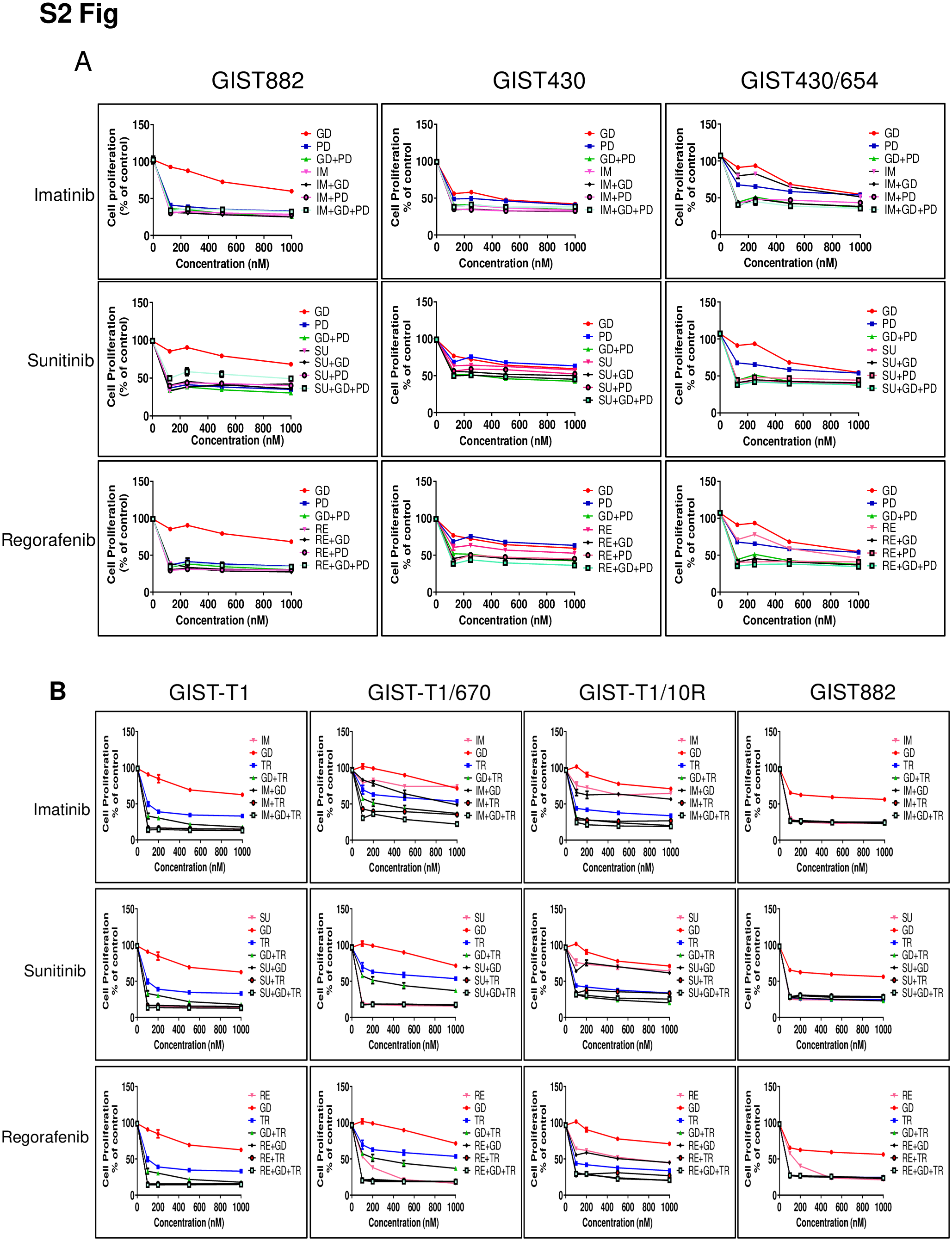

Supplement: S2 Fig — (A) Dose-response curves showing the proliferation of IM-sensitive (GIST882 and GIST 430) and IM-resistant (GIST430/654) cell lines upon treatment with the indicated inhibitors for 72 h (IM: Imatinib; SU: Sunitinib, RE: Regorafenib, GD: GDC-0941, PD: PD 0325901). Cells were treated with varying concentrations of KIT inhibitors as indicated, PD was used at a fixed concentration of 500 nM in combination with varying doses of GD in GD+PD combination. GD and PD were used at a fixed dose of 500 nM for triple combination with varying concentrations of KIT inhibitors. Data is presented as percentage of control, each point represents mean ± standard error, n = 3. The data is representative of three different experiments. (B) Monitoring cell proliferation with a cocktail containing trametinib. Dose-response curves estimating cell proliferation after treatment of GIST-T1, GIST-T1/670 GIST-T1/10R, and GIST882 cell lines with varying concentrations of indicated KIT inhibitors for 72 h. TR was used at a fixed concentration of 500 nM in combination with varying doses of GD or GD+TR combination. For triple combination, PI3K inhibitor (GD) and trametinib (TR) was used at 500 nM concentration with varying concentrations of KIT inhibitors. Proliferation was measured using the WST-1 reagent. Data is presented as mean ± standard deviation, n = 3. Data is representative of three independent experiments. (TIF) [file pone.0252689.s002.tif]

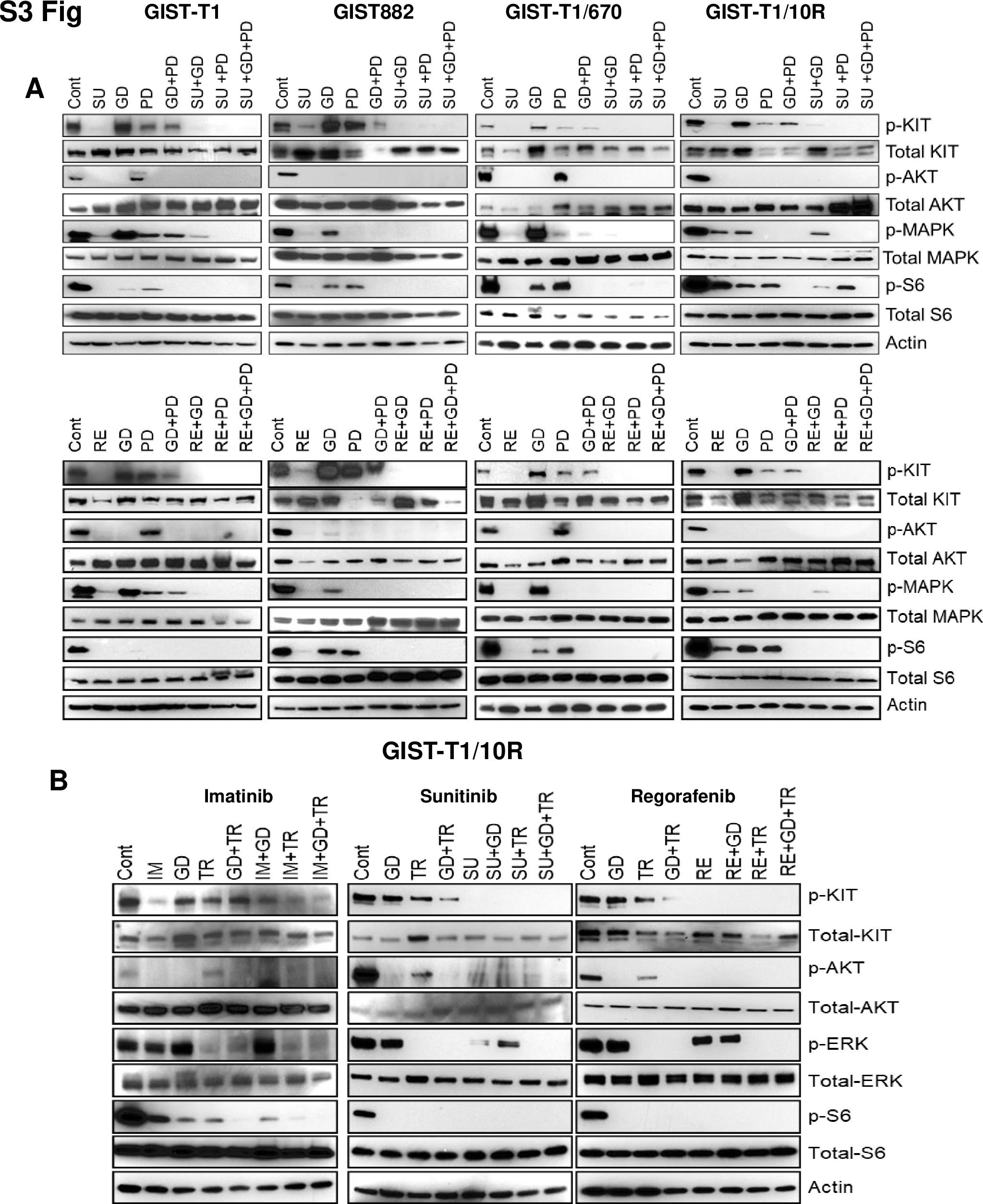

Supplement: S3 Fig — (A) Lysates from GIST-T1, GIST882, GIST-T1/670 and GIST-T1/10R cells were analyzed using immunoblots to detect the total and phosphorylated levels of the indicated proteins. The cells were treated with the indicated inhibitors for 24 h–GD: GDC-0941 (500 nM), PD: PD 0325901 (500 nM) Sunitinib (1 μM) (Upper panel) and Regorafenib (1 μM) (lower panel); data is representative of two independent experiments. (B) GIST-T1/10R cells were treated with the indicated inhibitors or combinations thereof for 24 h GD: GDC-0941 (500 nM), PD: TR: Trametinib (500 nM) Imatinib ((1 μM) Sunitinib (1 μM) and Regorafenib (1 μM); and cell lysates were run on SDS-PAGE followed by immunoblotting to detect the indicated proteins. Data is representative of two independent experiments. (TIF) [file pone.0252689.s003.tif]

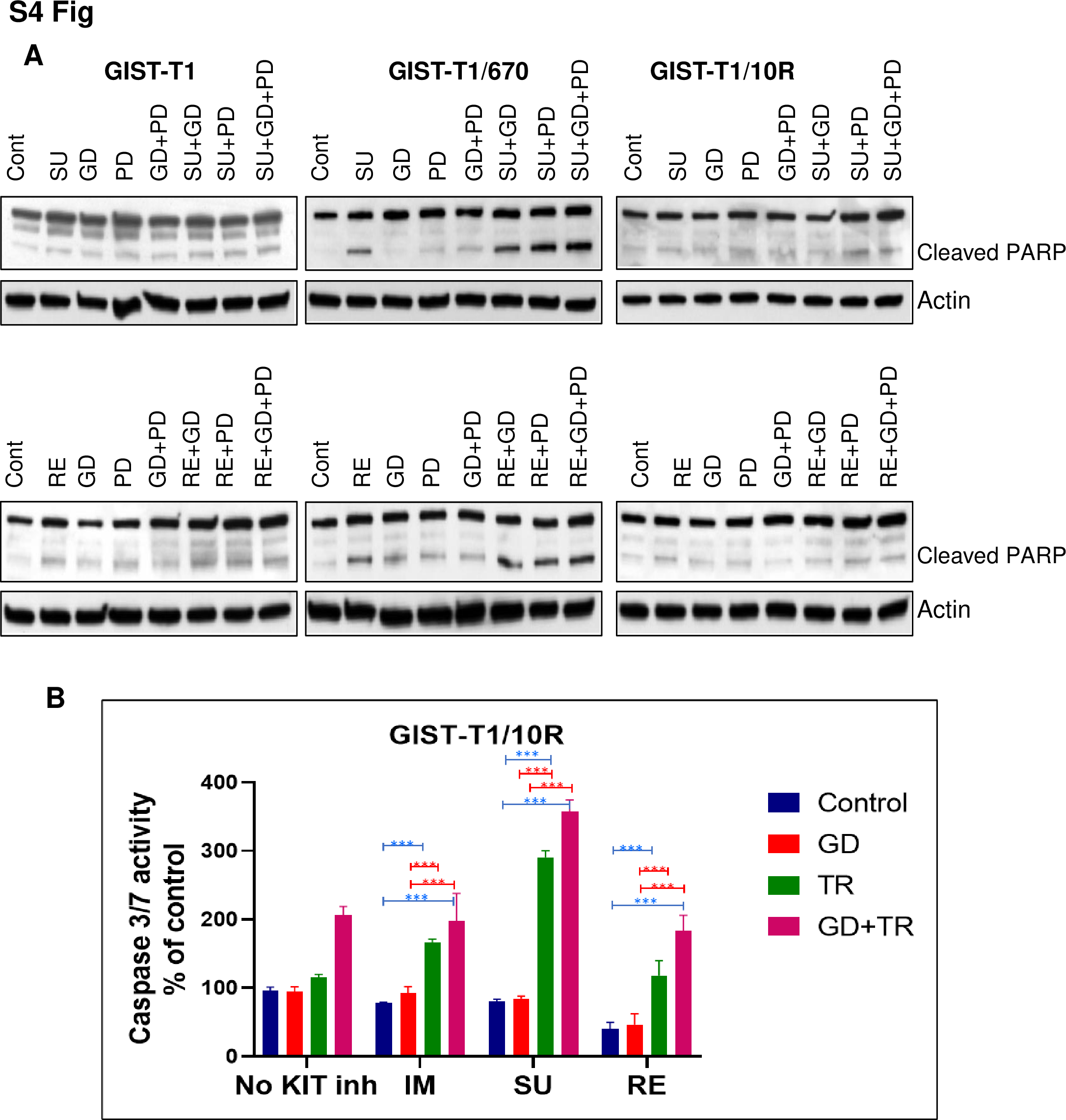

Supplement: S4 Fig — (A) Lysates from GIST-T1, GIST-T1/670 and GIST-T1/10R cells were analyzed using immunoblots to detect the total and cleaved PARP levels. The cells were treated with the indicated inhibitors for 24 h–SU: sunitinib (1 μM); RE: regorafenib (1 μM); GD: GDC-0941 (500 nM), PD: PD 0325901 (500 nM) or a combination at these concentrations. (B) GIST-T1/10R cells were treated with either KIT inhibitors (1 μM) or GD (500 nM) or TR (500 nM) and combinations thereof as indicated for 48 h before measuring the caspase 3/7 activity. Data is presented as percentage of control and each column represents mean of triplicates. Each point represents mean ± standard error, n = 3. The data is representative of three different experiments. p values for various combinations were compared within each KIT inhibitor treatment group. (***p<0.0001). (TIF) [file pone.0252689.s004.tif]

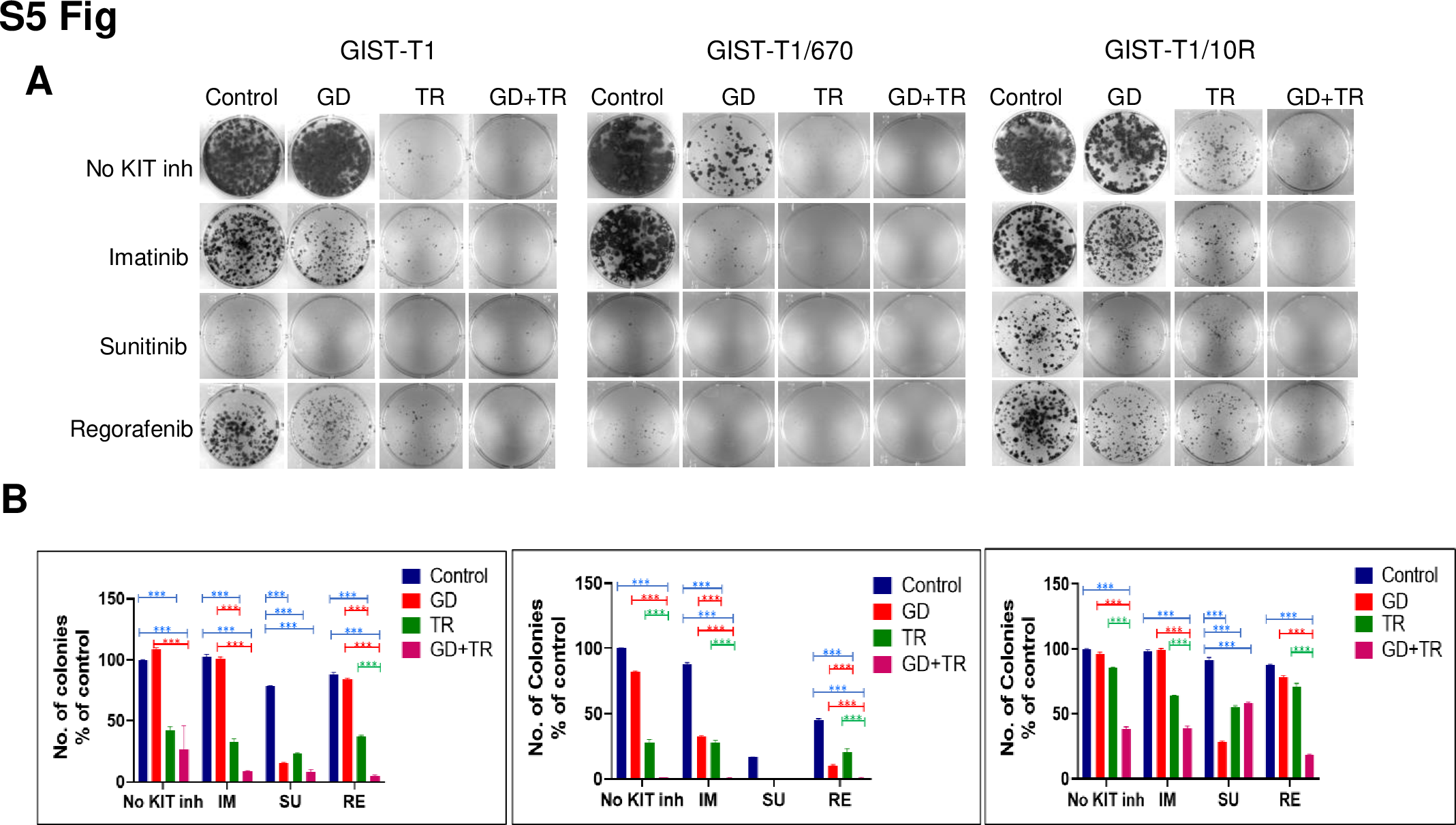

Supplement: S5 Fig — (A) Colony outgrowth (clonogenic) assay to assess the treatment response of imatinib-sensitive (GIST-T1) and -resistant (GIST-T1/670, GIST-T1/10R) cell lines to trametinib (TR) and the indicated drug combinations. After drug treatment, the cells were allowed to form colonies in drug-free media for two weeks before staining with crystal violet. (B) Quantification of colony numbers from the clonogenic assay, the colony numbers were normalized to the vehicle controls (Cont) and each column represents mean of triplicates. The experiment was repeated three times and representative data is shown. Each point represents mean ± standard error, n = 3. The data is representative of three different experiments. p values for various combinations were compared within each KIT inhibitor treatment group. (***p<0.0001). (TIF) [file pone.0252689.s005.tif]
