# Supplementary material for: Inhibition of PI3K and MAPK pathways along with KIT inhibitors as a strategy to overcome drug resistance in gastrointestinal stromal tumors
Source: PLoS One. 2021 Jul 29;16(7):e0252689. doi: 10.1371/journal.pone.0252689 (PMC8320897; doi:10.1371/journal.pone.0252689)

All images were scanned using either Storm Imager or Biorad chemidoc imaging system

Fig. 1B

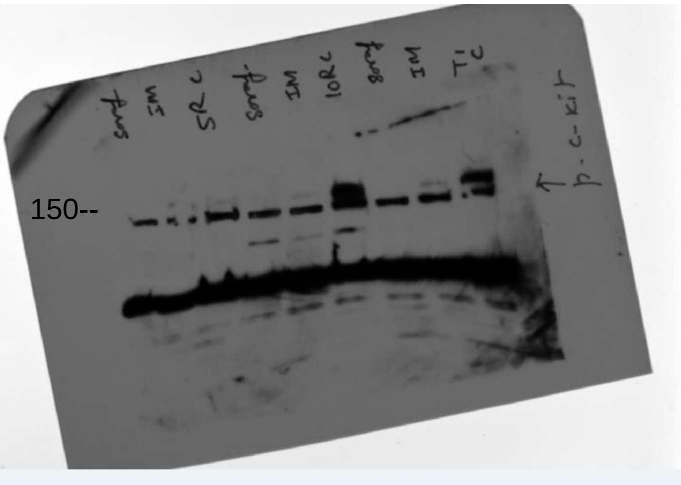

p-KIT

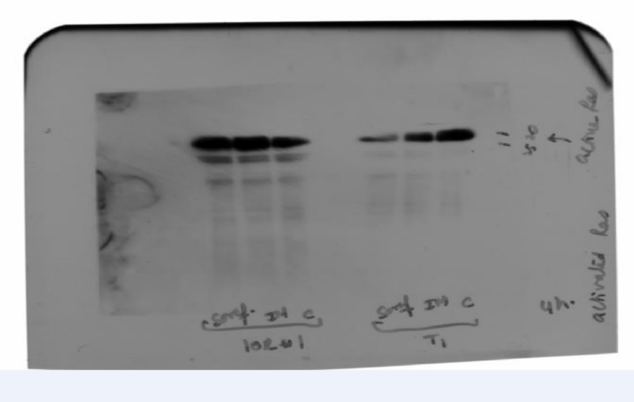

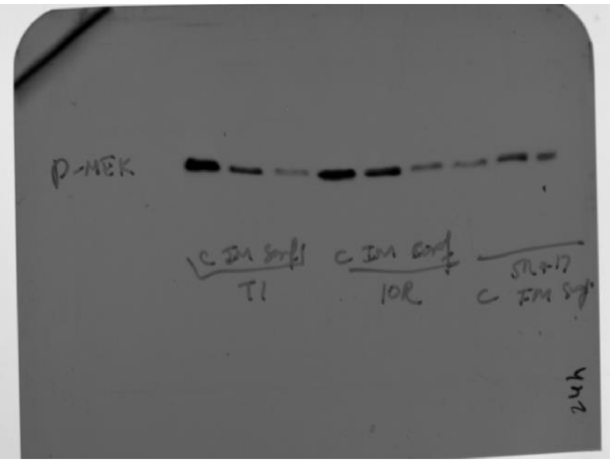

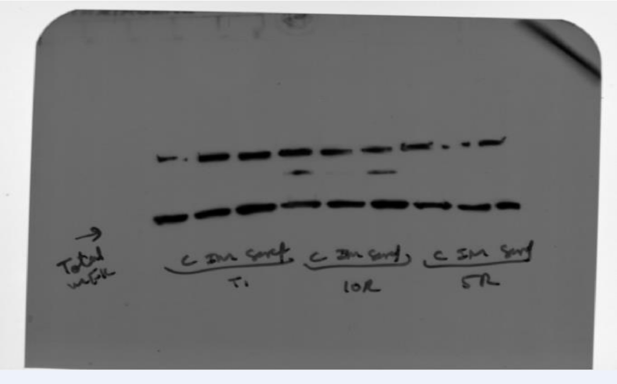

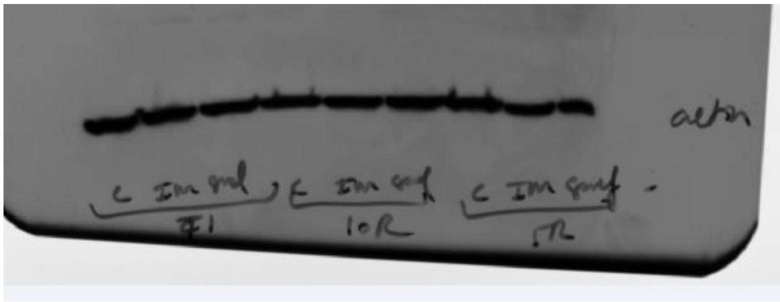

Fig. 1C

GIST-T1—(Left panel)

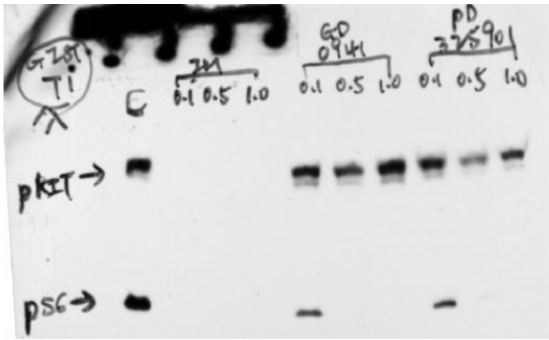

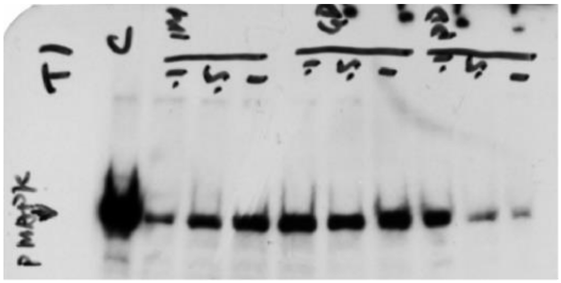

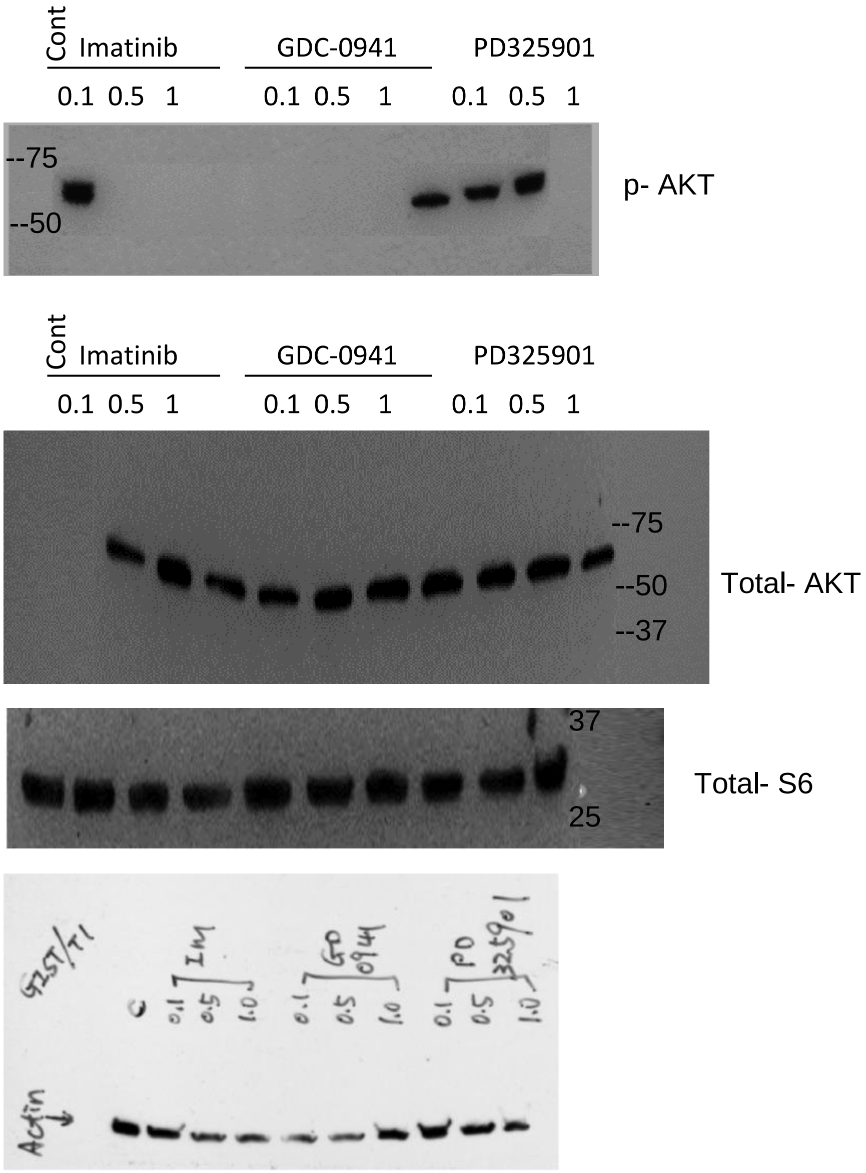
Cont
Imatinib
GDC-0941
PD325901
0.1 0.5 1 0.1 0.5 1 0.1 0.5 1
--75
--50
p- AKT
Cont
Imatinib
GDC-0941
PD325901
0.1 0.5 1 0.1 0.5 1 0.1 0.5 1
--75
--50
--37
Total- AKT
37
25
Total- S6
Actin

Fig. 1C---5R=GIST-T1/670 (middle panel)

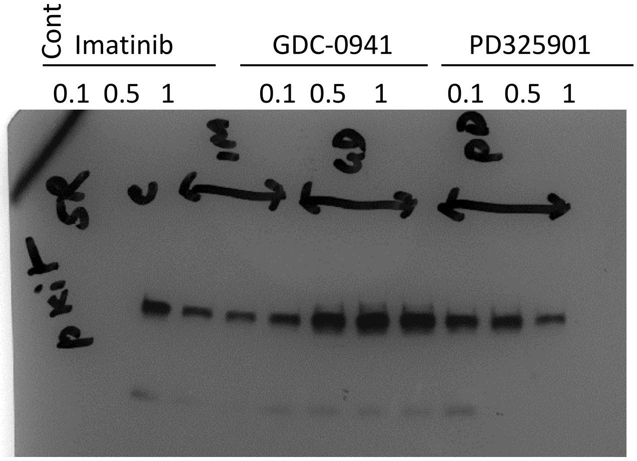

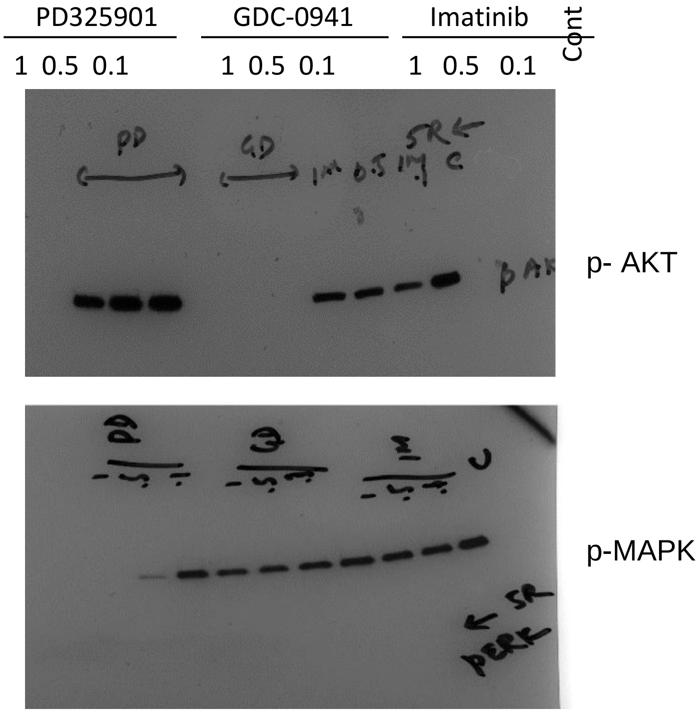

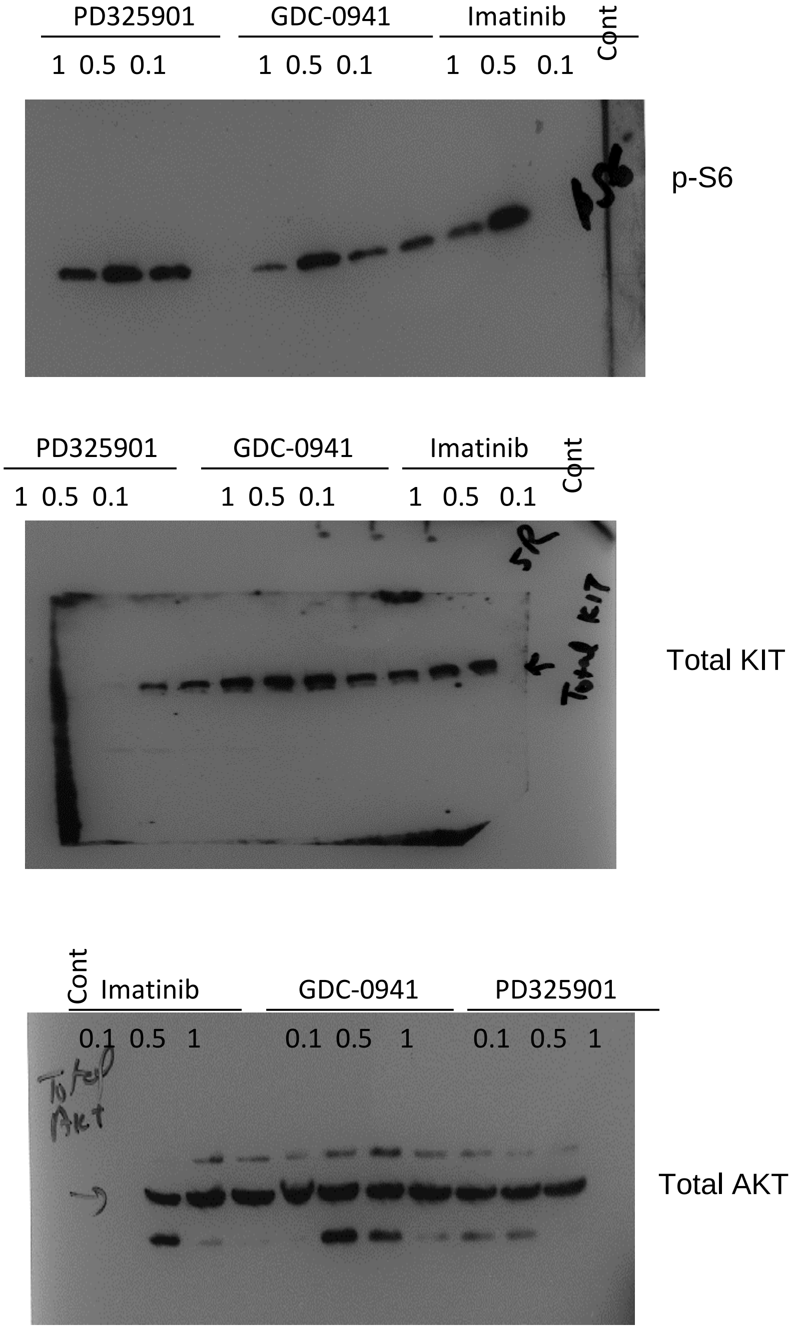
PD325901
GDC-0941
Imatinib
Cont
1 0.5 0.1
1 0.5 0.1
1 0.5 0.1
p-S6
PD325901
GDC-0941
Imatinib
Cont
1 0.5 0.1
1 0.5 0.1
1 0.5 0.1
Total KIT
Cont
Imatinib
GDC-0941
PD325901
0.1 0.5 1
0.1 0.5 1
0.1 0.5 1
Total AKT

Cont	Imatinib			GDC-0941			PD325901		
	0.1	0.5	1	0.1	0.5	1	0.1	0.5	1

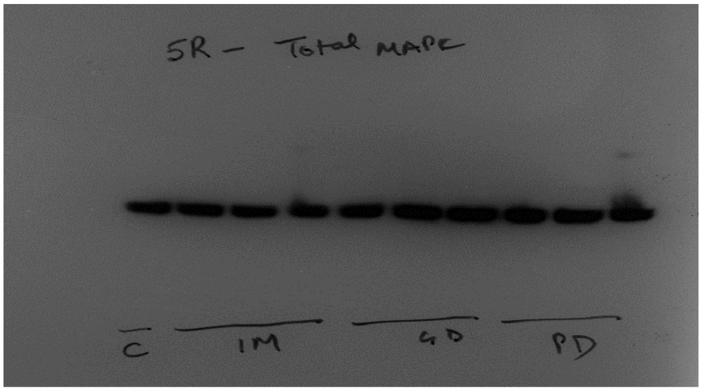

Total MAPK

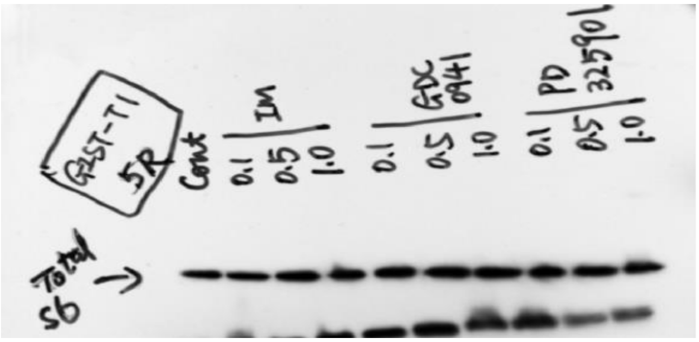

Total S6

Cont	Imatinib			GDC-0941			PD325901		
	0.1	0.5	1	0.1	0.5	1	0.1	0.5	1

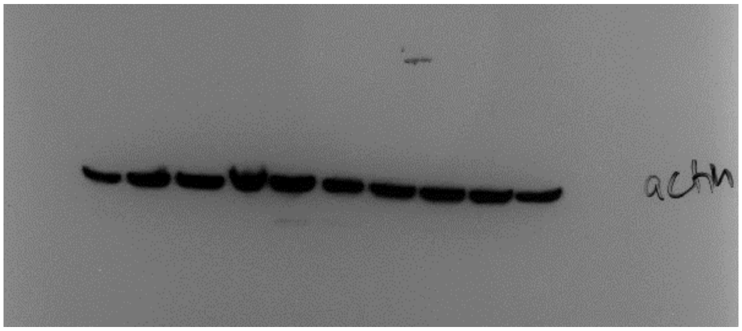

actin

Fig. 1C—GIST-T1/10R (right panel)

Dose response

p-KIT/P-MAPK

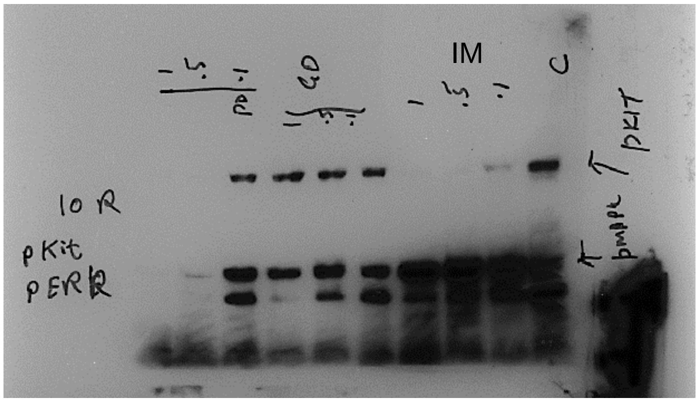

p-KIT

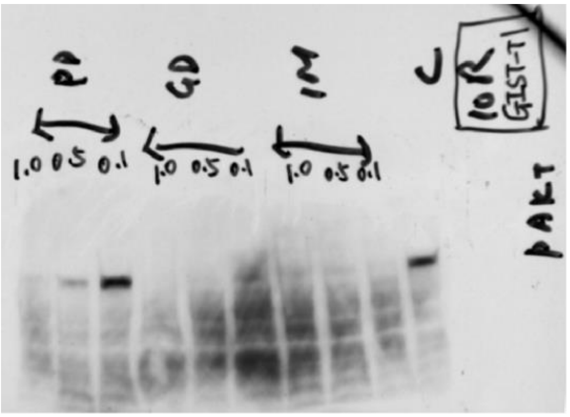

p-AKT

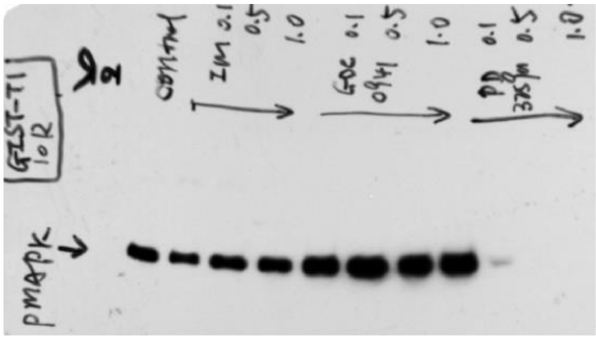

p-MAPK

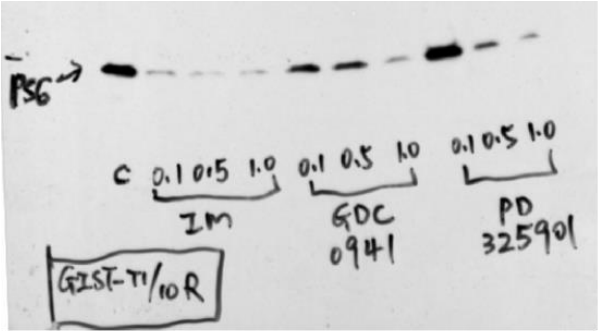

p-S6

GIST-T1/10R—Fig.1C

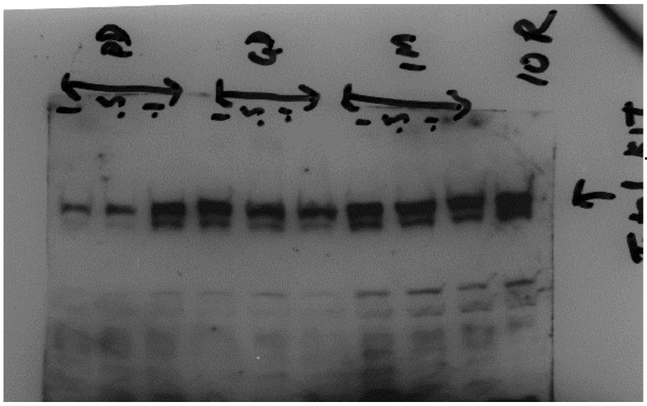

Total KIT

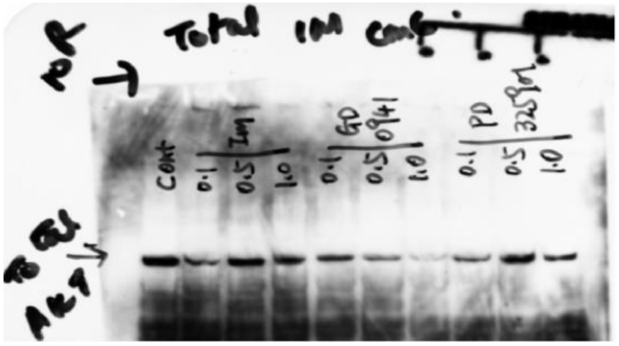

Total AKT

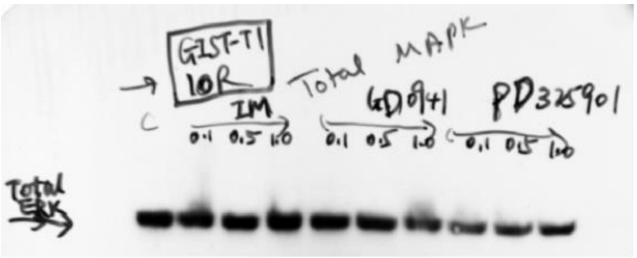

Total MAPK

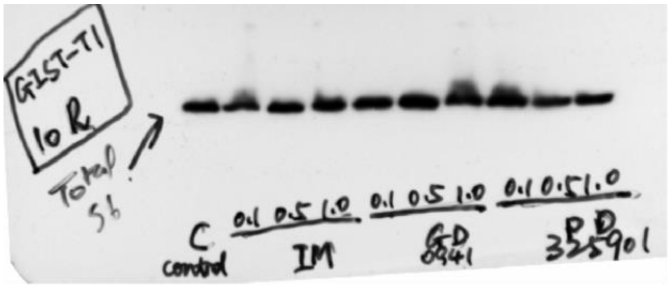

Total S6

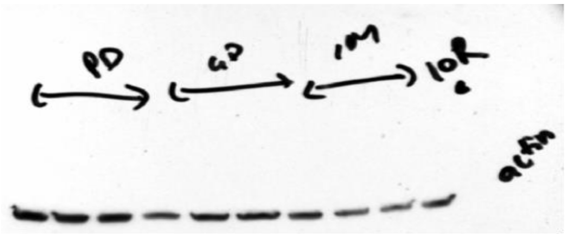

actin

Fig.3

GIST-T1-Imatinib

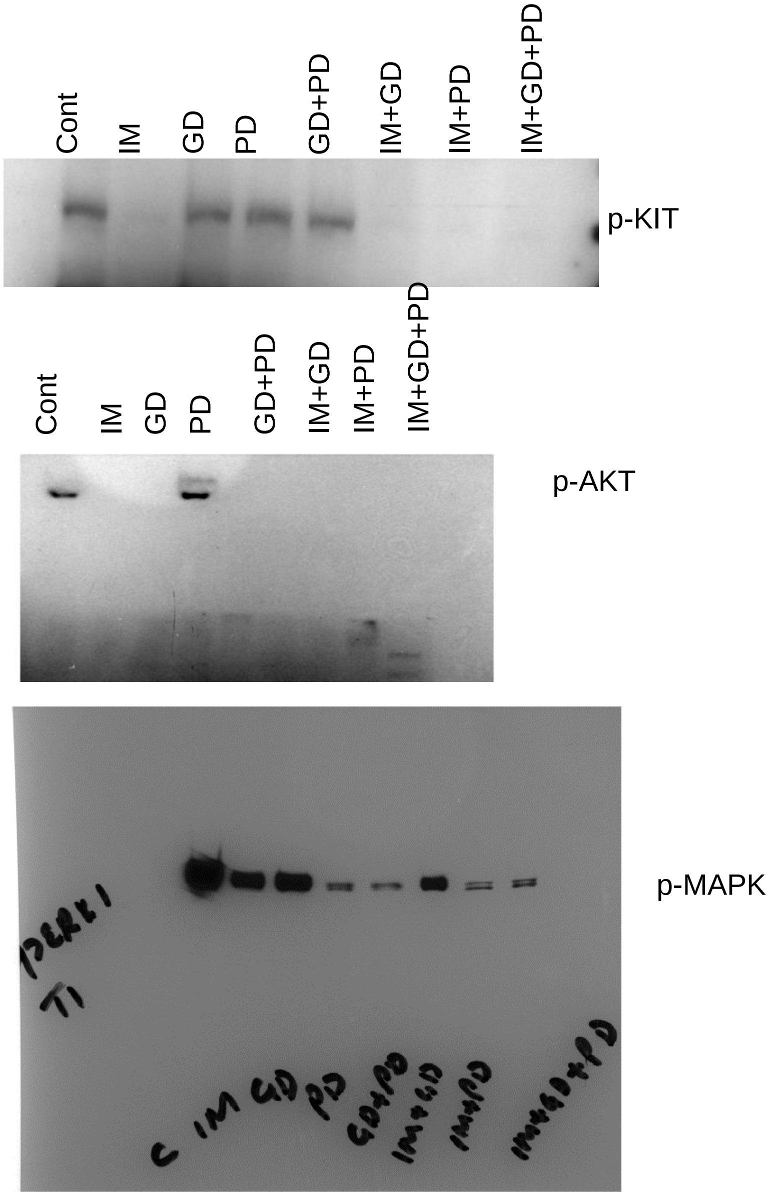

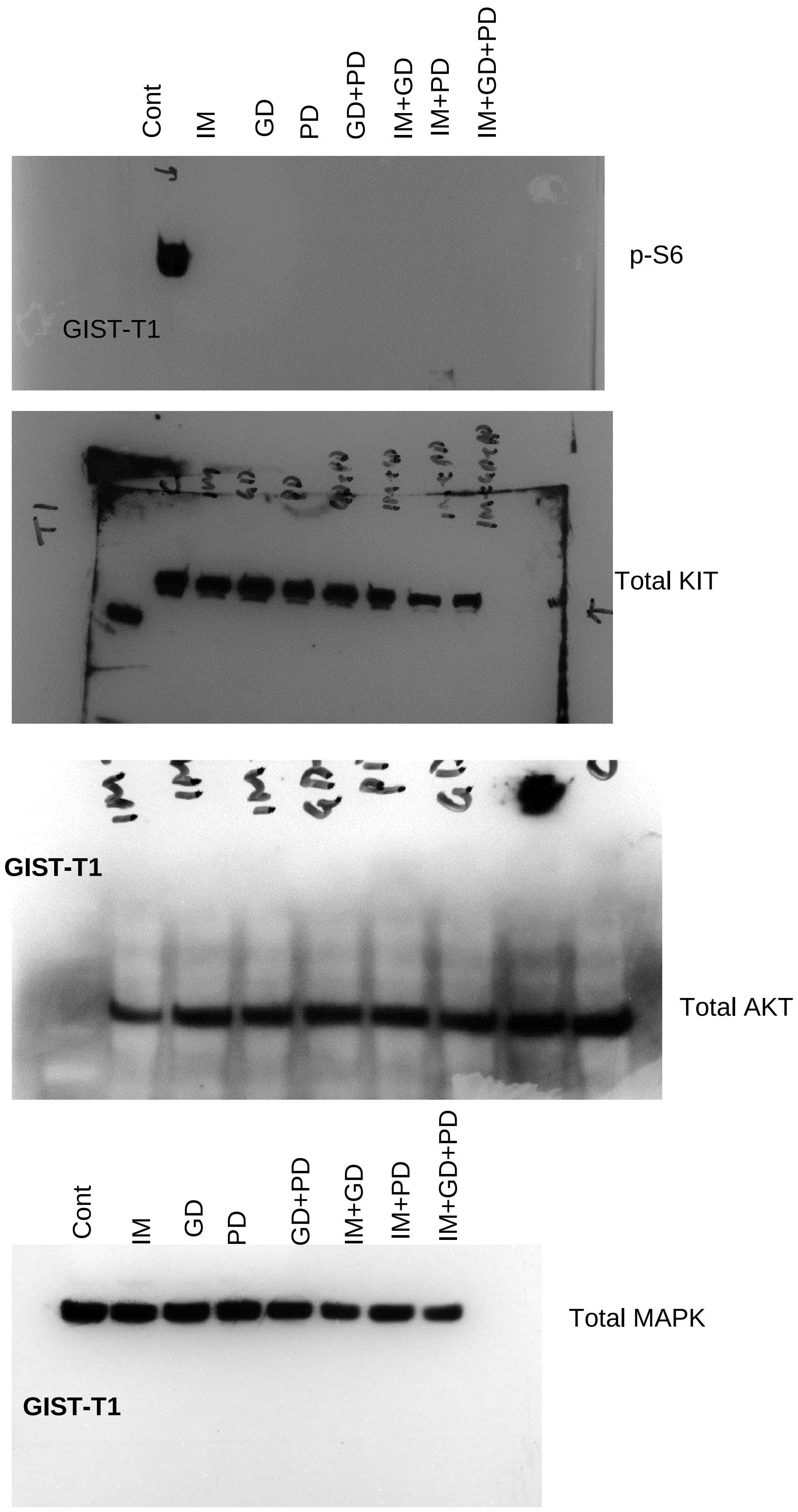
Cont
IM
GD
PD
GD+PD
IM+GD
IM+PD
IM+GD+PD
p-S6
GIST-T1
Total KIT
GIST-T1
Total AKT
Cont
IM
GD
PD
GD+PD
IM+GD
IM+PD
IM+GD+PD
Total MAPK
GIST-T1

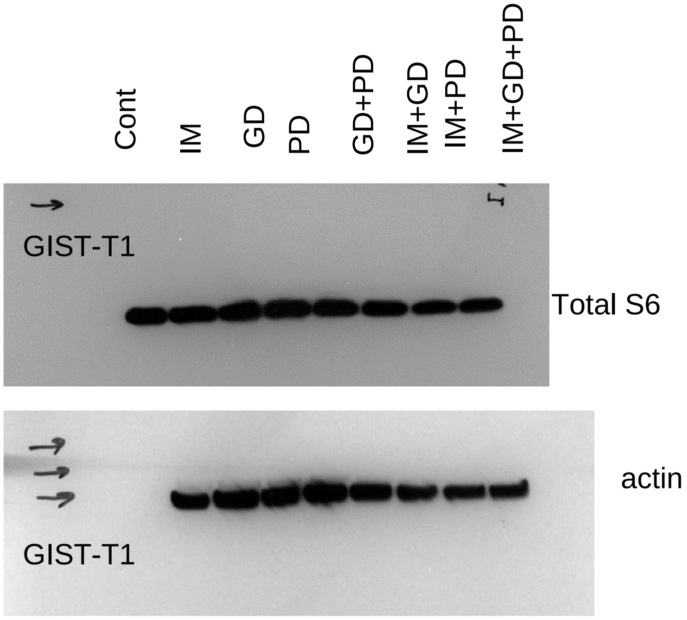

Fig. 3--GIST882 (2nd panel)

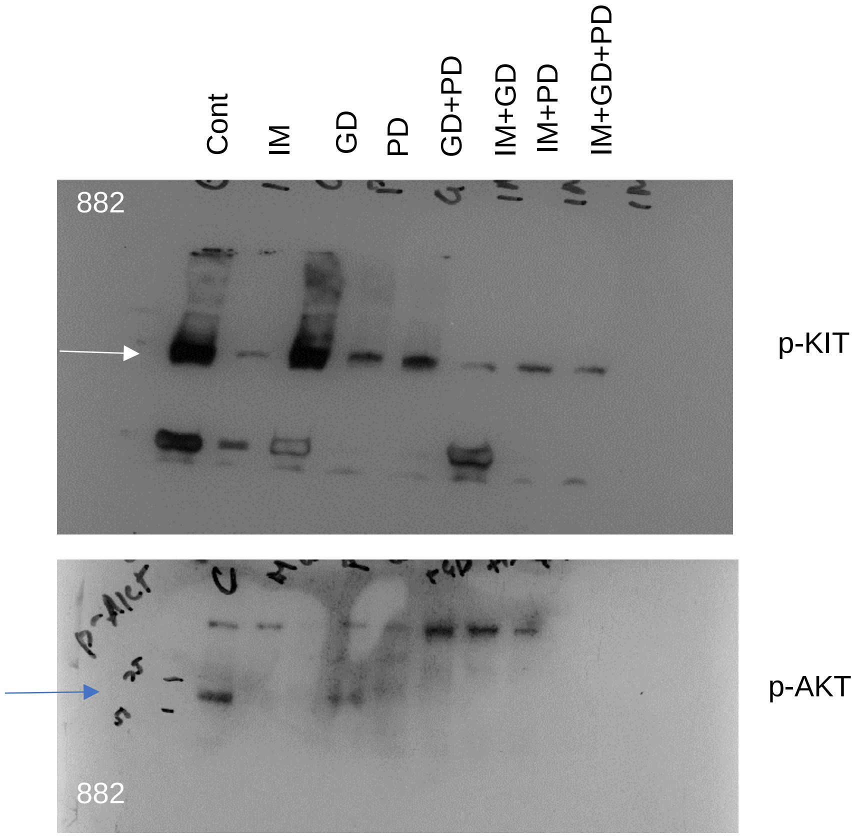

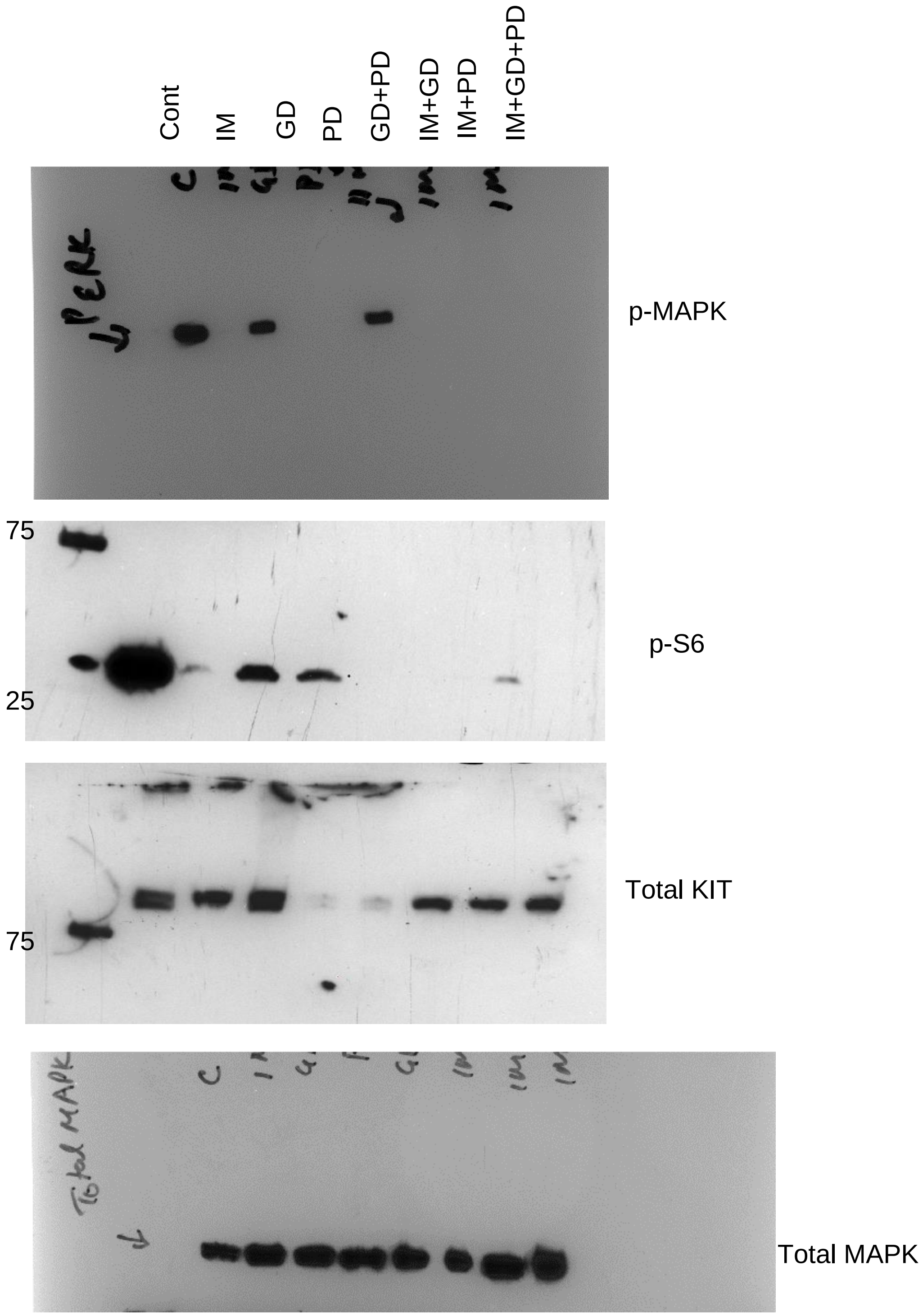
Cont
IM
GD
PD
GD+PD
IM+GD
IM+PD
IM+GD+PD
p-MAPK
75
p-S6
25
Total KIT
75
Total MAPK

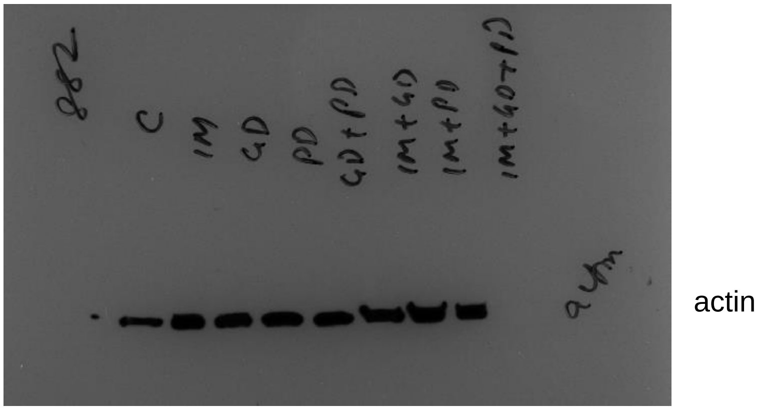

Fig.3 GIST-T1/670 (5R) 3rd panel

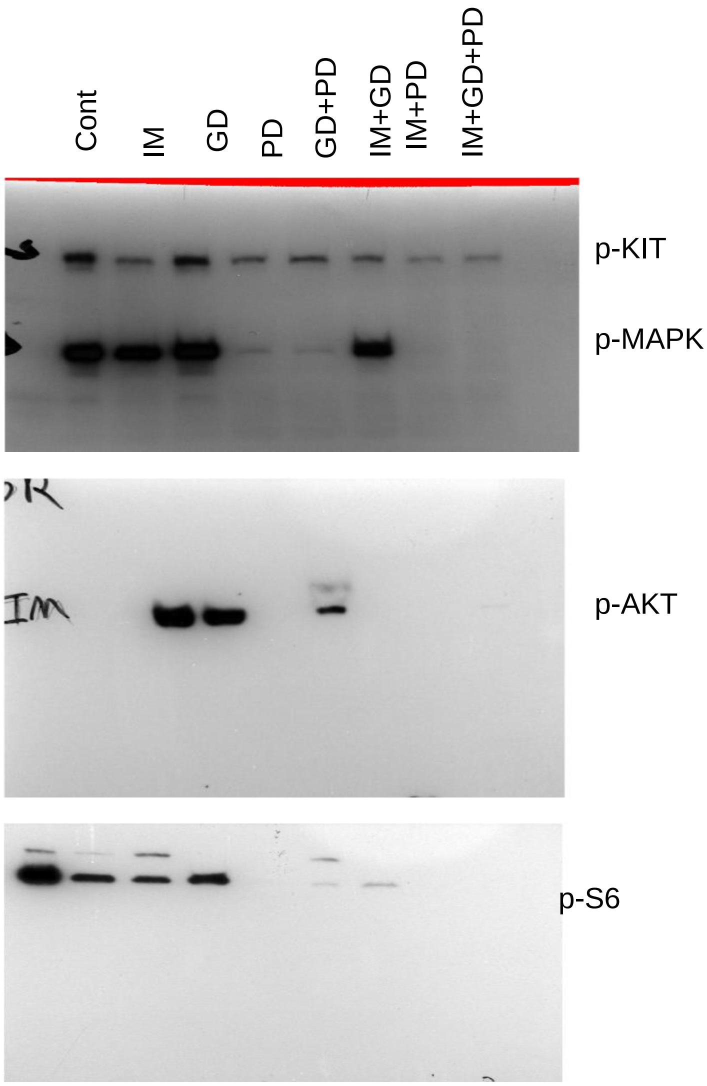

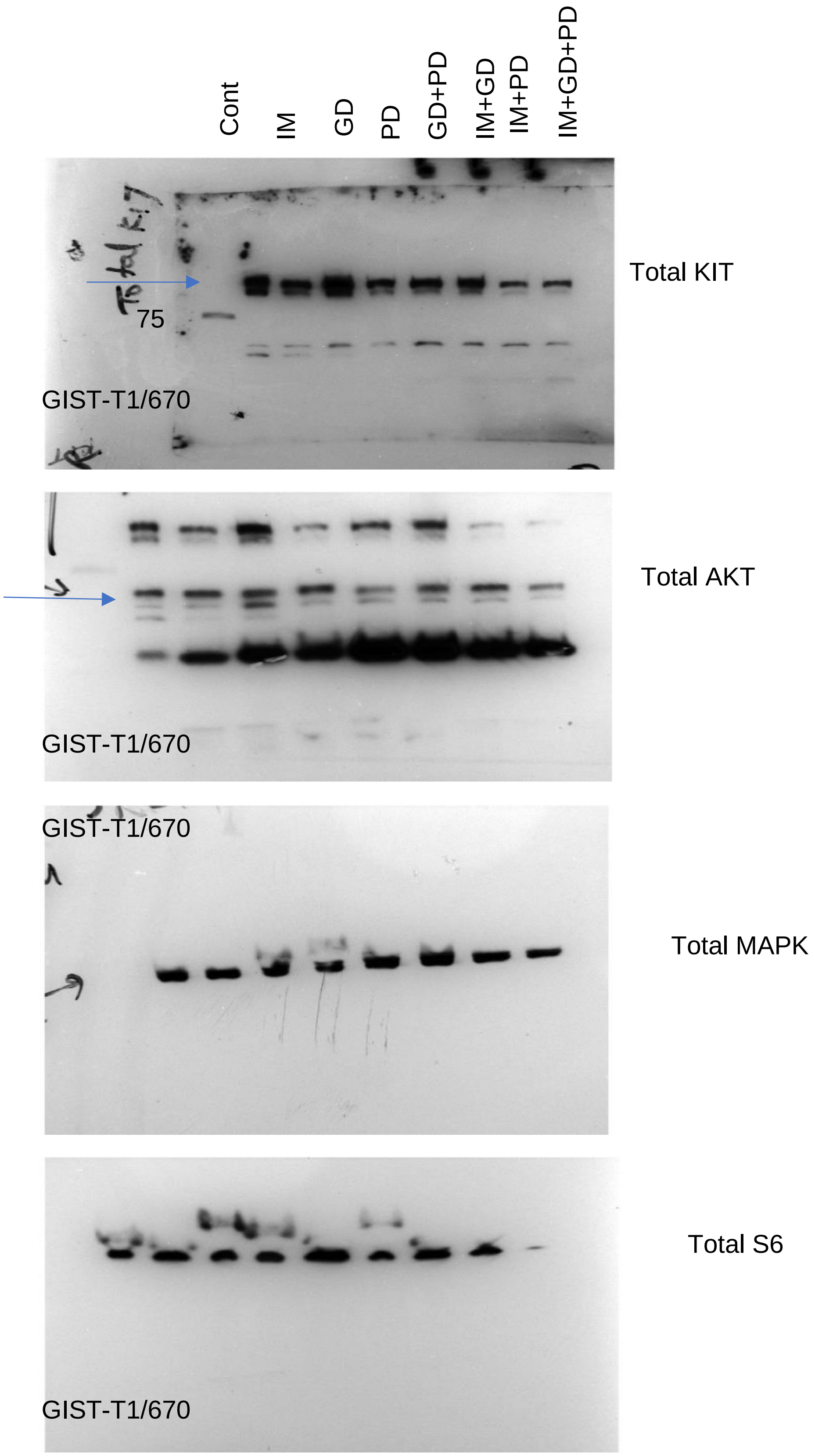
Cont
IM
GD
PD
GD+PD
IM+GD
IM+PD
IM+GD+PD
Total KIT
75
GIST-T1/670
Total AKT
GIST-T1/670
GIST-T1/670
Total MAPK
Total S6
GIST-T1/670

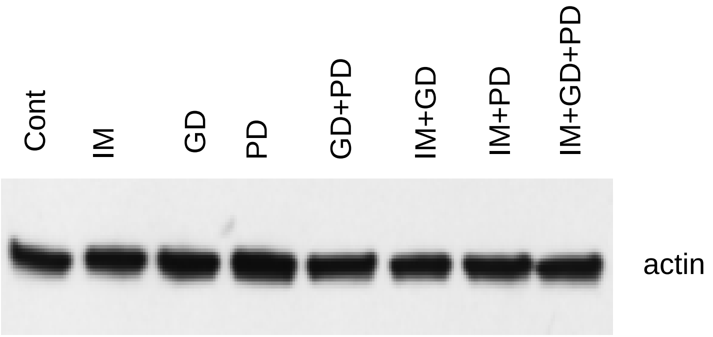

Fig. 3-GIST-T1/10R

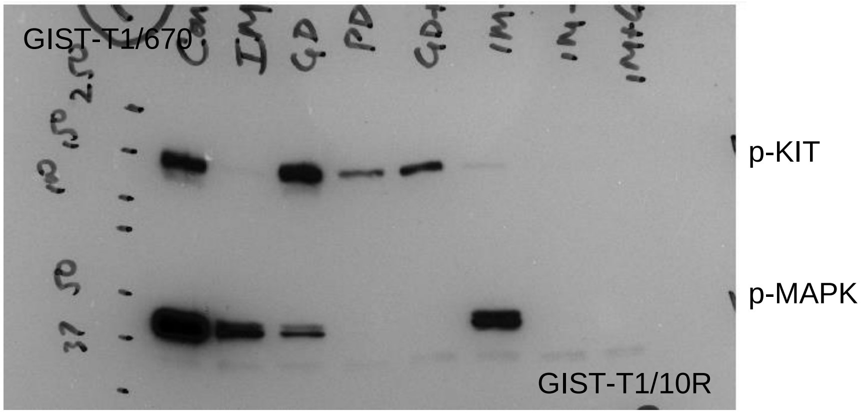

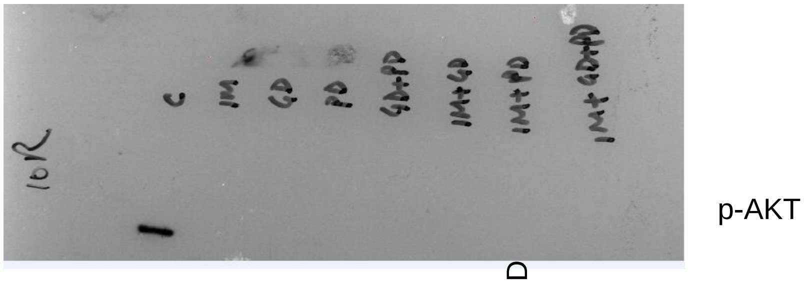

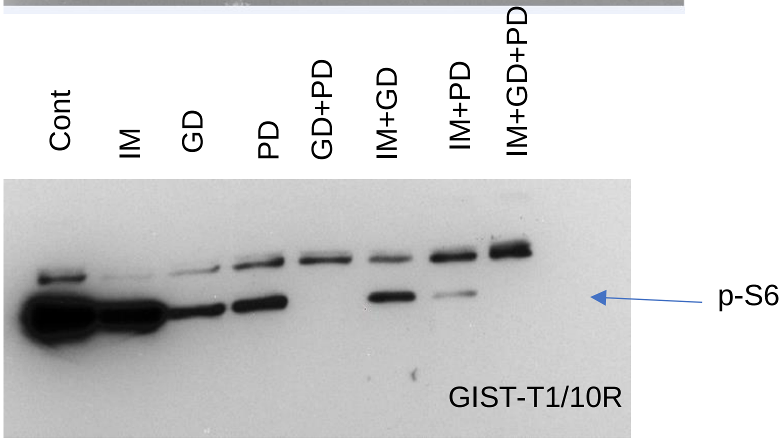

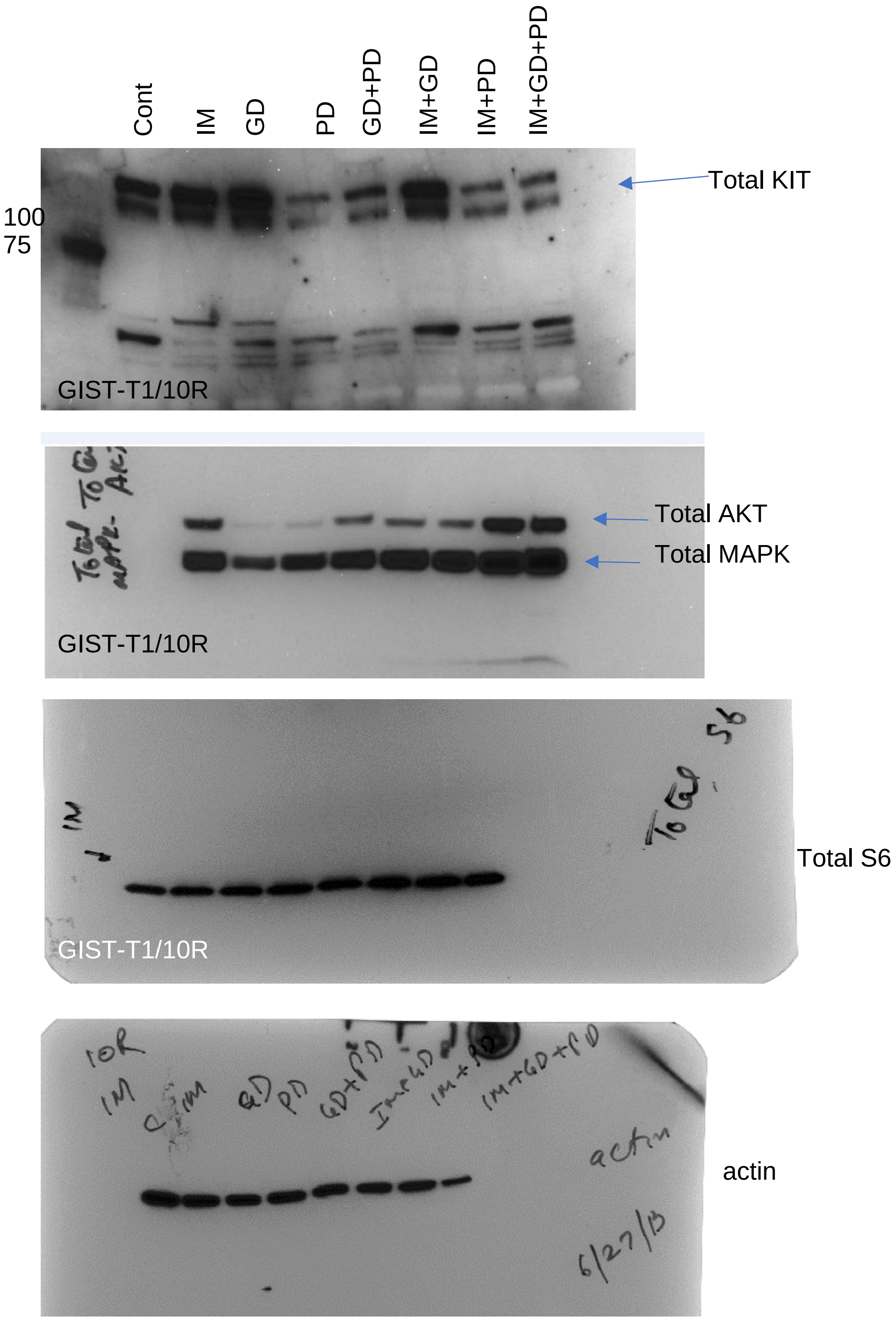

Cont
IM
GD
PD
GD+PD
IM+GD
IM+PD
IM+GD+PD
100
75
Total KIT
GIST-T1/10R
Total AKT
Total MAPK
GIST-T1/10R
Total S6
GIST-T1/10R
actin

Fig. 4B

GIST-T1

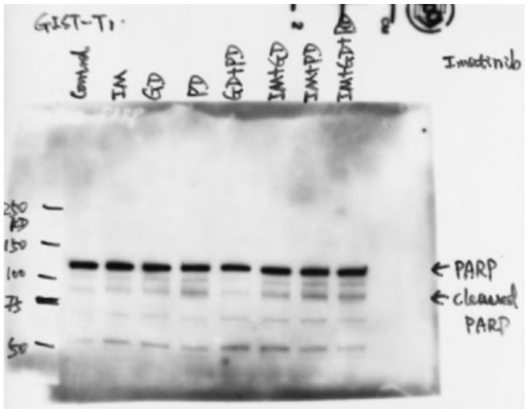

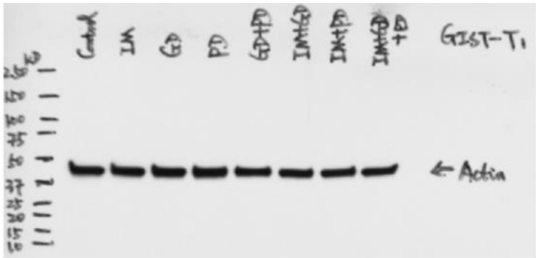

GIST-T1/670

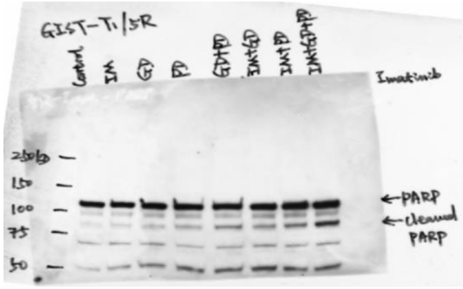

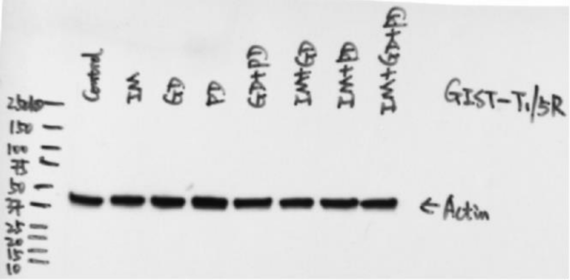

GIST-T1/10R

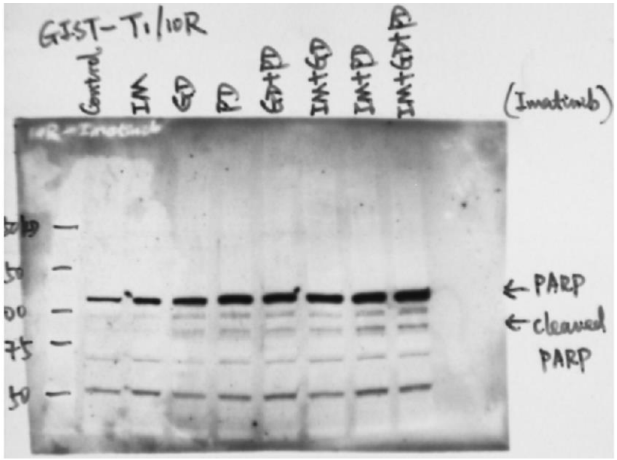
GIST-T1/10R
Control
IM
GD
PD
GD+PD
IM+GD
IM+PD
IM+GD+PD
(Imatinib)
← PARP
← cleaved PARP
75
50

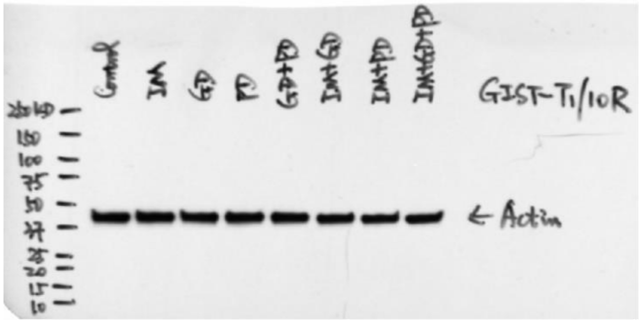
Control
IM
GD
PD
GD+PD
IM+GD
IM+PD
IM+GD+PD
GIST-T1/10R
150
100
75
50
37
25
20
15
10
← Actin

Supplementary figures

S1C Fig

GIST882

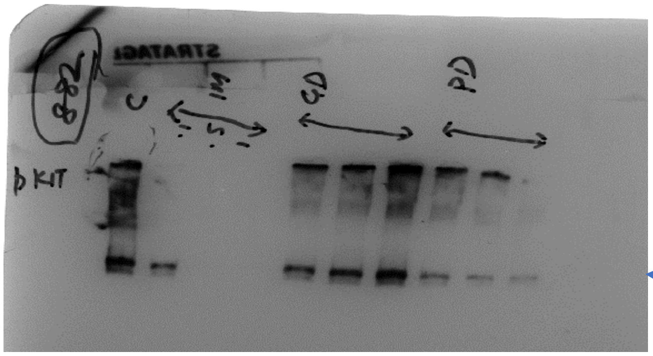

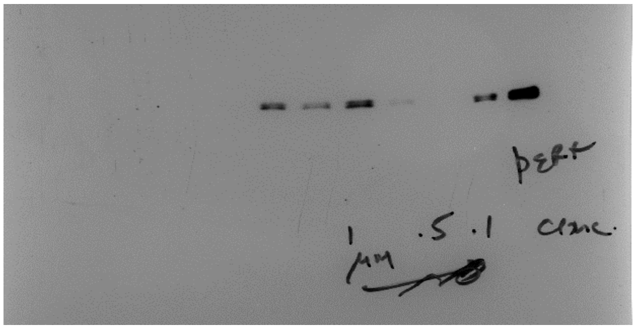

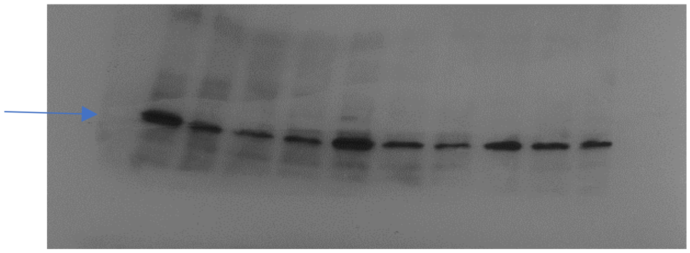

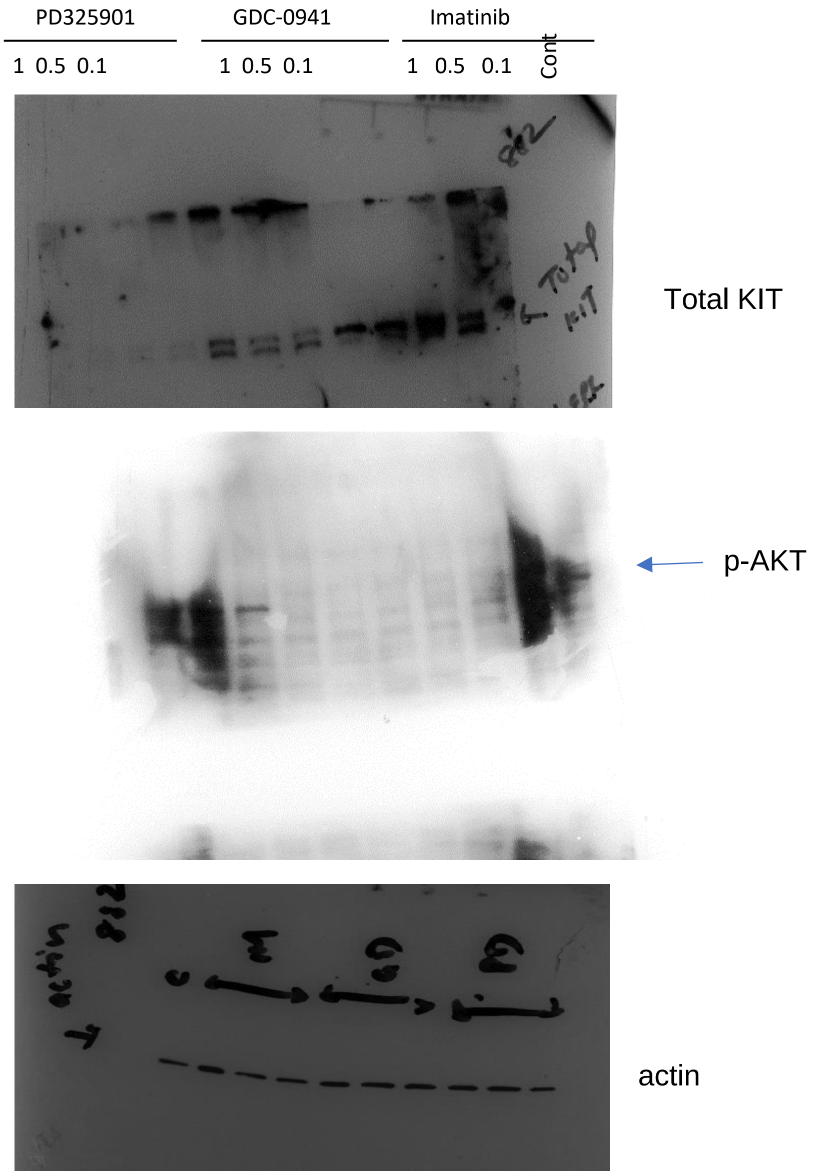
PD325901
GDC-0941
Imatinib
1 0.5 0.1
1 0.5 0.1
1 0.5 0.1
Cont
Total KIT
p-AKT
actin

S3A Fig

GIST-T1-sunitinib

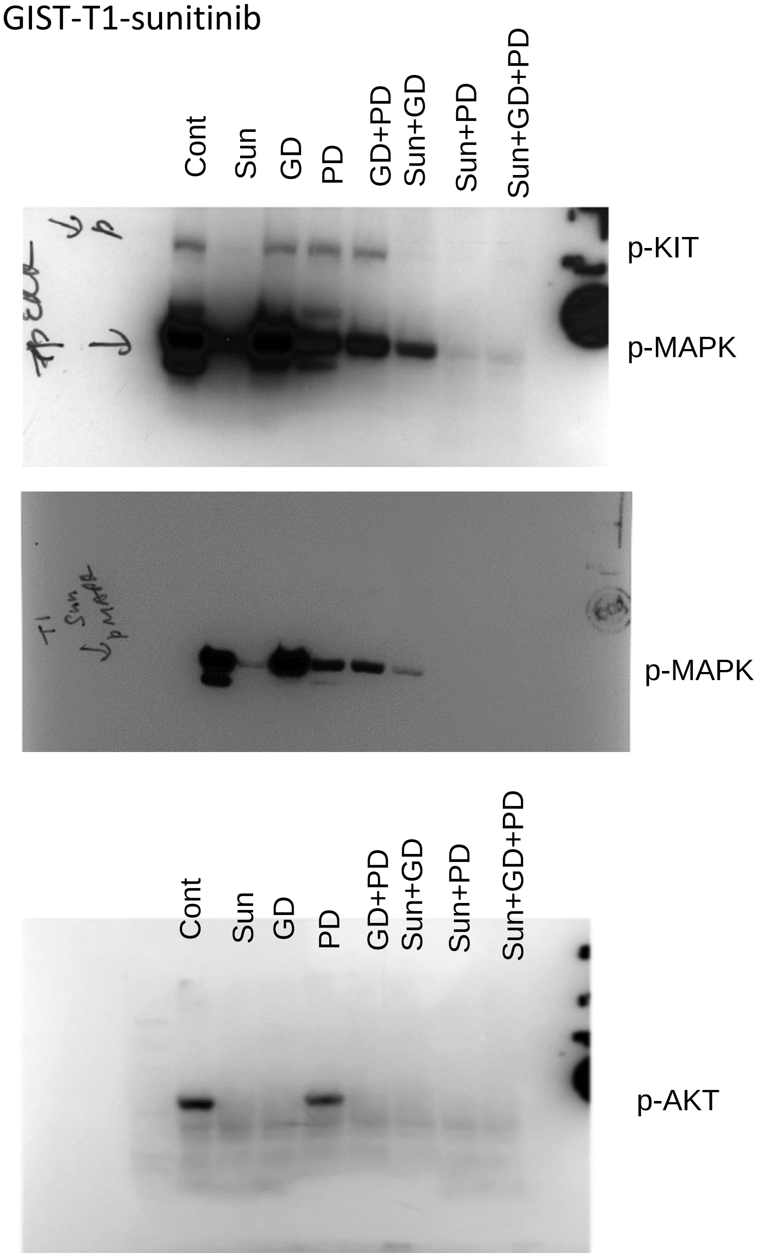

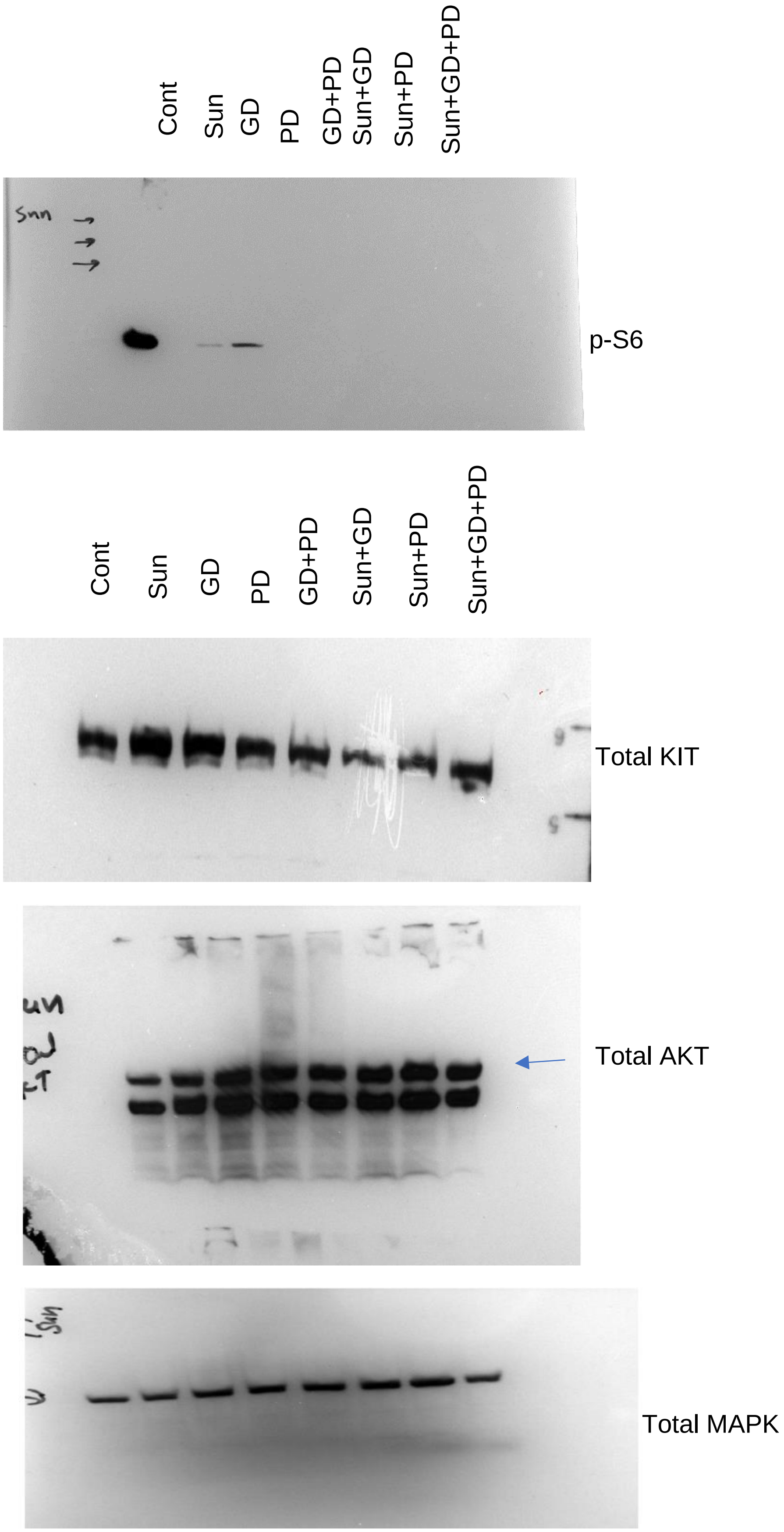
Cont
Sun
GD
PD
GD+PD
Sun+GD
Sun+PD
Sun+GD+PD
p-S6
Cont
Sun
GD
PD
GD+PD
Sun+GD
Sun+PD
Sun+GD+PD
Total KIT
Total AKT
Total MAPK

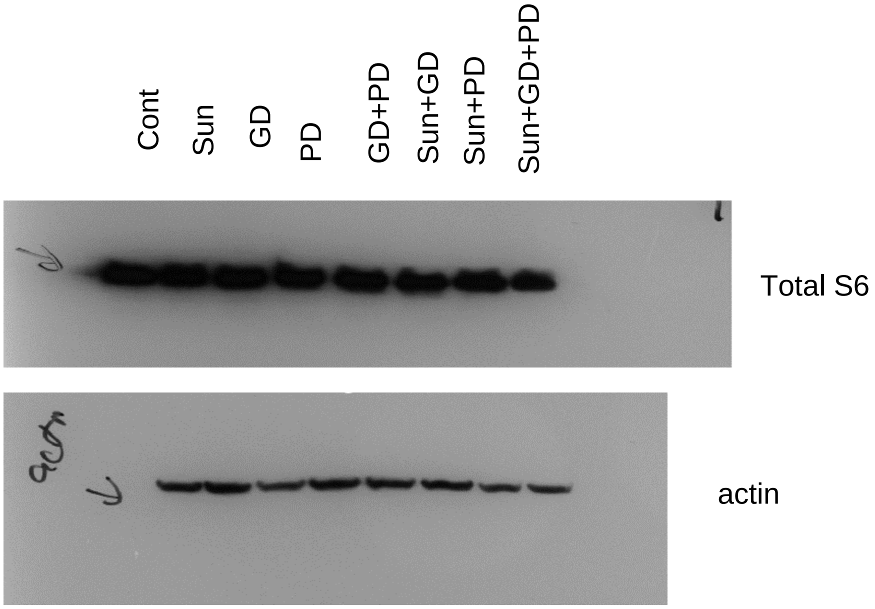

GIST 882-sunitinib (2nd panel)

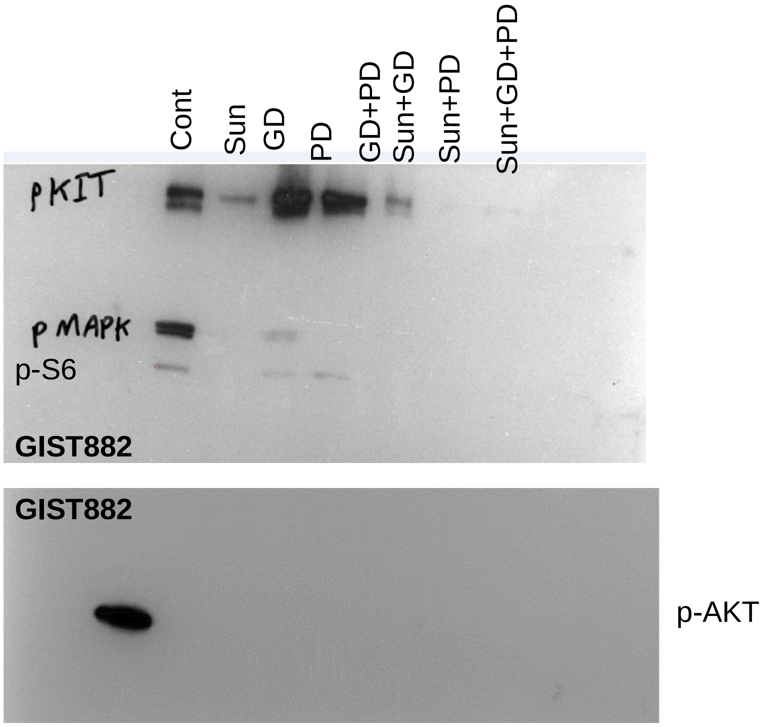

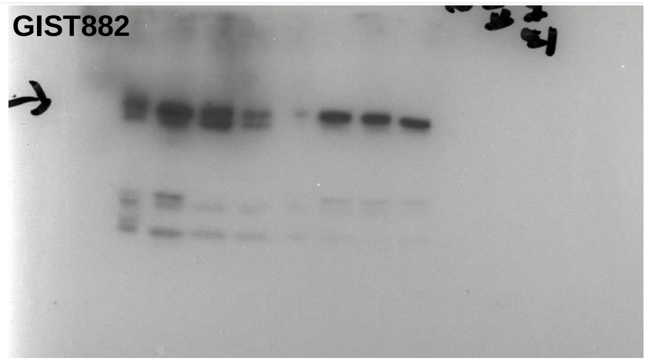

Total KIT

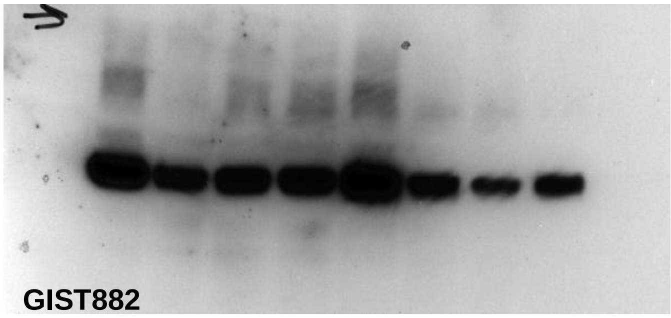

Total AKT

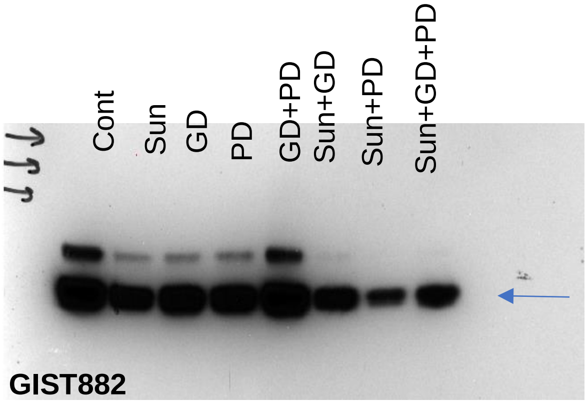

Total MAPK

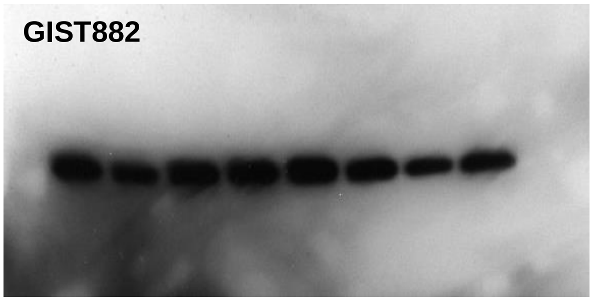

Total S6

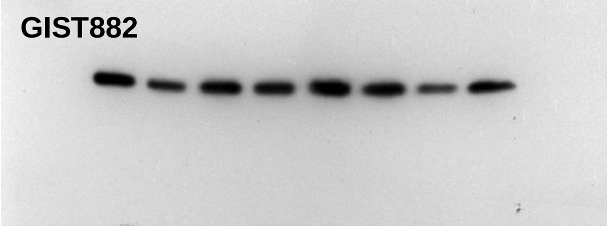

actin

GIST-T1/670--sunitinib

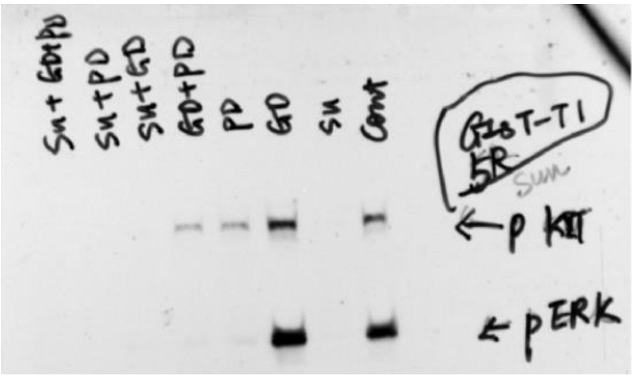

p-MAPK

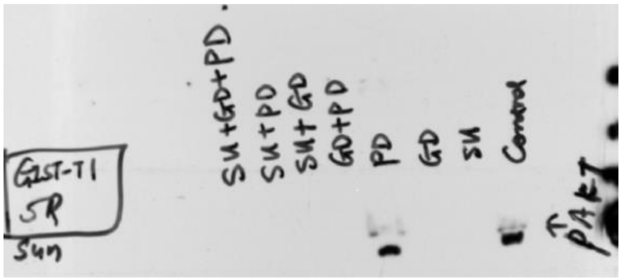

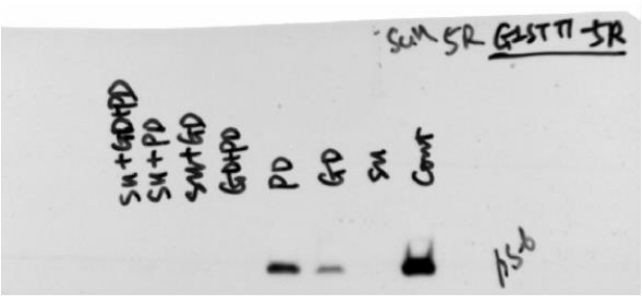

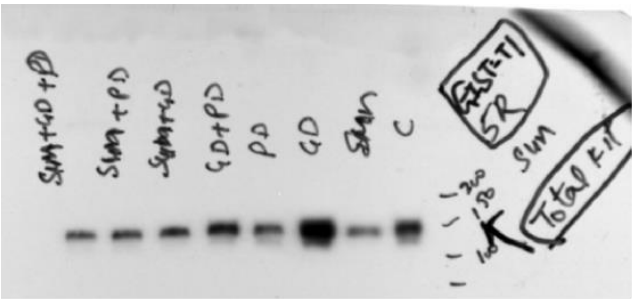

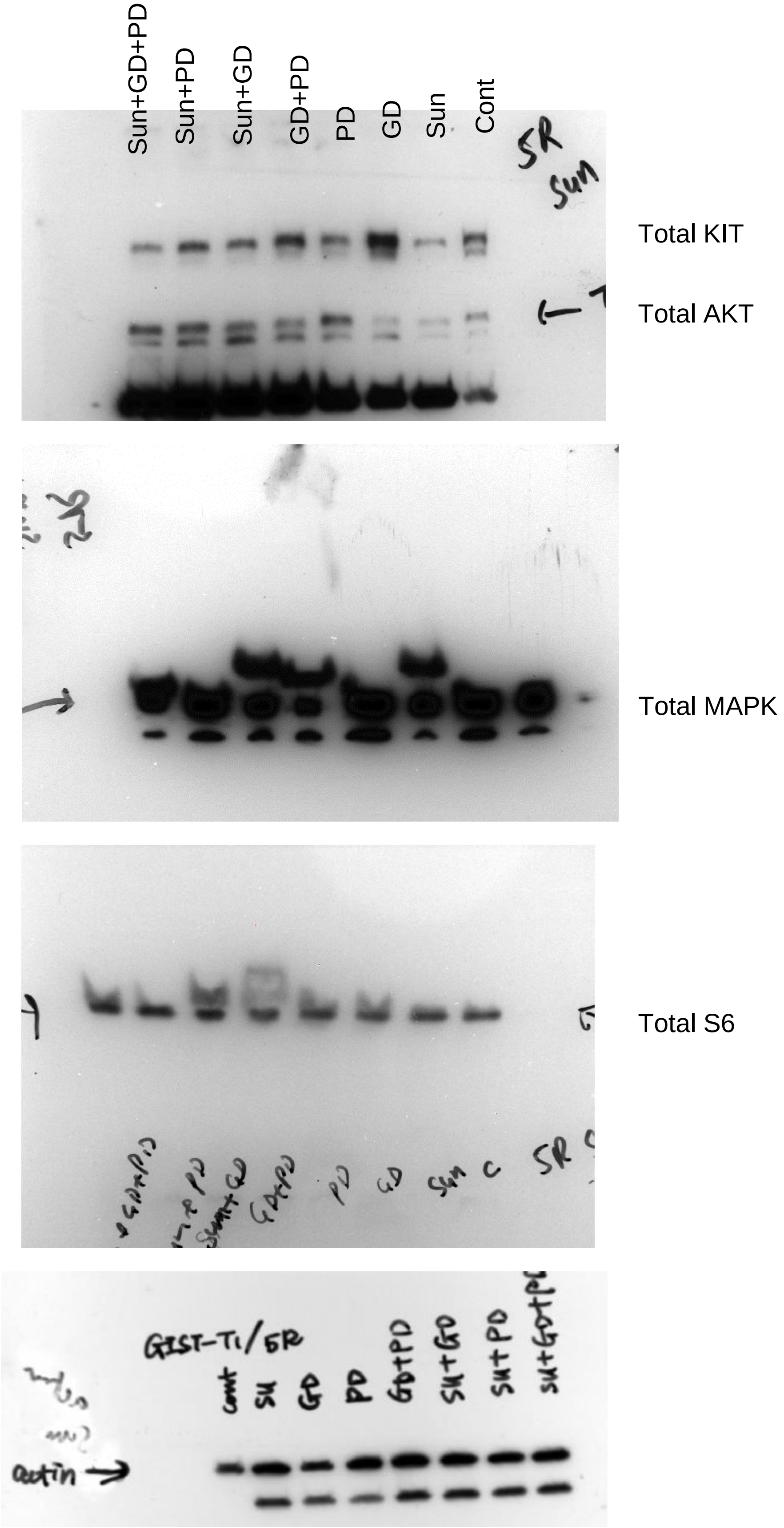

Sun+GD+PD
Sun+PD
Sun+GD
GD+PD
PD
GD
Sun
Cont
Total KIT
Total AKT
Total MAPK
Total S6
GIST-T1/5R
actin

GIST-T1/10R-sunitinib

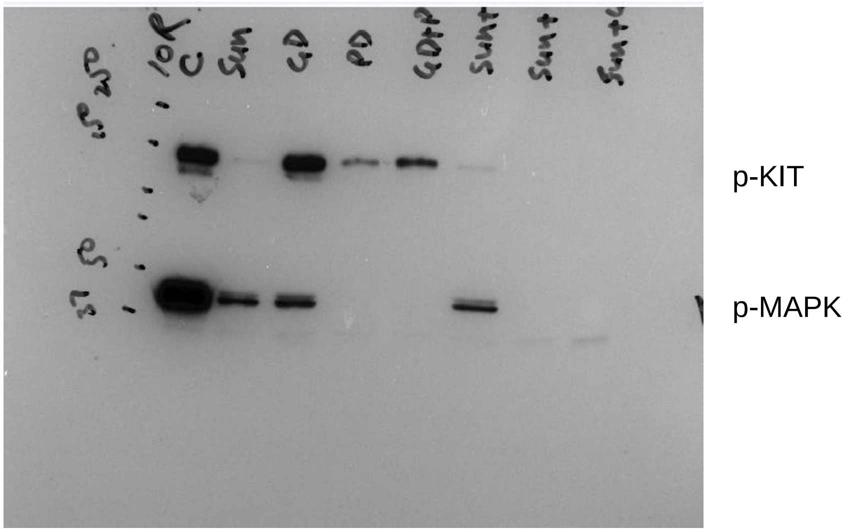

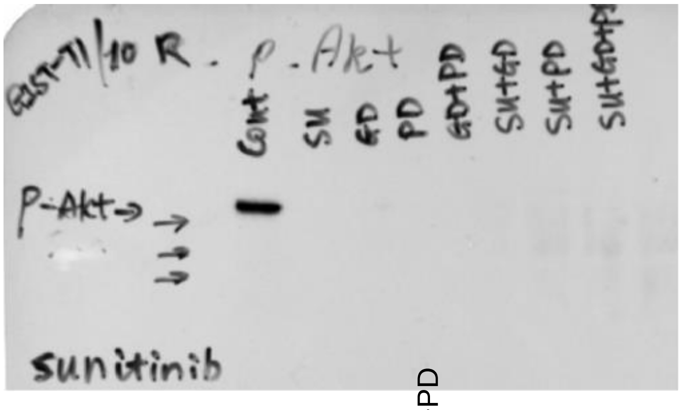

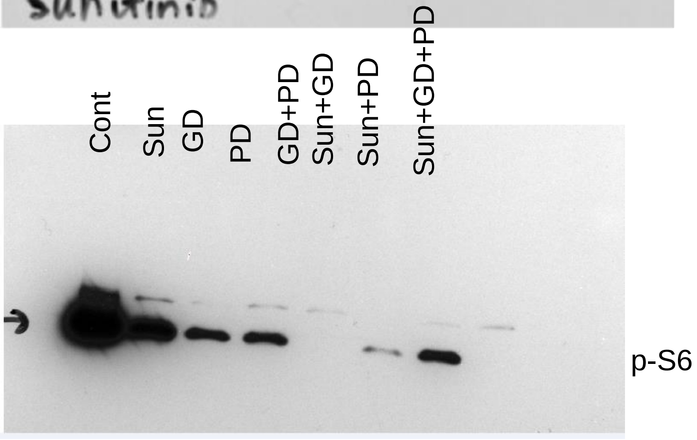

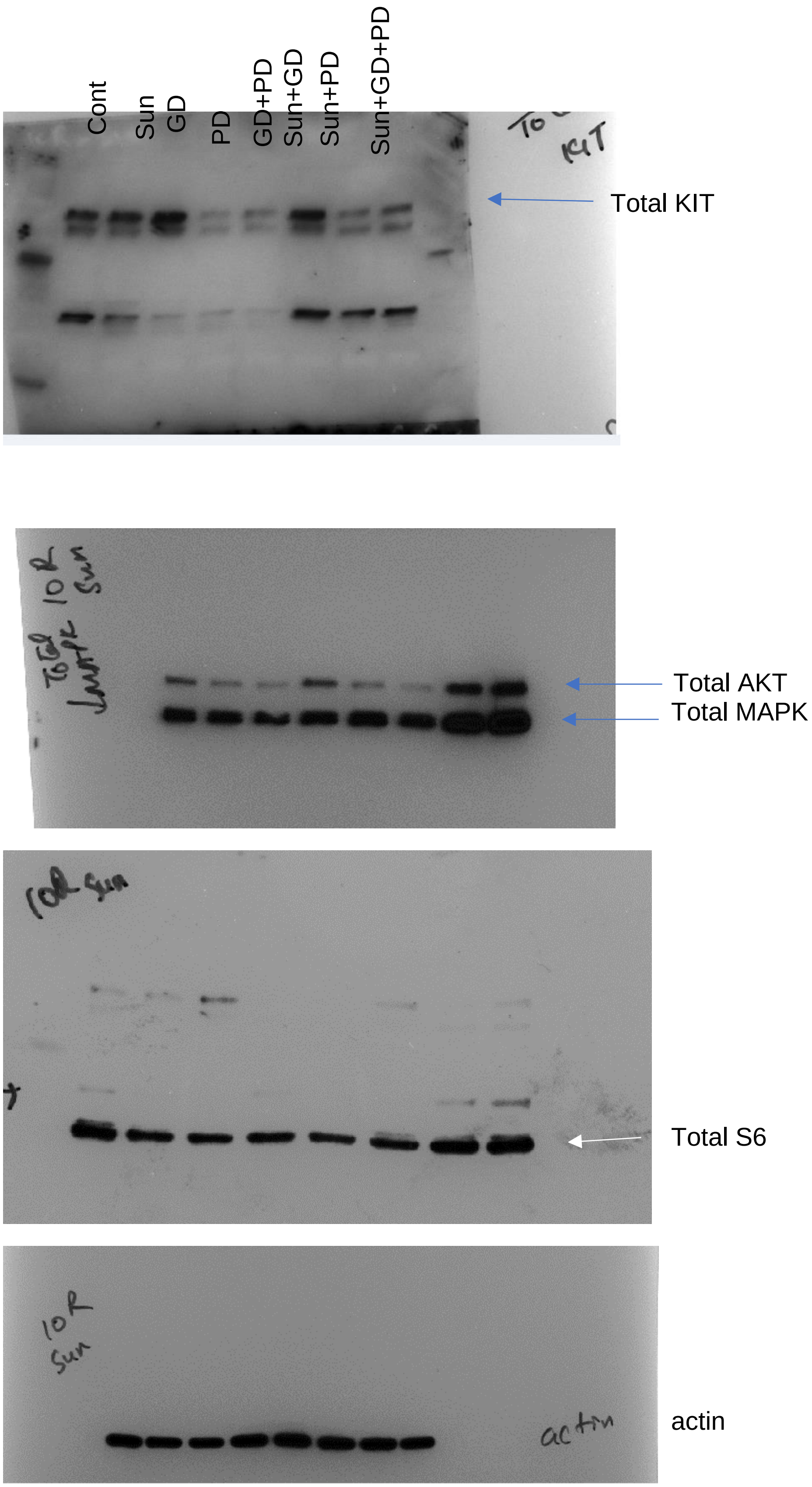

Cont
Sun
GD
PD
GD+PD
Sun+GD
Sun+PD
Sun+GD+PD
Total KIT
Total AKT
Total MAPK
Total S6
actin

S3A Fig GIST-T1-regorafenib

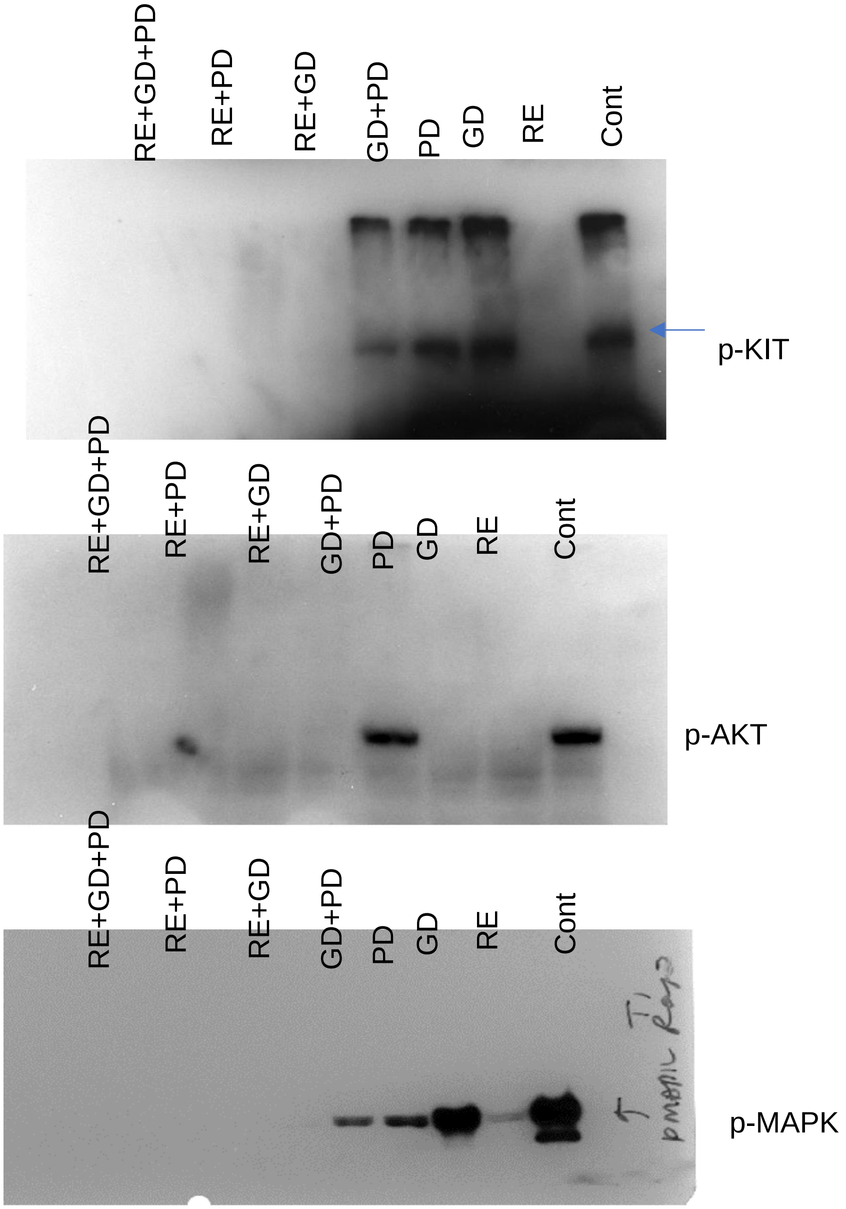

RE+GD+PD RE+PD RE+GD GD+PD PD GD RE Cont

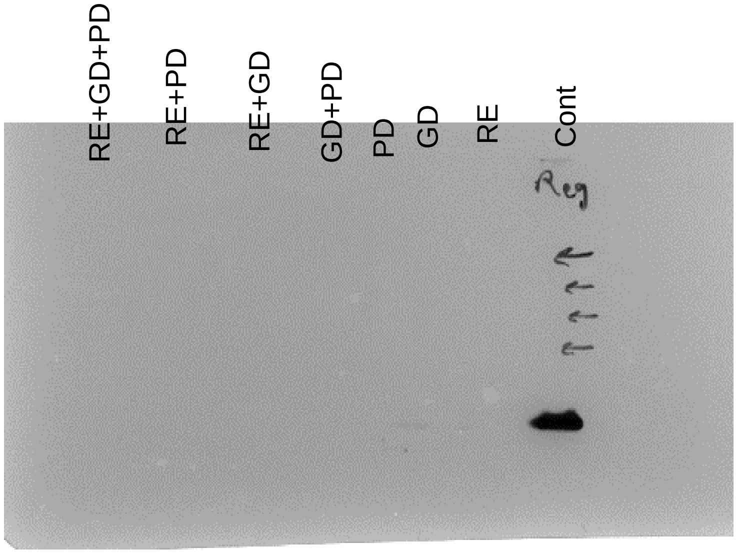

p-S6

Cont RE GD PD GD+PD RE+GD RE+PD RE+GD+PD

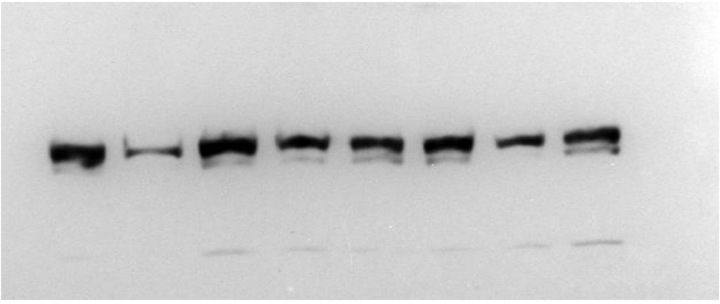

Total KIT

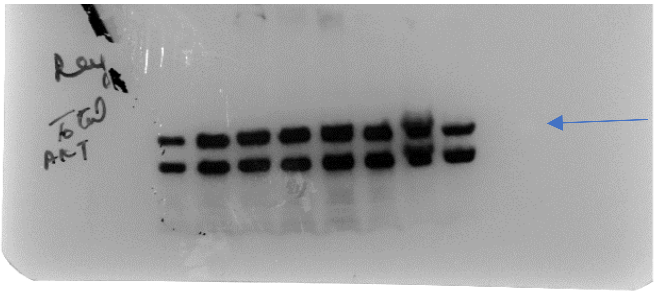

Total AKT

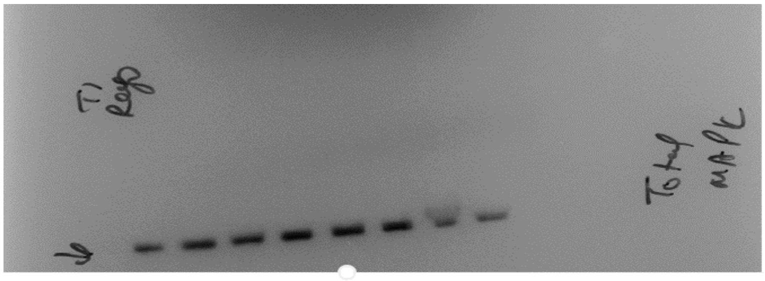

Total MAPK

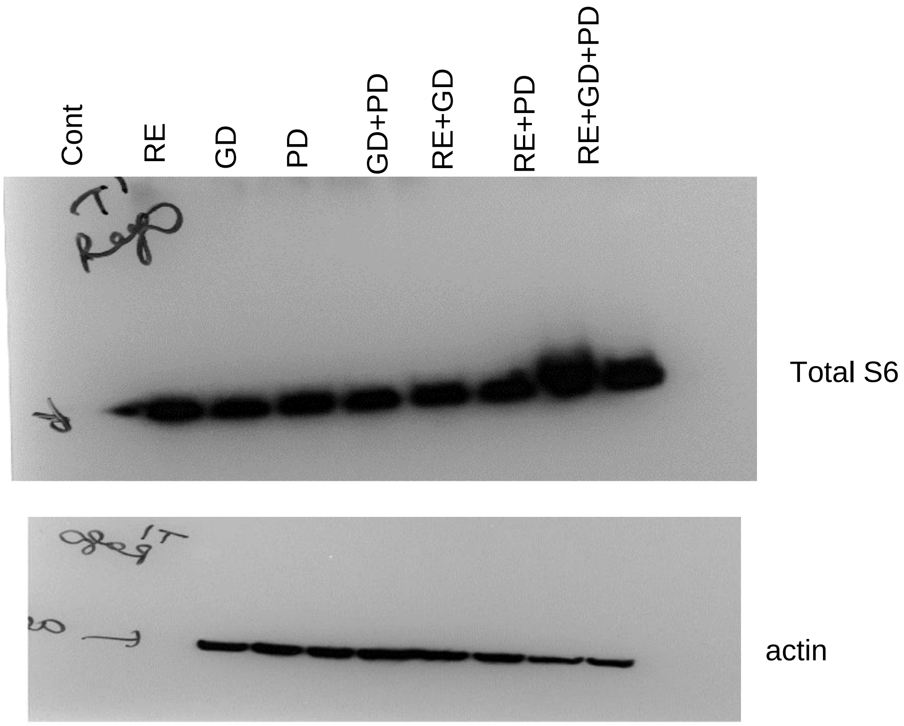

GIST882-regorafenib

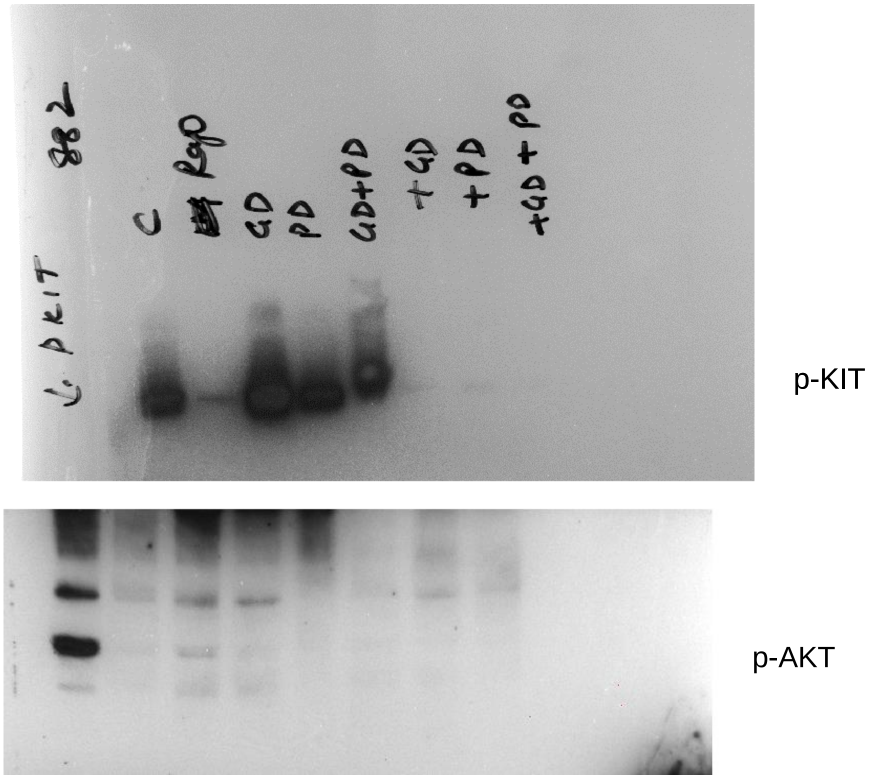

Cont RE GD PD GD+PD RE+GD RE+PD RE+GD+PD

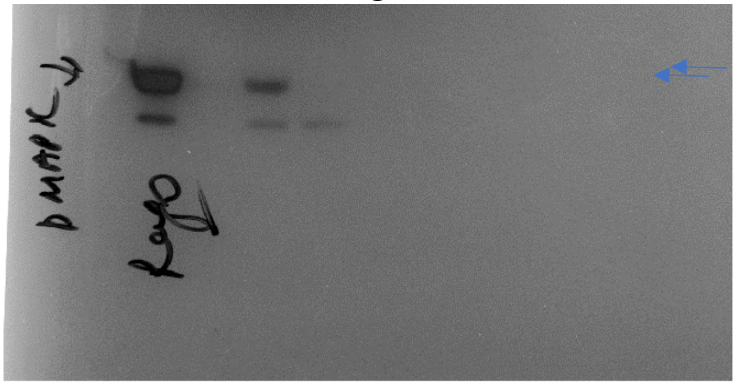

p-MAPK

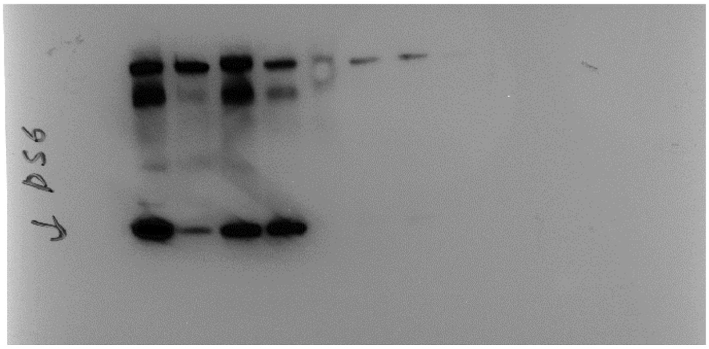

p-S6

RE+GD+PD RE+PD RE+GD GD+PD PD GD RE Cont

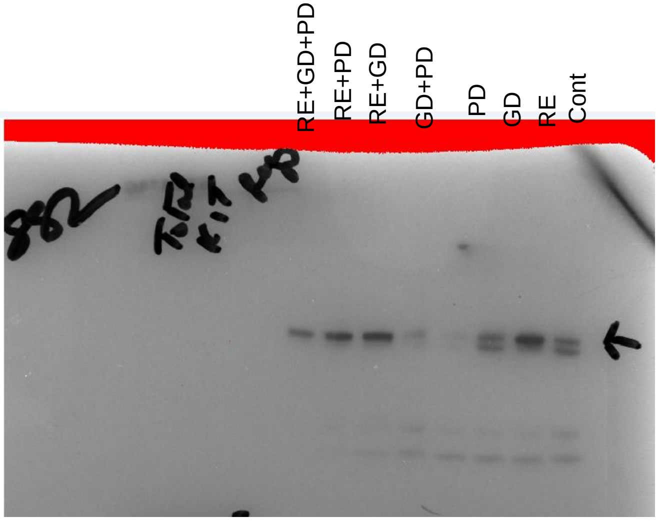

Total KIT

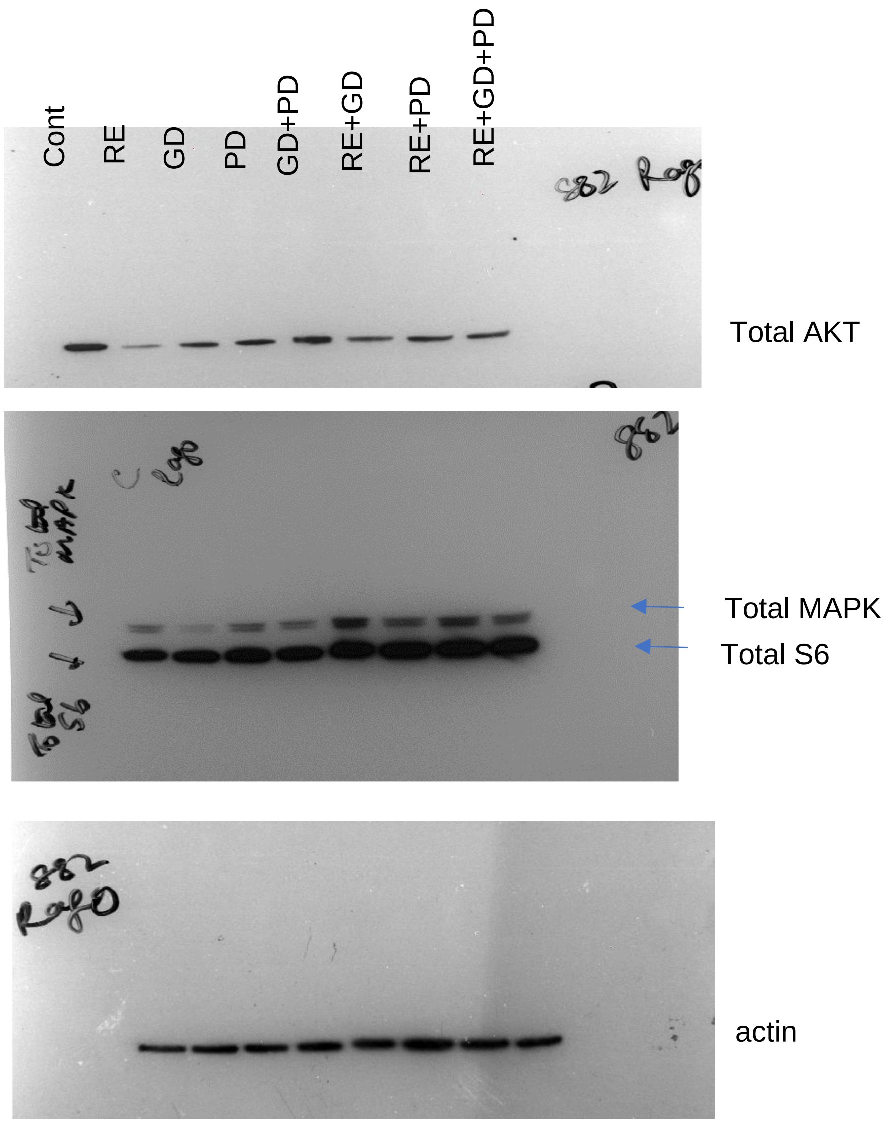

S3B Fig

GIST-T1/670 (5R)-Regorafenib

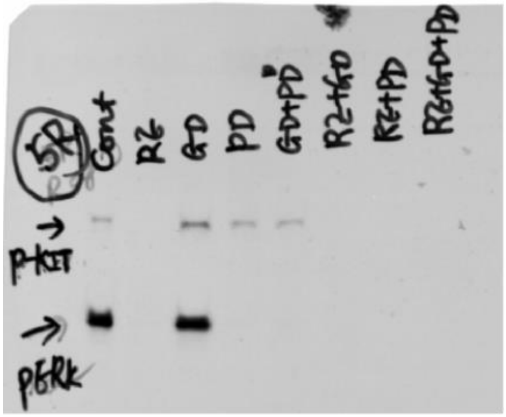

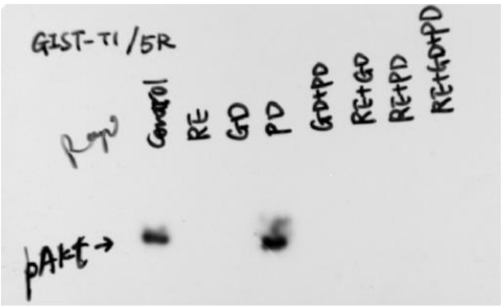
GIST-T1/5R
Control
RE
GD
PD
GD+PD
RE+GD
RE+PD
RE+GD+PD
pAkt →

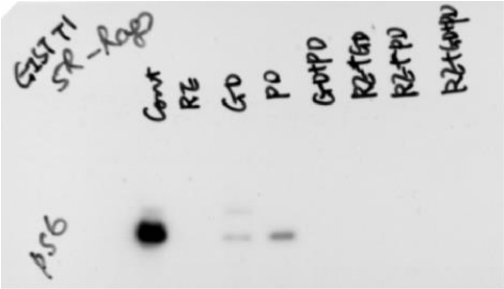
GIST-T1
5R-Rap
Cont
RE
GD
PD
GD+PD
RE+GD
RE+PD
RE+GD+PD
pS6

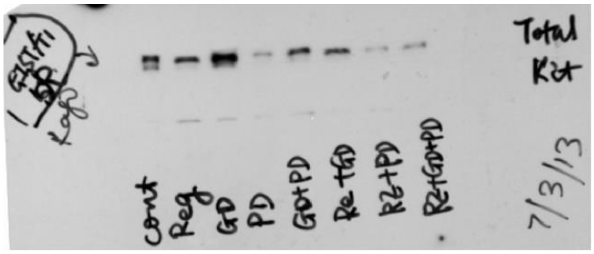
Total
Kit
Cont
Reg
GD
PD
GD+PD
Re+GD
RE+PD
RE+GD+PD
7/3/13

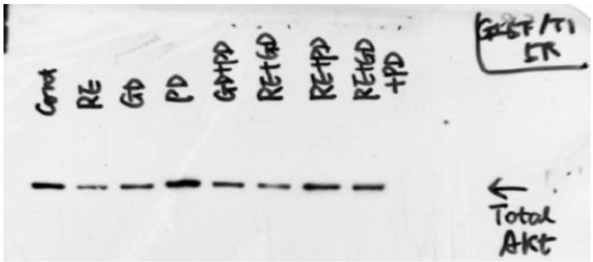
GIST/T1
5R
Cont
RE
GD
PD
GD+PD
RE+GD
RE+PD
RE+GD
+PD
Total
Akt

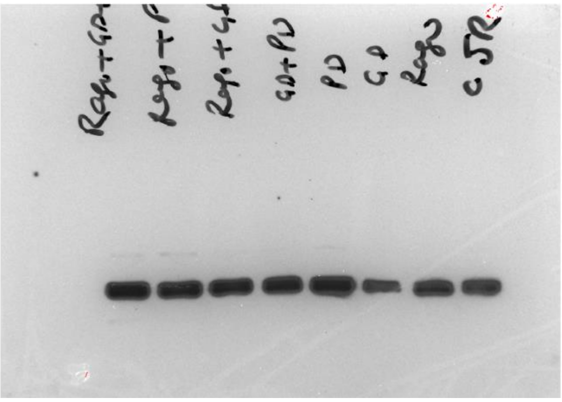

Total MAPK

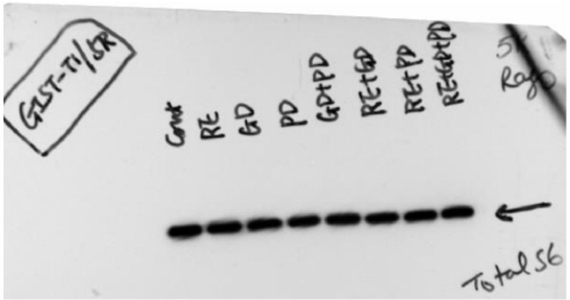

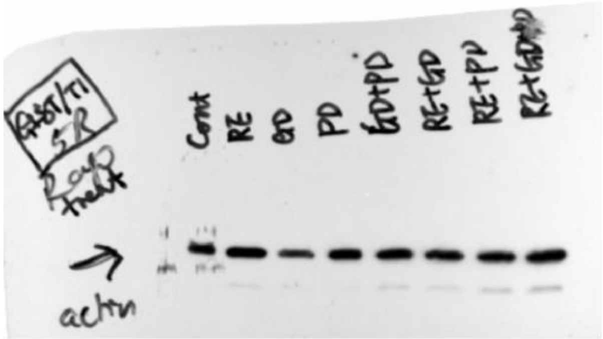

S3A Fig GIST-T1/10R-regorafenib

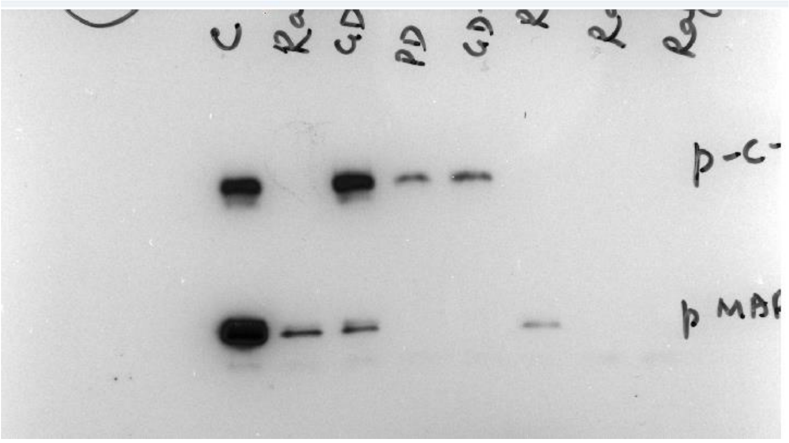

p-KIT

p-MAPK

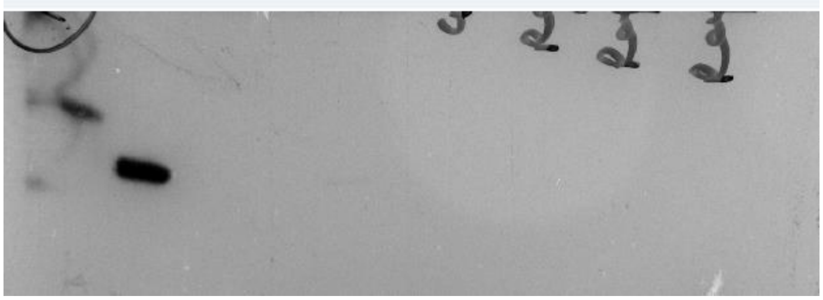

p-AKT

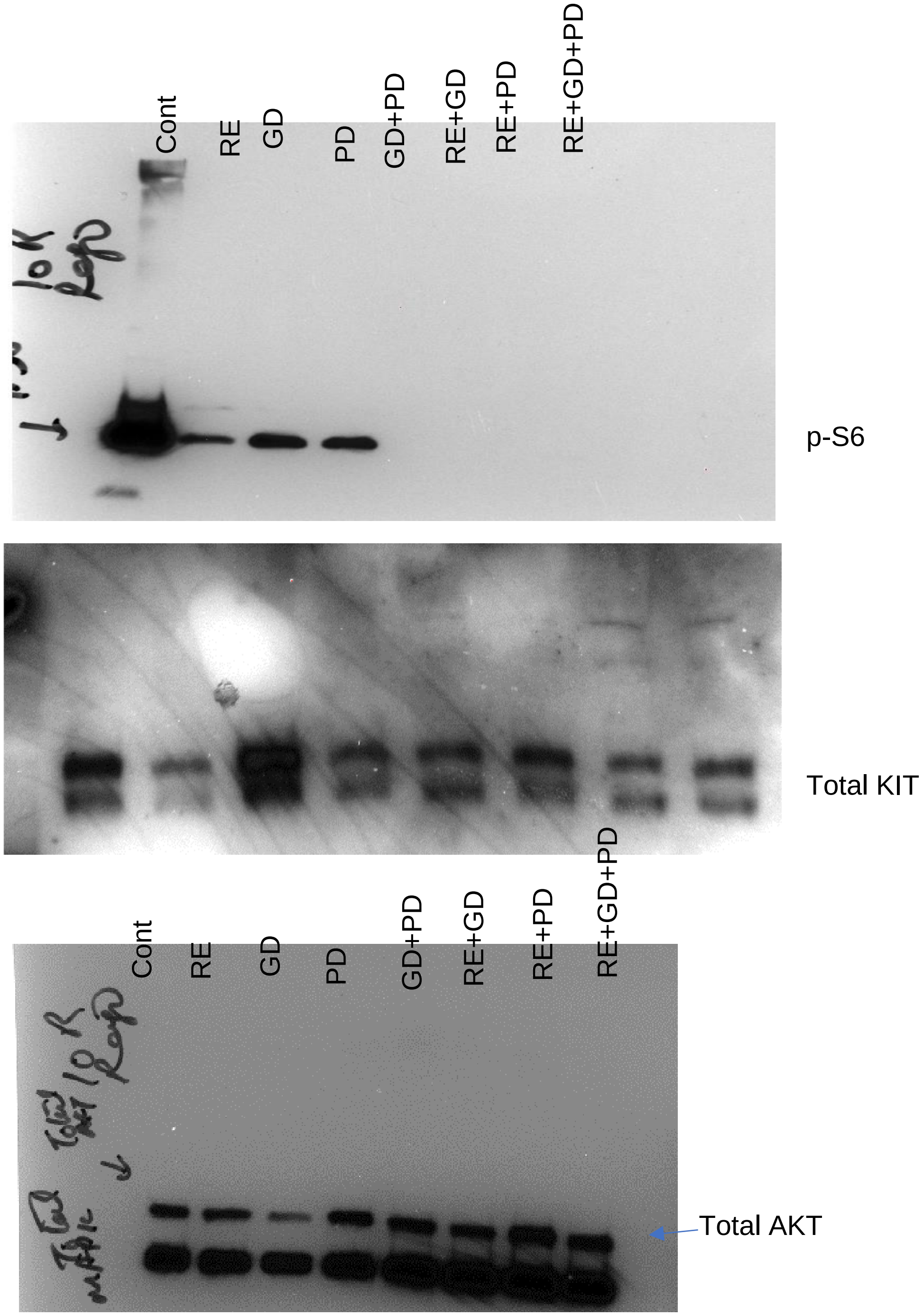

Cont
RE
GD
PD
GD+PD
RE+GD
RE+PD
RE+GD+PD
p-S6
Total KIT
Cont
RE
GD
PD
GD+PD
RE+GD
RE+PD
RE+GD+PD
Total AKT

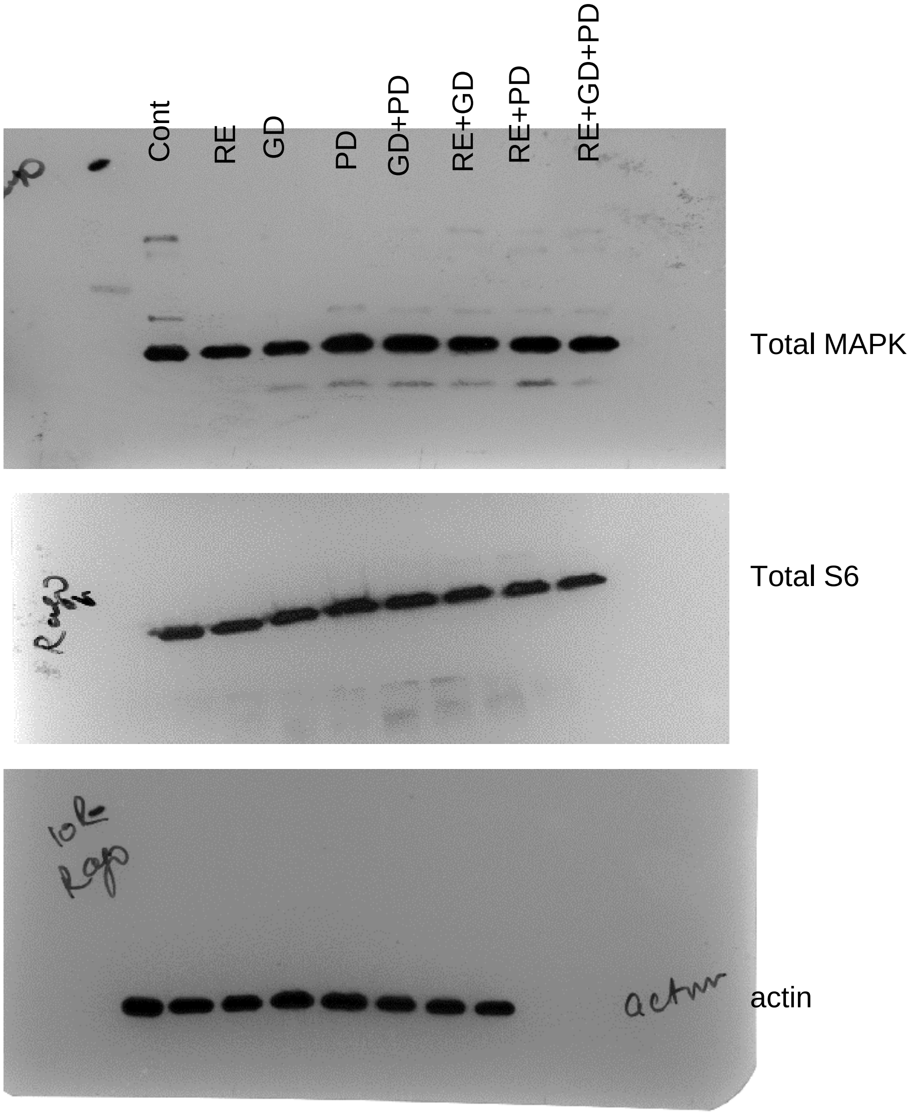

Fig. S3B--**GIST-T1/10R-Imatinib with trametinib**

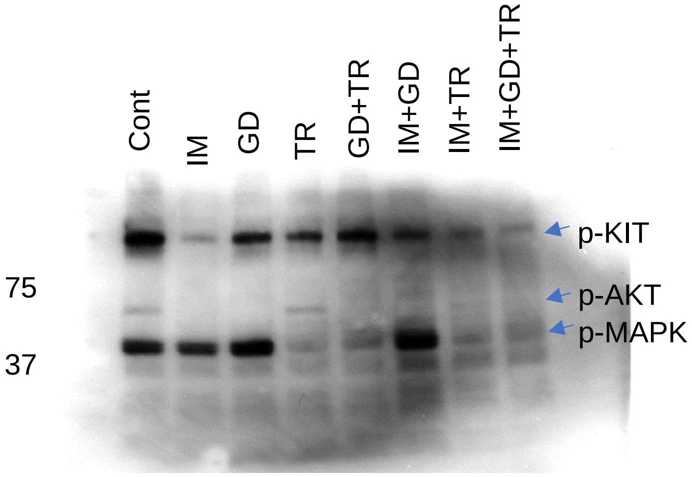

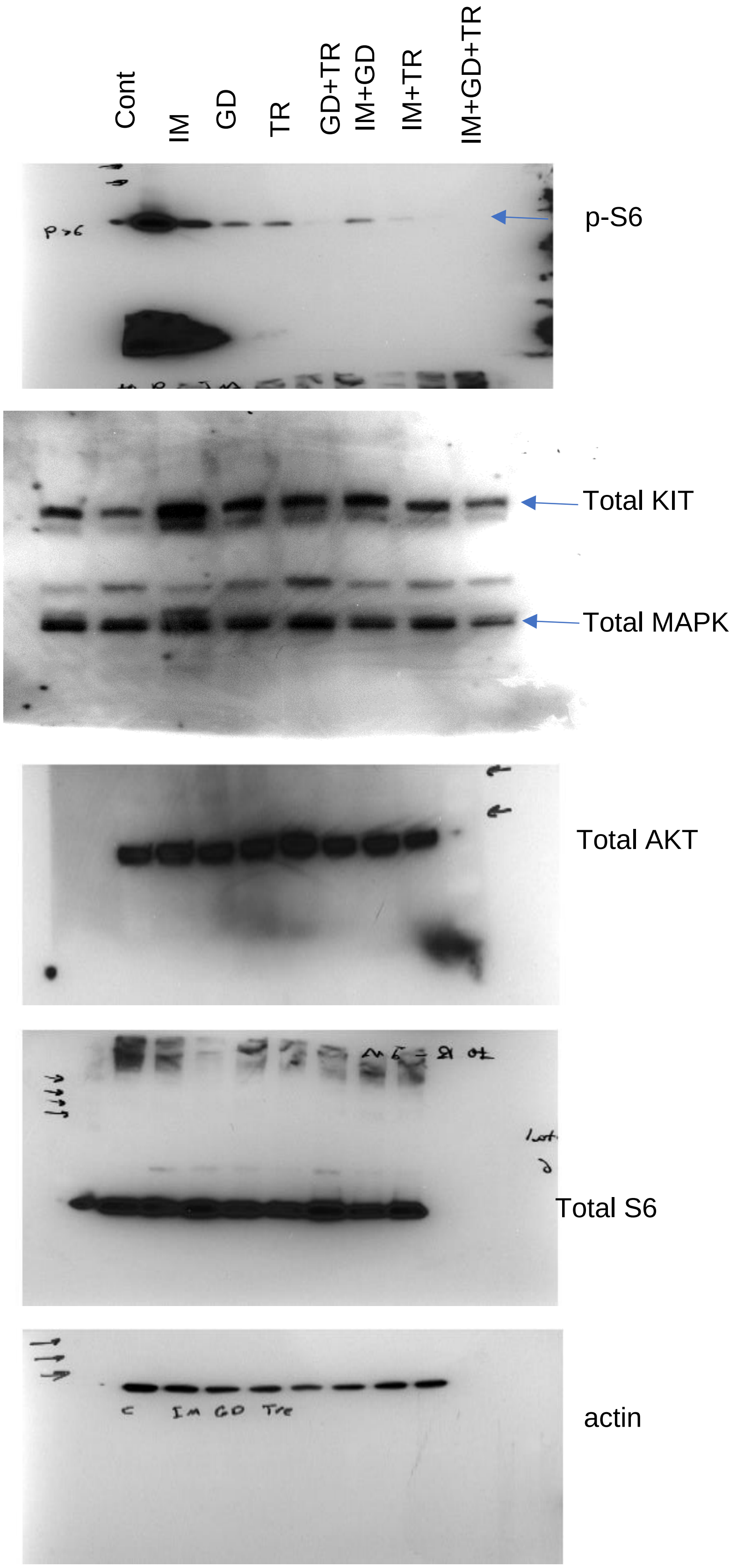
Cont
IM
GD
TR
GD+TR
IM+GD
IM+TR
IM+GD+TR
p-S6
Total KIT
Total MAPK
Total AKT
Total S6
actin

Fig. S3B

10R-sun with trametinib

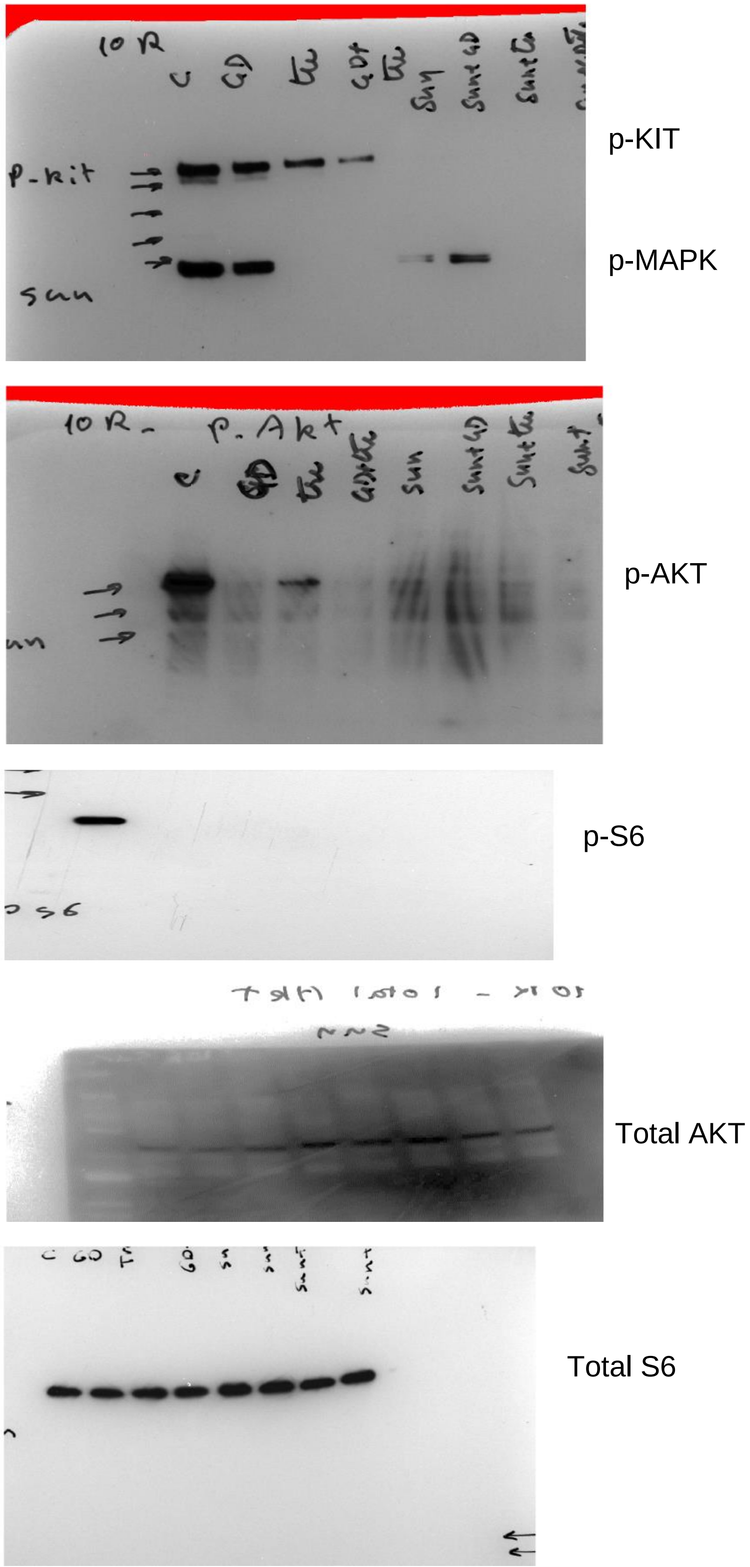

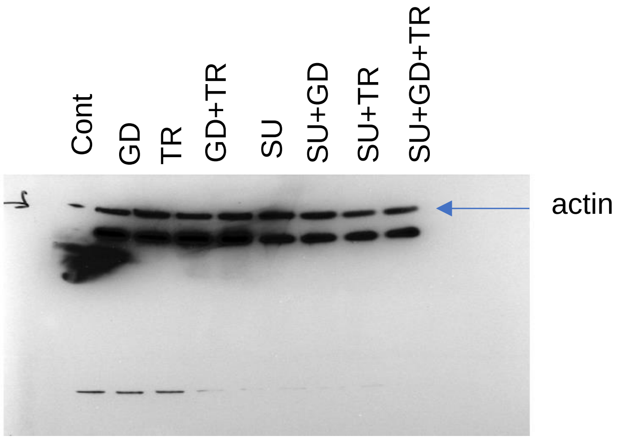

Fig. S3B- GIST-T1/10R-Rego with trametinib

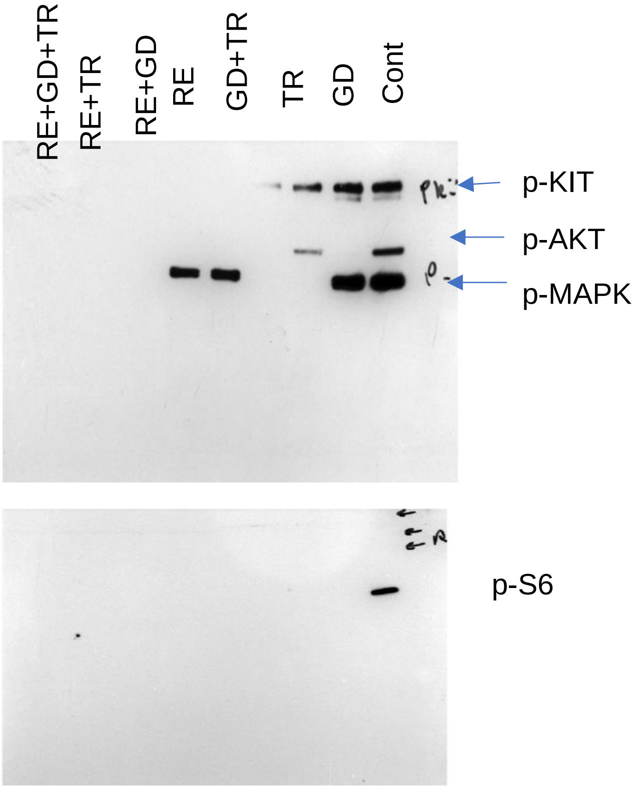

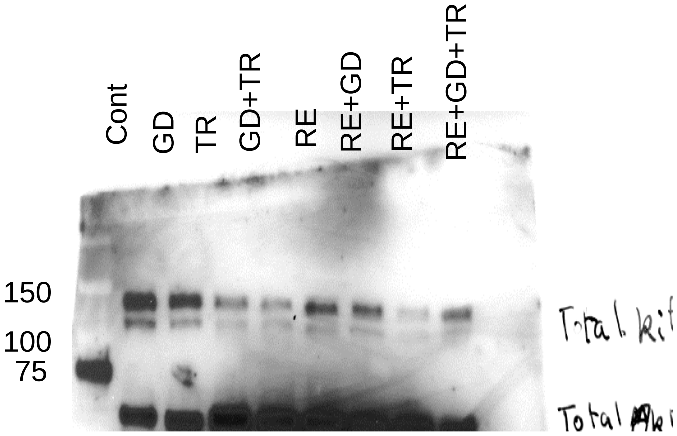

Total-KIT

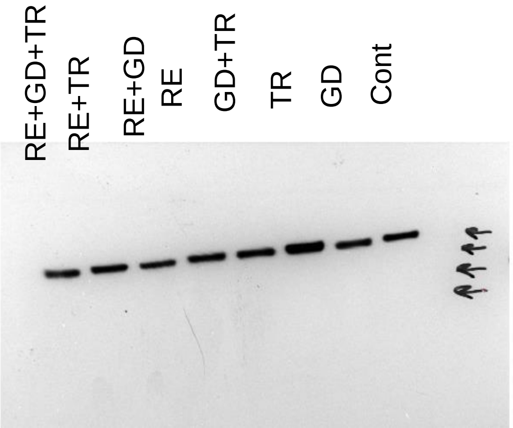

Total-AKT

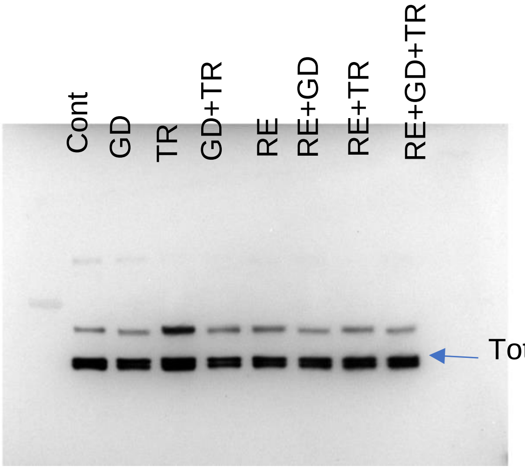

Total-MAPK

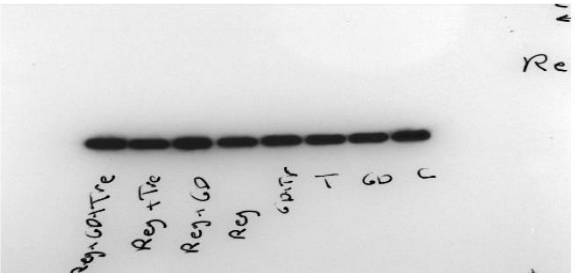

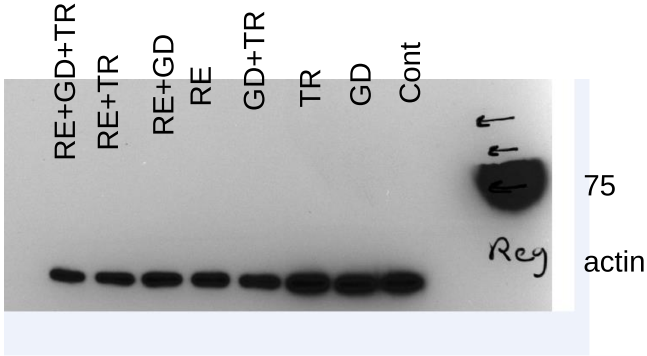

Fig. S4A

GIST-T1 _sunitinib

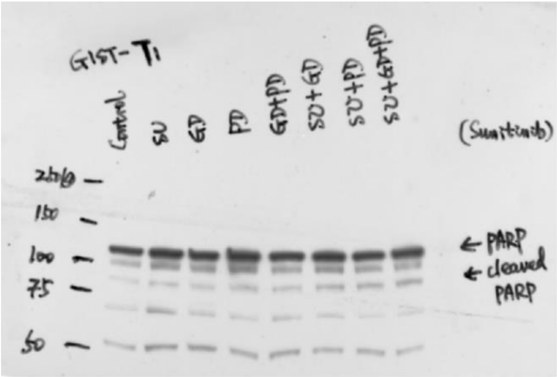

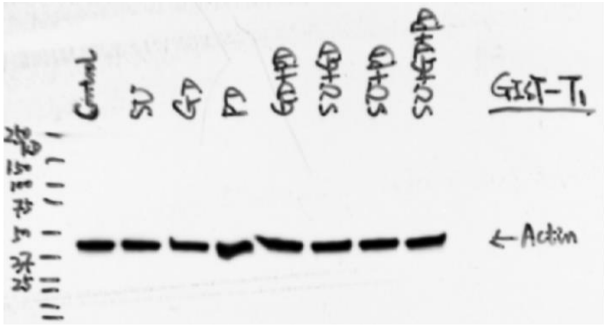

GIST-T1-regorafenib

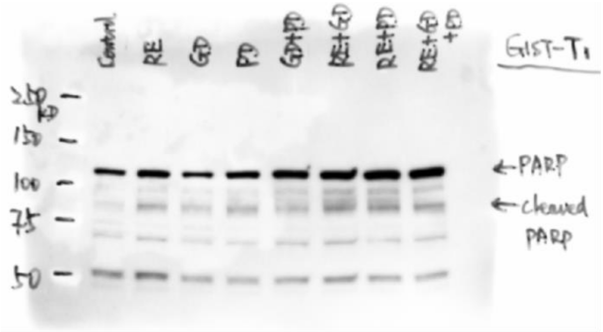

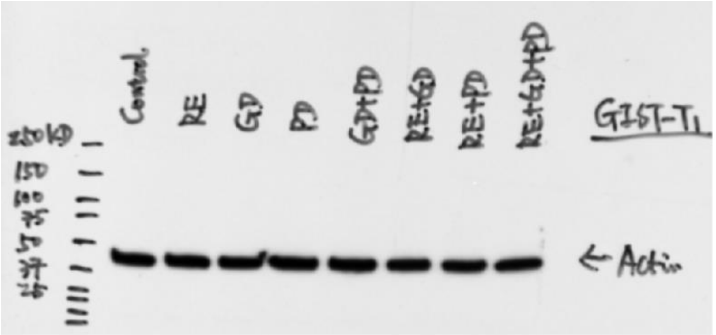

GIST-T1/670 (5R)--sunitinib

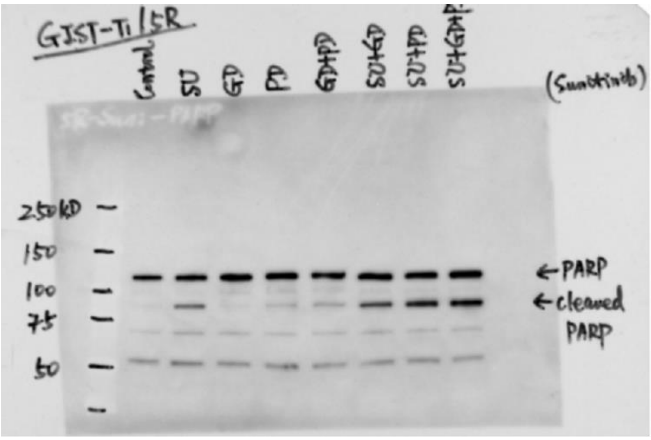

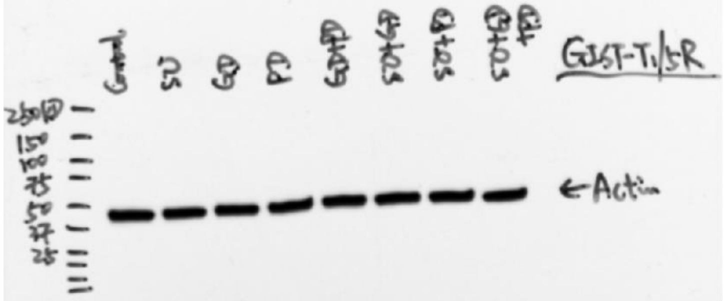

GIST-T1/670 (5R)--regorafenib

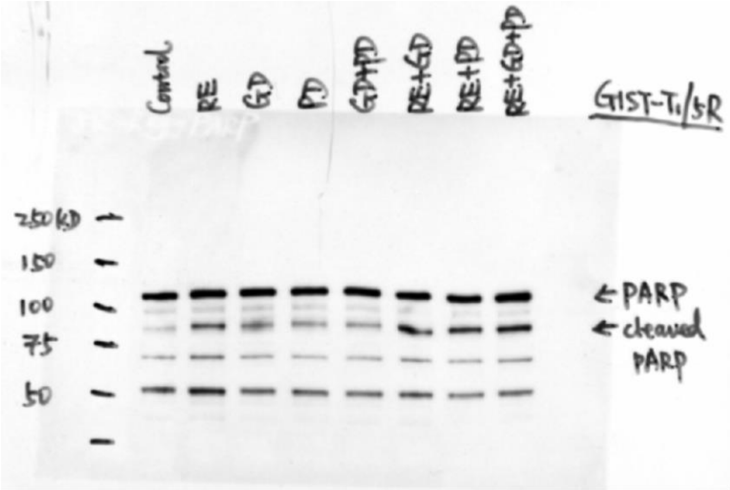

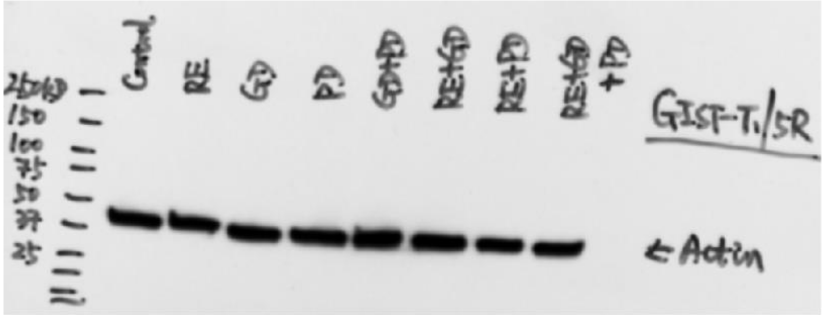

GIST-T1/10R--sunitinib

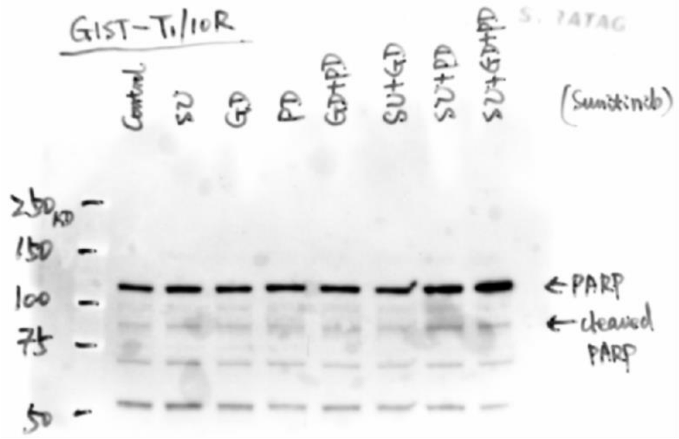

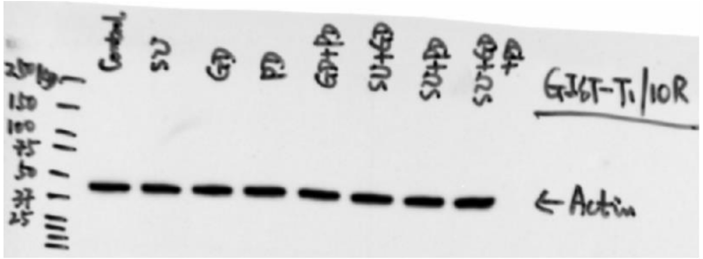

GIST-T1/10R--regorafenib

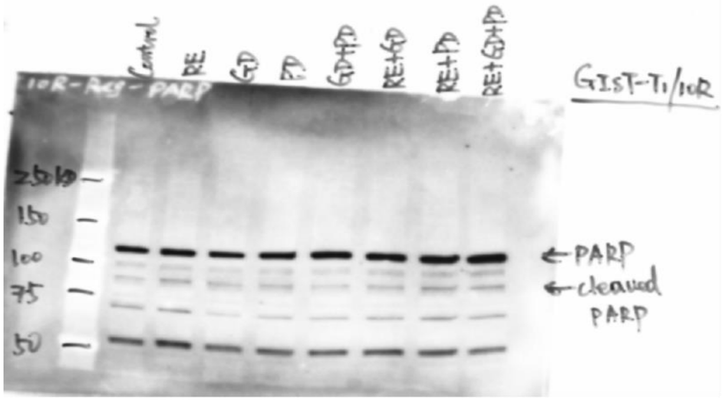

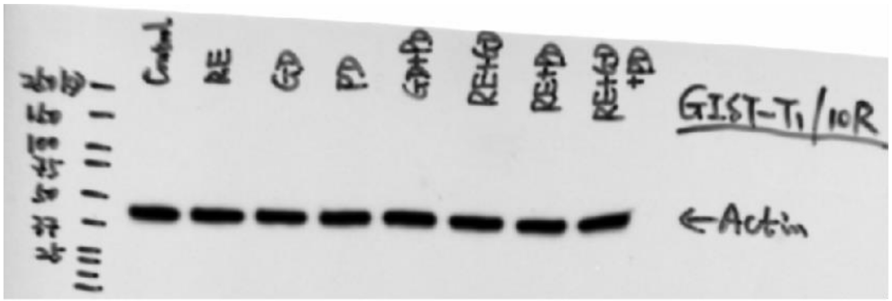

Supplement: S1 Raw images — (PDF) [file pone.0252689.s006.pdf]
